# Reactions of Nitroxides, Part 17. Synthesis, Fungistatic and Bacteriostatic Activity of Novel Five- and Six-Membered Nitroxyl Selenoureas and Selenocarbamates

**DOI:** 10.3390/molecules24132457

**Published:** 2019-07-04

**Authors:** Jerzy Zakrzewski, Bogumiła Huras, Anna Kiełczewska, Maria Krawczyk, Jarosław Hupko, Katarzyna Jaszczuk

**Affiliations:** Łukasiewicz Research Network-Institute of Industrial Organic Chemistry, Annopol 6, 03-236 Warsaw, Poland

**Keywords:** organoselenium compounds, selenoureas, selenocarbamates, fungistatic activity, bacteriostatic activity

## Abstract

The reactions of 3-isoselenocyanato-2,2,5,5-tetramethylpyrrolidine-1-oxyl, 3-isoselenocyanatomethyl-2,2,5,5-tetramethyl-3-pyrrolidine-1-oxyl, and 4-isoselenocyanato-2,2,6,6-tetramethylpiperidine-1-oxyl with selected amines and alcohols give the corresponding novel nitroxyl selenoureas and selenocarbamates, all bearing a nitroxyl moiety. Synthesized selenoureas and selenocarbamates show significant activity against pathogenic fungi and bacteria. In contrast to piperidine nitroxides, pyrrolidine, five-membered nitroxyl selenoureas and selenocarbamates show excellent antifungal and antibacterial activity against pathogenic fungi and bacteria, respectively.

## 1. Introduction

In our previous paper [1], we synthesized nitroxyl radicals containing a tellurium atom and evaluated their antifungal activity. As a part of our continuing interest in the synthesis and evaluation of the biological activity of the compounds containing chalcogen atoms, the activity of organoselenium compounds bearing nitroxyl moieties is discussed in the present paper.

The role of organoselenium compounds in organic synthesis, their presence in living organisms, and application as bioactive compounds, have been recently discussed [2]. The biological activity of synthetic organoselenium compounds was reviewed [3,4,5,6,7,8,9]. 

Selenoureas showed free-radical scavenging [10,11,12], and enzyme inhibition [10,11,13,14,15,16] potential. They demonstrated anticancer [10,11,17], DNA binding [17,18,19,20], antioxidant [17,18,21,22,23], antibacterial [18], antifungal [18,24,25,26], and herbicidal [25] properties.

Selenourea derivatives were synthesized from the following starting compounds—sources of selenium: isoselenocyanates and primary or secondary amines [27,28,29,30,31,32,33,34,35,36,37,38,39,40,41,42,43,44] selenoamides and nitrile oxides (generated in situ) [45], hydrogen selenide [46,47,48], lithium aluminum hydride (LiAlH_2_Se) [12,32,36,49,50,51,52,53], bis(dimethylaluminum) selenide [54], tetraethylammonium tetraselenotungstate [14], elemental selenium with an isonitrile and an amine [55], elemental selenium with an amine and triethylorthoformate [56], and elemental selenium with a secondary amine, a base, and dihalomethan derivatives [57,58]. 

Selenoureas served as starting materials to the synthesis of further organoselenium derivatives [59]. Selenocarbamates showed antiproliferative [60], cytotoxic [61], antioxidant [22], pesticidal [24,26], and effective superoxide anion scavenger [62] activities. Selenocarbamates were obtained by addition reaction of isoselenocyanate with alcohols [54,63].

Methyl and ethyl nitroxyl selenocarbamates were obtained from 4-isoselenocyanato-2,2,6,6-tetramethylpiperidine-1-oxyl, by the reaction with either sodium methoxide or ethoxide, in methanol or ethanol, respectively [26]. The biological action of nitroxyl radicals was well documented [64,65,66,67]. Nitroxides showed antioxidant properties by scavenging free radical reactive oxygen species (ROS) and, in consequence, protecting cells against oxidative stress [64,65,66,67,68,69,70]. Antioxidant and antitumor activity of amides obtained from exactly the same nitroxyl amines (PROXYL-NH_2_, PROXYL-CH_2_NH_2_, and TEMPO-NH_2_) as used in this work have been recently described [71].

Nitroxides acted as the enzyme superoxide dismutase mimics, converting superoxide anion to oxygen and hydrogen peroxide using redox reactions involving a nitroxide, a corresponding hydroxylamine and an oxoammonium salt [65]. It was, however, stated that nitroxides were less active than superoxide dismutase itself [72].

Radioprotective effects of nitroxides in the presence of iron ions were detected [73]. Radioprotective effects in vivo of 4-hydroxy-2,2,6,6-tetramethyl-piperidine-1-oxyl (TEMPOL) were studied in mice [74]. Ebselen (2-phenyl-1,2-benzisoselenazol-3(*2H*)-one) derivatives modified with a nitroxyl radical fragment (see below) showed higher activity as glutathione peroxidase mimic than ebselene itself [75]. Antihypertensive effects observed for nitroxides (mainly TEMPOL) were reviewed [76]. The inhibitory effect of selected dinitroxides and polynitroxides on the growth of some species of bacteria, yeasts and fungi was described [77].

The role of nitroxides in cancer therapy connected with their antioxidant properties was reviewed [66,78]. Free nitroxyl radicals bearing an adamantyl moiety exhibited also the biological activity. Adamantyl derivatives of nitroxyl radicals showed antiparkinsonian activity [79]. In one of our previous work [26] fungicidal activities of six-membered nitroxyl selenoureas and selenocarbamates were presented. Herein, we would like to present the synthesis and pesticidal properties of five- and six-membered nitroxyl selenoureas and selenocarbamates.

## 2. Results and Discussion

### 2.1. Synthesis of Selenoureas ***4***–***8*** and Selenocarbamates ***9***, ***10***

**Scheme 1:**
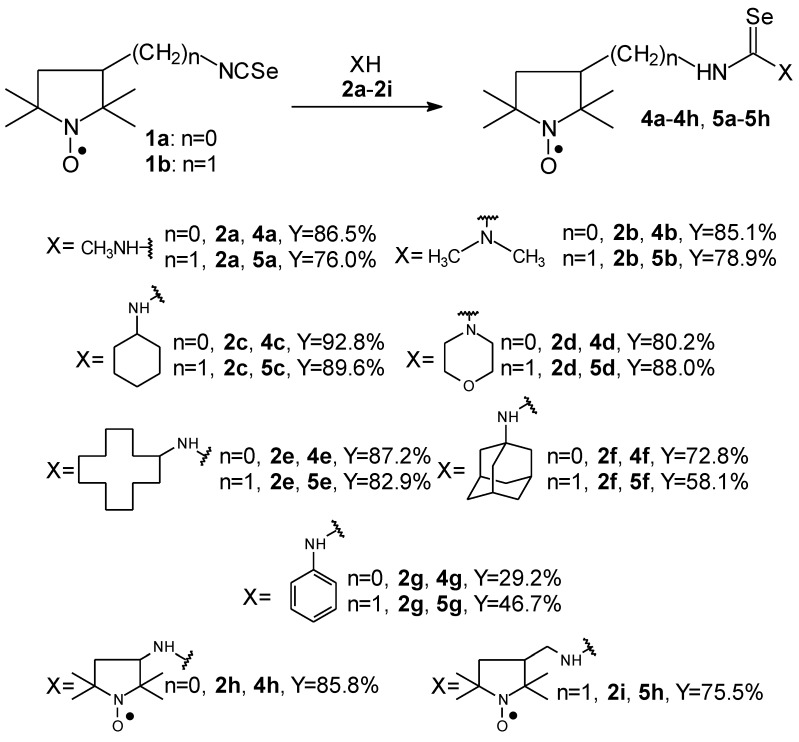
Selenoureas **4a**–**4h** and **5a**–**5h**; the reaction of 3-isoselenocyanato-2,2,5,5-tetramethylpyrrolidine-1-oxyl (**1a**, PROXYL-NCSe) and 3-isoselenocyanatomethyl-2,2,5,5-tetramethylpyrrolidine-1-oxyl (**1b**, PROXYL-CH_2_NCSe) with a series of amines.

Selenoureas **4a**–**4h** and **5a**–**5h** were synthesized by addition reaction of a series of amines **2a**–**2i** to the five-membered nitroxyl isoselenocyanates: 3-isoselenocyanato-2,2,5,5-tetramethylpyrrolidine-1-oxyl (**1a**, PROXYL-NCSe), and 3-isoselenocyanatomethyl-2,2,5,5-tetramethylpyrrolidine-1-oxyl (**1b**, PROXYL-CH_2_NCSe). The reactions occurred at room temperature (at approximately 0–10 °C in the case of volatile amines). Benzene was used as a solvent (Scheme 1).

**Scheme 2:**
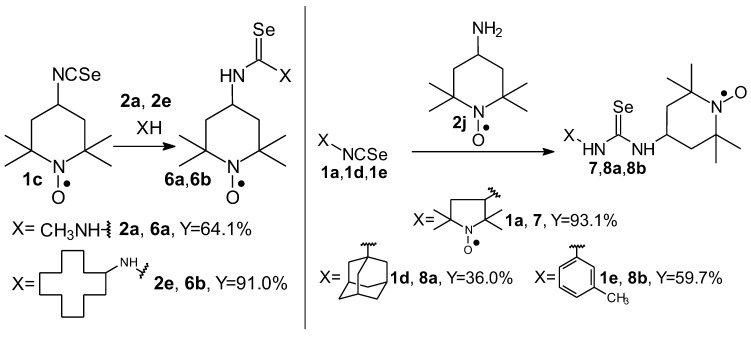
Selenoureas containing six-membered nitroxyl moiety **6a**, **6b**, **7**, **8a**, **8b**; reaction of 4-isoselenocyanato-2,2,6,6-tetramethylpiperidine-1-oxyl (**1c**, TEMPO-NCSe), 3-isoselenocyanato-2,2,5,5-tetramethylpyrrolidine-1-oxyl (**1a**, PROXYL-NCSe) 1-adamantyl isoselenocyanate (**1d**), 3-methylphenyl isoselenocyanate (**1e**) with a series of amines.

Selenoureas **6a** and **6b** were synthesized by addition reaction of methylamine (**2a**) and cyclododecylamine (**2e**) to the six-membered nitroxyl isoselenocyanate: 4-isoselenocyanato-2,2,6,6-tetramethylpiperidine-1-oxyl (**1c**, TEMPO-NCSe). Biradical selenourea **7** as well as selenoureas **8a** and **8b** were synthesized by addition reaction of the six-membered nitroxyl amine: 4-amino-2,2,6,6-tetramethylpiperidine-1-oxyl (**2j**, TEMPO-NH_2_) to the five-membered nitroxyl isoselenocyanate: 3-isoselenocyanato-2,2,5,5-tetramethylpyrrolidine-1-oxyl (**1a**, PROXYL-NCSe) as well as adamantyl and 3-methylphenyl isoselenocyanates (**1d** and **1e**, respectively). The reactions occurred at room temperature. Benzene was used as a solvent (Scheme 2).

**Scheme 3:**
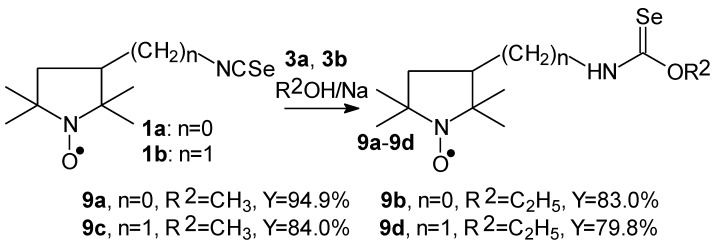
Selenocarbamates **9a**–**9d**; the reaction of 3-isoselenocyanato-2,2,5,5-tetramethylpyrrolidine-1-oxyl (**1a**, PROXYL-NCSe) and 3-isoselenocyanatomethyl-2,2,5,5-tetramethylpyrrolidine-1-oxyl (**1b**, PROXYL-CH_2_NCSe) with methanol and ethanol.

Selenocarbamates **9a**–**9d** were synthesized by addition reaction of methanol (**3a**) or ethanol (**3b**) to the five-membered isoselenocyanates: 3-isoselenocyanato-2,2,5,5-tetramethylpyrrolidine-1-oxyl (**1a**, PROXYL-NCSe), and 3-isoselenocyanatomethyl-2,2,5,5-tetramethylpyrrolidine-1-oxyl (**1b**, PROXYL-CH_2_NCSe). The reaction occurred at room temperature. A corresponding alcohol was used as a solvent (Scheme 3).

**Scheme 4:**
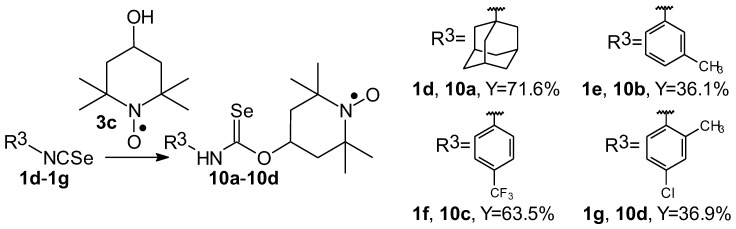
Selenocarbamates **10a**–**10d**; the reaction of 1-adamantyl isoselenocyanate (**1d**), and aryl isoselenocyanates (**1e**–**1g**) with 4-hydroxy-2,2,6,6-tetramethyl-piperidine-1-oxyl (**3c**, TEMPOL).

Selenocarbamates containing six-membered nitroxyl moiety **10a**–**10d** were synthesized by addition reaction of nitroxyl secondary alcohol: 4-hydroxy-2,2,6,6-tetramethylpiperidine-1-oxyl (**3c**. TEMPOL) to the 1-adamantyl isoselenocyanate (**1d**) and aryl isoselenocyanates: 3-methylphenyl, 4-trifluoromethylphenyl, and 2-methyl-4-chlorophenyl isoselenocyanates (**1e**, **1f**, **1g**, respectively) in the presence of NaH as a base. The reaction occurred at room temperature. THF was used as a solvent (Scheme 4).

The predominant amount of synthesized nitroxyl selenoureas and selenocarbamates proved to be unstable at elevated temperatures. Compounds generally underwent decomposition upon heating during the melting temperature measurement. As a result of the decomposition, wide ranges of melting temperatures were observed. The purity of the synthesized compounds was evaluated by means of HPLC. 

All the synthesized compounds were characterized using mass spectrometry (EI MS, ESI MS, HR MS) and IR spectroscopy (see the Supplementary Materials). The ^1^H NMR spectra were not performed due to the paramagnetic broadening, owing to the presence of the nitroxyl moieties [80,81,82,83,84]. EI MS showed the presence of the molecular mass peak for the almost all compounds under investigation (except six membered nitroxides **8a** and **8b**). The intensities of the molecular signals were diverse. The molecular signals were abundant for five-membered nitroxyl selenoureas **5b**–**5d** and selenocarbamates **9a** and **9c**. The intensities of molecular signals were 100% for a six-membered nitroxyl selenourea **6a** and a nitroxyl selenocarbamate **9d**. Five-membered nitroxyl selenoureas **4f**, **5f**, **5h**, six-membered nitroxyl selenoureas **6b**, **8a**, **8b**, and nitroxyl selenocarbamates **10a**–**10c** showed negligible molecular signals (for **8a**, **8b** no visible molecular signal were observed). 

In order to confirm the molecular mass, ESI MS was performed for the almost all compounds under investigations (except of a selenourea **4b** and selenocarbamates **9a** and **10a**–**10c**). The molecular masses were confirmed by the observation *m*/*z* M + 23 (100%) signals. 

Exact molecular masses were confirmed by means of HR ESI MS (HR EI MS in the case of selenourea **4b** and selenocarbamates **9a** and **10a**–**10c**). 

IR spectra revealed strong absorption II amide band at ~1550 cm^−1^ characteristic for selenoureas [42].

### 2.2. Fungistatic and Bacteriostatic Activity of Selenoureas ***4***–***8*** and Selenocarbamates ***9***,***10***

All synthesized selenoureas **4**–**8** and selenocarbamates **9**, **10** were tested for the herbicidal, insecticidal, acaricidal, antifungal, and antibacterial activities. No herbicidal, insecticidal, and acaricidal activities were observed. Significant fungistatic and bacteriostatic activities were found.

The investigated selenium containing nitroxides **4**–**8** and **9**, **10** were tested in vitro against the basic set of phytopathogenic fungi: *Botrytis cinerea*, *Fusarium culmorum*, *Phytophthora cactorum*, and *Rhizoctonia solani* at the concentration of 200 mg/L, and for the selected, active compounds, at the concentration of 20 mg/L. The selenoureas **4**–**8** and selenocarbamates **9**, **10** were also tested in vivo against *Blumeria graminis* as a phytopathogenic fungi, however, no tested compounds showed satisfactory activity against this species.

In order to enlarge the set of phytopathogenic fungi, the selected, active selenouraeas **4**–**8** and selenocarbamates **9**, **10** were also tested at the concentration of 20 mg/L against the phytopathogenic fungi *Alternaria alternata, Fusarium oxysporum, Phytophtora infestans*, and against *Ascosphaera apis* (causing chalkbrood disease in honey bees). 

The bacteriostatic activity of selenoureas **4**–**8** and selenocarbamates **9**, **10** was tested for phytopathogenic bacteria *Erwinia carotovora sub. atraseptica*, *Pseudomonas phaseolicola*, *Pseudomonas lachrymans*, *Pseudomonas syringae* at concentration of 100 mg/L. The results of the fungistatic activity were presented in Table 1 and Table 2 The results of the bacteriostatic activity were presented in Table 3 and Table 4.

Almost all nitroxyl selenoureas **4**–**8** (except **4e**—cyclododecyl derivative—see below) showed 100% activity against at least one fungus of the basic set of the tested fungi at the basic concentration of 200 mg/L.

-**4b**, **4d**, **5b**, **5c**, **5d**, **5h**, **6b**, **7** were active at 100% level against four basic fungi.

-**4a**, **4c**, **4h**, **5a**, **5g**, **6a**, were active against three of four basic fungi at 100% level.

-**4f**, **4g**, **8b** were active against two of four basic fungi at 100% level.

-**5e**, **5f** were active against one of four basic fungi at 100% level.

Especially, both five-membered nitroxyl selenourea series **4** i **5** showed the high activity against *B.cinerea*, *P.cactorum*, and *R.solani* at the concentration of 200 mg/L.

However, it was worthy to note that the cyclododecyl and adamantyl nitroxyl selenoureas revealed significantly lower activity:

-cyclododecyl and adamantyl nitroxyl selenoureas **4e**, **4f**, **5e**, **5f** against *B. cinerea*,

-cyclododecyl nitroxyl selenourea **4e** i adamantyl nitroxyl selenourea **5f** against *P.cactorum,*

-cyclododecyl nitroxyl selenoureas **4e** i **5e** against *R.solani*.

Nitroxyl carbamates **9a**–**9d** were active at 100% level against all basic tested species *B.cinerea*, *F.culmorum P.cactorum*, and *R.solani* at the basic concentration of 200 mg/L. Nitroxyl selenocarbamates **10a**–**10d** (TEMPOL (**3c**) derivatives) showed neither fungicidal nor bacteriostatic activity.

As noted above almost all “100%” compounds at the basic concentration of 200 mg/L were tested against the basic set of fungi (*B. cinerea*, *F. culmorum*, *P. cactorum*, and *R. solani*) at the concentration of 20 mg/L and against the additional set of fungi (*A. alternata, F. oxysporum, P. infestans*, and *A. apis*) also at the concentration of 20 mg/L. No tested nitroxyl compounds attained 100% in tests with the additional set of fungi. However, against the basic set of fungi significant amount of the tested nitroxyl selenoureas were active also at the concentration of 20 mg/L at the same 100% level. Nitroxyl selenourea **4c** was active at the concentration of 20 mg/L at 100% level (MIC ≤ 20) against two species: *B. cinerea and P. cactorum*. Nitroxyl selenoureas **4d**, **4h**, **5a**–**5d**, **5g**, **5h**, **7** were active at the concentration of 20 mg/L at 100% level (MIC ≤ 20) against *P. cactorum*.

The different size of alkyl and cycloalkyl fragments present in the nitroxyl selenoureas **4**–**8**, prompted us to estimate the potential correlation between the observed fungicidal activity and the calculated octanol-water partition coefficient (clog P, HyperChem 7 software, Hypercube Inc., Gainesville, Fl, USA). Linear dependence between the average fungicidal activity (at 200 mg/L) vs. clog P was observed for the series of five-membered nitroxides **4a**–**4g** (R^2^ = 0.95). Interestingly, the analogous dependence for the similar series **5a**–**5g** was not observed.

Nitroxyl selenoureas **4c**, **5b**, **5c**, **5h**, **6a**, **6b** showed activity at the concentration of <100 mg/L for all four bacterial species.

**5g** showed activity at concentration of <100 mg/L against three of four bacteria species. 

**4a**, **4d**, **4g**, **5f** showed activity at concentration of <100 mg/L against two of four bacteria species.

**4f** showed activity at concentration of <100 mg/L against one of four bacteria species.

Nitroxyl selenocarbamate **9b** showed activity at concentration of <100 mg/L against three of four tested bacteria species. Nitroxyl selenocarbamate **10d** showed activity at concentration of <100 mg/L against one of four tested bacteria species. 

## 3. Materials and Methods 

### 3.1. General

The synthesis of the following nitroxyl, cycloalkyl and aryl isoselenocyanates: 3-isoselenocyanato-2,2,5,5-tetramethylpyrrolidine-1-oxyl (PROXYL-NCSe, **1a**) and 3-isoselenocyanatomethyl-2,2,5,5-tetramethylpyrrolidine-1-oxyl (PROXYL-CH_2_NCSe, **1b**), 1-adamantyl isoselenocyanate (**1d**), 3-methylphenyl isoselenocyanate (**1e**), 4-trifluoromethylphenyl isoselenocyanate (**1f**), and 4-chloro-2-methylphenyl isoselenocyanate (**1g**) has been recently described [85], The synthesis of 4-isoselenocyanato-2,2,6,6-tetramethylpiperidine-1-oxyl (TEMPO-NCSe, **1c**) was described previously [26,85,86,87]. The references for the synthesis of the following nitroxyl amines: 3-amino-2,2,5,5-tetramethylpyrrolidine-1-oxyl, PROXYL-NH_2_ (**2h**), 3-aminomethyl-2,2,5,5-tetramethylpyrrolidine-1-oxyl PROXYL-CH2-NH_2_ (**2i**), 4-amino-2,2,6,6-tetramethylppiperidine-1-oxyl, TEMPO-NH_2_ (**2j**) have been recently cited [85]. The references for the synthesis of 4-hydroxy-2,2,6,6-tetramethylppiperidine-1-oxyl (TEMPOL (**3c**)) was cited [88]. TLC was carried out on silica gel Merck Alurolle 5562 or Alufolien 5554; TLC visualization was achieved using UV 254 nm light and/or I_2_ vapor; visualization of selenium containing compounds: UV 254 nm and irradiation with UV lamp for 5–10 min (red spots) or spraying with 1% ethanolic PdCl_2_ (dark brown spots on pale beige background). Column chromatography was performed on silica gel 0.040–0.063 mm, 230–400 mesh: Merck 1.09385.1000 or Zeochem 60 hyd. HPLC conditions: C18, 5 μ, 150 × 4.6 mm, UV detector, λ = 220 nm; method a: mobil phase: acetonitrile:H_2_O 1:1, flow: 1 mL/min; method b: mobil phase: acetonitrile:H_2_O 1:3, flow: 1 mL/min; method c: mobil phase: acetonitrile:H_2_O 3:1, flow: 1.3 mL/min. EI-MS data (70 eV) were recorded on an AMD 604 and Agilent Technologies 5975 B mass spectrometers. HRMS-EI data were recorded by using an AMD 604 mass spectrometer. MS-ESI and HRMS-ESI (MeOH as a solvent) were recorded by using a Micromass LCT apparatus. IR spectra were recorded on an FT/IR Jasco 420 spectrophotometer. 

### 3.2. 1,3-Substituted Nitroxyl Selenoureas ***4a, 4b, 5a, 5b, 6a*;** Reaction of the Nitroxyl Isoselenocyanates ***1a***–***1c*** with Volatile Amines ***2a***, ***2b***; a General Procedure

To a chilled and magnetically stirred solution of the corresponding nitroxyl isoselenocyanate (**1a**–**1c**, 0.001 mol) in benzene (5–6 mL), benzene solution of methylamine (**2a**) or dimethylamine (**2b**) (~1 mL) was added dropwise at about 5 °C. The reaction was carried out for 1 h at room temperature. The precipitate was filtered off and washed with hexane. The filtrate was evaporated, the residue was triturated with hexane to give the additional amount of the product (Scheme 1).

### 3.3. 1,3-Substituted Nitroxyl Selenoureas ***4c***–***4h*, *5c***–***5h*, *6b*, *7*, *8a*, *8b***; Reaction of the Nitroxyl Isoselenocyanates ***1a***–***1c*** with Liquid and Solid Amines (***2c***–***2j***); a General Procedure

A corresponding nitroxyl isoselenocyanate (**1a**–**1c**, 0.001 mol) was dissolved in benzene (5–6 mL). The corresponding amine (**2c**–**2j**, 0.0011 mol) was added using a syringe. The reagents were stirred for 1 h at room temperature. The formed precipitate was filtered off and washed with hexane. If no precipitate was formed, the solution was evaporated. The residue was either triturated with hexane and filtered off or chromatographed (Scheme 1 and Scheme 2).

*1-(2,2,5,5-Tetramethyl-1-oxyl-3-pyrrolidinyl)-3-methyl selenourea* (**4a**). 
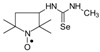
 C_10_H_20_N_3_OSe, M = 278, 0.32 g, Yield: 86.5%, yellow glass, m.p. 141–144 °C (dec.), TLC: *R*_f_ = 0.14 benzene:methanol 9:1; purity (HPLC, method b): 98.2%; MS (EI, 70 eV, *m*/*z*, int [%]): 279 (13), 278 (20, M), 276 (12), 262 (10), 260 (43), 258 (22), 248 (25), 246 (24), 244 (11), 233 (10), 205 (50), 203 (29), 192 (25), 190 (14), 177 (22), 175 (11), 167 (12), 165 (35), 163 (20), 139 (13), 138 (10), 137 (10), 136 (12), 126 (30), 125 (21), 124 (38), 123 (15), 122 (28), 120 (14), 111 (92), 110 (74), 100 (27), 98 (54), 95 (59), 84 (100), 70 (33), 69 (29), 67 (21), 57 (66), 56 (59), 55 (28), 53 (12), 42 (30), 41 (39); MS (ESI, *m*/*z*, int [%]): 301 (100, M + 23), 299 (20); HR MS (ESI, *m*/*z*) for C_10_H_20_N_3_OSeNa: calcd: 301.0669, found: 301.0656; IR (ν, cm^−1^, KBr): 3420, 3280, 2971, 1564.

*1-(2,2,5,5-Tetramethyl-1-oxyl-3-pyrrolidinyl)-3,3-dimethyl selenourea* (**4b**). 
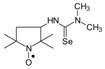
 C_11_H_22_N_3_Ose, M = 292, Yield: 85.1%, yellow crystalline powder, m.p. 178–180 °C (dec.), TLC: *R*_f_ = 0.11 benzene:methanol 9:1; purity (HPLC, method b): 94.6%; MS (EI, 70 eV, *m*/*z*, int [%]): 292 (14, M), 290 (8), 262 (15), 260 (14), 247 (10), 245 (7), 221 (11), 219 (53), 217 (32), 206 (22.6), 205 (9), 204 (11), 203 (8), 193 (22), 191 (37), 189 (14), 179 (37), 177 (21), 153 (11), 138 (21), 136 (88), 134 (43), 126 (28), 125 (30), 124 (37), 111 (12), 110 (31), 100 (53), 98 (40), 95 (34), 82 (19), 71 (100), 69 (30), 67 (23), 56 (34), 55 (39), 44 (54), 42 (45), 41 (45); HR MS (EI, 70 eV, *m*/*z*) for C_11_H_22_N_3_OSe: calcd: 292.09281, found: 292.09388; IR (ν, cm^−1^, KBr): 3440, 3344, 2973, 1557, 1341.

*1-(2,2,5,5-Tetramethyl-1-oxyl-3-pyrrolidinyl)-3,3-pentyleno selenourea* (**4c**). 
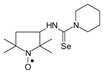
 C_14_H_26_N_3_OSe, M = 332, 0.399 g, Yield: 92.8%, yellow crystalline powder, m.p. 154–156 °C (dec.), TLC: *R*_f_ = 0.25 benzene:methanol 9:1; purity (HPLC, method a): 98.8%; MS (EI, 70 eV, *m*/*z*, int [%]): 333 (6), 332 (9, M), 330 (5), 302 (11), 300 (19), 287 (10), 285 (6), 259 (76), 257 (39), 246 (18), 233 (33), 231 (32), 219 (19), 176 (95), 174 (47), 165 (55), 164 (31), 136 (12), 126 (14), 124 (32), 111 (58), 110 (30), 100 (27), 98 (47), 95 (21), 84 (100), 69 (61), 67 (19), 56 (41), 55 (40), 41 (63); MS (ESI, *m*/*z*, int [%]): 355 (100, M + Na), 353 (10); HR MS (ESI, *m*/*z*) for C_14_H_26_N_3_OSeNa: calcd: 355.1139, found: 355.1154; IR (ν, cm^−1^, KBr): 3359, 2960, 2938, 1550, 1331, 1239, 1132.

*1-(2,2,5,5-Tetramethyl-1-oxyl-3-pyrrolidinyl)-3,3-(3-oxapentyleno) selenourea* (**4d**). 
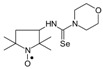
 C_13_H_24_N_3_O_2_Se, M = 334, 0.348 g, Yield: 80.2%, yellow solid, m.p. 135–139 °C (dec.), TLC: *R*_f_ = 0.16 benzene:methanol 9:1; purity (HPLC, method b): 97.2%; MS (EI, 70 eV, *m*/*z*, int [%]): 335 (9), 334 (12, M), 332 (7), 304 (18), 302 (19), 289 (10), 261 (80), 259 (40), 248 (24), 235 (20), 233 (28), 231 (13), 221 (36), 219 (21), 178 (100), 176 (52), 167 (63), 134 (32), 132 (16), 126 (18), 124 (45), 113 (52), 111 (16), 100 (54), 98 (49), 95 (37), 86 (58), 69 (52), 56 (54), 55 (43), 42 (34), 41 (53), MS (ESI, *m*/*z*, int [%]): 357 (100, M + Na), 355 (20); HR MS (ESI, *m*/*z*) for C_13_H_24_N_3_O_2_SeNa: calcd: 357.0931, found: 357.0928; IR (ν, cm^−1^, KBr): 3358, 2973, 1543, 1339, 1231, 1220, 1121, 1025, 878.

*1-(2,2,5,5-Tetramethyl-1-oxyl-3-pyrrolidinyl)-3-cyclododecyl selenourea* (**4e**). 
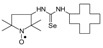
 C_21_H_40_N_3_OSe, M = 430, 0.376 g, Yield: 87.2%, beige crystalline powder, m.p. 142–150 °C (dec.), TLC: *R*_f_ = 0.16 benzene:methanol 9:1; purity (HPLC, method c): 99.4%; MS (EI, 70 eV, *m*/*z*, int [%]): 431 (42), 430 (19, M), 429 (22), 414 (18), 412 (29), 410 (14), 398 (11), 357 (70), 355 (37), 344 (21), 318 (14), 317 (12), 315 (12), 291 (14), 289 (11). 277 (10), 275 (14), 263 (19), 182 (36), 168 (22), 151 (16), 149 (12), 126 (52), 124 (38), 112 (12), 111 (38), 110 (39), 99 (62), 98 (89), 84 (100), 69 (50), 67 (25), 56 (56), 55 (64), 43 (29), 41 (46); MS (ESI, *m*/*z*, int [%]): 453 (100, M + 23), 451 (20); HR MS (ESI, *m*/*z*) for C_21_H_40_N_3_OSeNa: calcd: 453.2234, found: 453.2254; IR (ν, cm^−1^, KBr): 3434, 2932, 1552, 1469, 1364.

*1-(2,2,5,5-Tetramethyl-1-oxyl-3-pyrrolidinyl)-3-(1-adamantyl) selenourea* (**4f**). 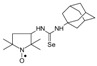 C_19_H_32_N_3_OSe, M = 398, 0.260 g, Yield: 72.8%, pale beige crystalline powder m.p. 140–145 °C, TLC: *R*_f_ = 0.21 benzene:methanol 9:1; purity (HPLC, method a): 99.5%; MS (EI, 70 eV, *m*/*z*, int [%]): 399 (3, M + 1), 398 (1, M), 397 (1), 396 (1), 135 (100), 110 (6), 107 (10), 99 (37), 98 (11), 94 (12), 93 (17), 84 (20), 79 (18), 71 (8), 67 (11), 56 (14), 55 (8), 41 (16); MS (ESI, *m*/*z*, int [%]): 421 (100, M + 23), 419 (20); HR MS (ESI, *m*/*z*) for C_19_H_32_N_3_OSeNa: calcd: 421.1608, found: 421.1592; IR (ν, cm^−1^, KBr): 3439, 2910, 1544.

*1-(2,2,5,5-Tetramethyl-1-oxyl-3-pyrrolidinyl)-3-phenyl selenourea* (**4g**). 
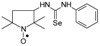
 C_15_H_22_N_3_OSe, M = 340, 0.149 g, Yield: 29.2%, beige crystalline powder, m.p. 135–136 °C, TLC: *R*_f_ = 0.15 benzene:methanol 9:1; purity (HPLC, method a): 94.1%; MS (EI, 70 eV, *m*/*z*, int [%]): 341 (16), 340 (12, M), 339 (8), 338 (9), 322 (8), 310 (8), 308 (9), 295 (8), 267 (42), 265 (23), 254 (17), 244 (13), 243 (20), 239 (14), 229 (15), 228 (28), 227 (18), 201 (11), 199 (16), 188 (16), 187 (19), 186 (14), 185 (25), 184 (15), 173 (42), 171 (24), 159 (21), 158 (16), 157 (12), 156 (10), 145 (15), 144 (21), 126 (36), 124 (28), 119 (60), 118 (29), 111 (22), 110 (54), 108 (11), 104 (31), 99 (90), 98 (55), 96 (14), 95 (32), 94 (18), 93 (37), 92 (18), 91 (12), 84 (100), 82 (12), 81 (11), 78 (19), 77 (67), 71 (24), 70 (32), 69 (37), 68 (10), 67 (22), 65 (20), 58 (27), 57 (18), 56 (77), 55 (33), 53 (14), 51 (19), 43 (17), 42 (34), 41 (54); MS (ESI, *m*/*z*, int [%]): 363 (100, M + Na), 361 (15), 301 (20), 271 (5); HR MS (ESI, *m*/*z*) for C_15_H_22_N_3_OSeNa: calcd: 363.0826, found: 363.0831; IR (ν, cm^−1^, KBr): 3273, 2982, 1547, 1240, 692.

*1,3-Bis(2,2,5,5-tetramethyl-1-oxyl-3-pyrrolidinyl) selenourea* (**4h**). 
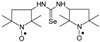
 C_17_H_32_N_4_O_2_Se, M = 404, 0.343 g, Yield: 85.8%, m.p. 68–73 °C, TLC: *R*_f_ = 0.21 benzene:methanol 9:1, yellow, glassy solid; purity (HPLC, method a): 97.8%; MS (EI, 70 eV, *m*/*z*, int [%]): 406 (6), 405 (4), 404 (15, M), 402 (8), 386 (19), 374 (10), 372 (9), 340 (4), 322 (9), 250 (12), 245 (14), 177 (10), 142 (24), 126 (70), 124 (35), 111 (40), 110 (58), 98 (100), 95 (30), 84 (84), 70 (24), 69 (42), 67 (20), 58 (36), 56 (93), 55 (37), 43 (24), 42 (19), 41 (38); MS (ESI, *m*/*z*, int [%]): 427 (100, M + Na), 425 (10); HR MS (ESI, *m*/*z*) for C_17_H_32_N_4_O_2_SeNa: calcd: 427.1588, found: 427.1591; IR (ν, cm^−1^, KBr): 3437, 2973, 1547, 1462, 1365.

*1-[(2,2,5,5-Tetramethyl-1-oxyl-3-pyrrolidinyl)methyl]-3-methyl selenourea* (**5a**). 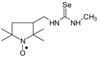 C_11_H_22_N_3_OSe, M = 292, 0.31 g, Yield: 76.0%, yellow crystalline powder, m.p. 117–122 °C (dec.), TLC: *R*_f_ = 0.5 benzene:acetone 1:1; purity (HPLC, method b): 97.3%; MS (EI, 70 eV, *m*/*z*, int [%]): 293 (16), 292 (31, M), 290 (16), 262 (13), 260 (11), 219 (13), 217 (7), 210 (7), 206 (9), 196 (13), 195 (10), 180 (95), 163 (19), 151 (28), 140 (51), 124 (70), 122 (18), 112 (16), 111 (13), 110 (20), 109 (25), 98 (27), 84 (12), 83 (17), 82 (20), 81 (17), 78 (55), 69 (100), 58 (27), 57 (34), 56 (22), 55 (32), 42 (32), 41 (51); MS (ESI, *m*/*z*, int [%]): 315 (100, M + Na), 313 (10); HR MS (ESI, *m*/*z*) for C_11_H_22_N_3_OSeNa: calcd: 315.0826, found: 315.0832; IR (ν, cm^−1^, KBr) 3434, 2971, 2930, 1567, 1460, 1364, 681.

*1-[(2,2,5,5-Tetramethyl-1-oxyl-3-pyrrolidinyl)methyl]-3,3-dimethyl selenourea* (**5b**). 
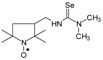
 C_12_H_24_N_3_OSe, M = 306, 0.3 g, Yield: 78.9%, yellow crystalline powder, m.p. 162–170 °C, TLC: *R*_f_ = 0.31 benzene:acetone 1:1; purity (HPLC, method b): 99.1%; MS (EI, 70 eV, *m*/*z*, int [%]): 306 (84, M), 304 (41), 276 (44), 274 (37), 272 (16), 233 (75), 231 (39), 220 (26), 195 (16), 177 (82), 175 (41), 153 (19), 152 (15), 140 (24), 138 (27), 136 (100), 134 (48), 124 (24), 121 (14), 109 (26), 98 (17), 85 (15), 81 (14), 71 (85), 69 (38), 56 (19), 55 (25), 44 (29), 42 (24), 41 (50); MS (ESI, *m*/*z*, int [%]): 329 (100, M + Na), 327 (20); HR MS (ESI, *m*/*z*) for C_12_H_24_N_3_OSeNa: calcd: 329.0982, found: 329.0976; IR (ν, cm^−1^, KBr): 3440, 3293, 2969, 2929, 1552, 1459, 1373.

*1-[(2,2,5,5-Tetramethyl-1-oxyl-3-pyrrolidinyl)methyl]-3,3-pentyleno selenourea* (**5c**). 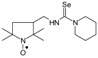 C_15_H_28_N_3_OSe, M = 346, 0.319 g, Yield: 89.6%, yellow crystalline powder, m.p. 165–171 °C, TLC: *R*_f_ = 0.17 benzene:methanol 9:1; purity (HPLC, method a): 97.7%; MS (EI, 70 eV, *m*/*z*, int [%]): 346 (76, M), 344 (39), 316 (31), 314 (31), 312 (14), 273 (100), 271 (52), 260 (23), 259 (20), 235 (21), 217 (91), 215 (46), 193 (14), 176 (71), 174 (35), 166 (8), 152 (8), 140 (15), 124 (15), 111 (76), 84 (63), 69 (71), 56 (28), 55 (38), 41 (67); MS (ESI, *m*/*z*, int [%]): 369 (100, M + Na), 367 (15); HR MS (ESI, *m*/*z*) for C_15_H_28_N_3_OSeNa: calcd: 369.1295, found: 369.1290; IR (ν, cm^−1^, KBr): 3352, 2926, 1553, 1332.

*1-[(2,2,5,5-Tetramethyl-1-oxyl-3-pyrrolidinyl)methyl]-3,3-(3-oxapentyleno) selenourea* (**5d**). 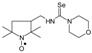 C_14_H_26_N_3_O_2_Se, M = 348, 0.315 g, Yield: 88.0%, m.p. 140–146 °C, TLC: *R*_f_ = 0.38 benzene:acetone 1:1, yellow solid; purity (HPLC, method b): 99.4%; MS (EI, 70 eV, *m*/*z*, int [%]): 348 (88, M), 346 (46), 318 (44), 316 (40), 314 (18), 275 (100), 273 (51), 262 (37), 261 (38), 260 (19), 259 (24), 237 (21), 219 (97), 217 (50), 167 (9), 152 (11), 140 (22), 134 (25), 124 (28), 113 (66), 109 (32), 98 (18), 86 (38), 83 (17), 81 (21), 69 (96), 67 (22), 57 (23), 56 (36), 55 (36), 41 (70); MS (ESI, *m*/*z*, int [%]): 371 (100, M + Na), 369 (25), 193 (20); HR MS (ESI, *m*/*z*) for C_14_H_26_N_3_O_2_SeNa: calcd: 371.1088, found: 371.1097; IR (ν, cm^−1^, KBr): 3364, 2970, 1547, 1462, 1420, 1363, 1334, 1273, 1250, 1212, 1119, 1022.

*1-[(2,2,5,5-Tetramethyl-1-oxyl-3-pyrrolidinyl)methyl]-3-cyclododecyl selenourea* (**5e**). 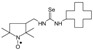 C_22_H_42_N_3_OSe, M = 444, 0.276 g, Yield: 82.9%, m.p. 145–150 °C, TLC: *R*_f_ = 0.16 benzene:methanol 9:1; yellow solid; purity (HPLC, method c): 97.6%; MS (EI, 70 eV, *m*/*z*, int [%]): 445 (20), 444 (21, M), 443 (10), 442 (12), 429 (3), 414 (7), 412 (9), 371 (34), 369 (18), 362 (18), 358 (19), 347 (15), 332 (100), 315 (23), 313 (12), 304 (7), 291 (14), 289 (9), 276 (14), 264 (7), 250 (5), 247 (5), 234 (4), 221 (9), 197 (11), 182 (61), 166 (12), 151 (8), 140 (49), 138 (16), 124 (34), 111 (23), 110 (19), 97 (25), 83 (33), 81 (23), 69 (51), 67 (22), 55 (61), 43 (24), 41 (51); MS (ESI, *m*/*z*, int [%]): 467 (100, M + Na), 465 (20); HR MS (ESI, *m*/*z*) for C_22_H_42_N_3_OSeNa: calcd: 467.2391, found: 467.2386; IR (ν, cm^−1^, KBr): 3308, 2931, 1550.

*1-[(2,2,5,5-Tetramethyl-1-oxyl-3-pyrrolidinyl)methyl]-3-(1-adamantyl) selenourea* (**5f**). 
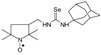
 C_20_H_34_N_3_OSe, M = 412, 0.335 g, Yield: 58.1%, m.p. 78–82 °C, TLC: *R*_f_ = 0.26 benzene:methanol 9:1, yellow crystals; purity (HPLC, method a): 98.4%; MS (EI, 70 eV, *m*/*z*, int [%]): 413 (2), 412 (2, M), 330 (4), 300 (23), 135 (100), 124 (8), 94 (7), 93 (12), 79 (12), 55 (7), 41 (8); MS (ESI, *m*/*z*, int [%]): 435 (100, M + Na), 433 (10); HR MS (ESI, *m*/*z*) for C_20_H_34_N_3_OSeNa: calcd: 435.1765, found: 435.1767; IR (ν, cm^−1^, KBr): 3430, 2907, 1543, 683.

*1-[(2,2,5,5-Tetramethyl-1-oxyl-3-pyrrolidinyl)methyl]-3-phenyl selenourea* (**5g**). 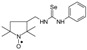 C_16_H_24_N_3_OSe, M = 354, 0.170 g, Yield: 46.7%, beige crystalline powder, m.p. 130–135 °C, TLC: *R*_f_ = 0.25 benzene:methanol 9:1; purity (HPLC, method a): 95.9%; MS (EI, 70 eV, *m*/*z*, int [%]): 355 (34), 354 (55, M), 352 (25), 339 (10), 337 (7), 324 (25), 322 (22), 320 (11), 281 (40), 279 (23), 272 (20), 268 (25), 260 (46), 258 (40), 257 (66), 243 (44), 242 (75), 226 (51), 225 (53), 223 (24), 213 (16), 204 (33), 203 (37), 201 (21), 199 (17), 184 (24), 183 (22), 174 (16), 173 (15), 167 (13), 157 (21), 140 (75), 133 (27), 131 (98), 124 (100), 119 (93), 118 (48), 110 (20), 109 (24), 99 (19), 98 (25), 93 (99), 81 (25), 77 (83), 69 (59), 67 (24), 65 (25), 58 (26), 56 (23), 55 (39), 51 (23), 41 (76); MS (ESI, *m*/*z*, int [%]): 377 (100, M + Na), 375 (30), 193 (20), 133 (10); HR MS (ESI, *m*/*z*) for C_16_H_24_N_3_OSeNa: calcd 377.0982, found: 377.0970; IR (ν, cm^−1^, KBr): 3161, 2967, 1552.

*1,3-bis[(2,2,5,5-tetramethyl-1-oxyl-3-pyrrolidinyl)methyl] selenourea* (**5h**). 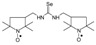 C_19_H_36_N_4_O_2_Se, M = 432, 0.249 g, Yield: 75.5%, m.p. 50–58 °C, TLC: *R*_f_ = 0.38 benzene:acetone 1:1, yellow glass; MS (EI, 70 eV, *m*/*z*, int [%]): 433 (4), 432 (4, M), 403 (3), 368 (5), 354 (11), 352 (15), 351 (10), 338 (26), 336 (31), 323 (10), 321 (14), 320 (12), 305 (24), 262 (32), 252 (13), 249 (15), 248 (13), 222 (14), 220 (13), 210 (12), 199 (23), 198 (25), 194 (29), 183 (46), 182 (54), 166 (21), 140 (69), 138 (40), 124 (100), 111 (20), 110 (24), 99 (22), 98 (22), 96 (20), 95 (16), 84 (22), 81 (20), 69 (30), 67 (19), 58 (26), 55 (31), 41 (38); MS (ESI, *m*/*z*, int [%]): 455 (100, M+Na), 453 (20); HR MS (ESI, *m*/*z*) for C_19_H_36_N_4_O_2_SeNa: calcd 455.1901, found: 455.1914; IR (ν, cm^−1^, KBr): 3432, 2971, 1557, 1462, 1364.

*1-(2,2,6,6-Tetramethyl-1-oxyl-4-piperidynylyl)-3-methyl selenourea* (**6a**). 
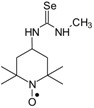
 C_11_H_22_N_3_OSe, M = 292, 0.28 g, Yield: 64.1%, beige crystalline powder, m.p. 140 °C (dec.), TLC: *R*_f_ = 0.14 benzene:methanol 9:1; purity (HPLC, method b): 95.7%; MS (EI, 70 eV, *m*/*z*, int [%]): 294 (21), 293 (33), 292 (100, M), 290 (52), 289 (21), 288 (18), 260 (12), 258 (7), 219 (52), 217 (27), 180 (41), 163 (32), 140 (56), 139 (40), 124 (87), 109 (62), 98 (38), 97 (18), 96 (17), 84 (22), 83 (20), 82 (20), 81 (15), 69 (47), 67 (17), 58 (22), 57 (41), 56 (19), 55 (29), 42 (26), 41 (35); MS (ESI, *m*/*z*, int [%]): 315 (100, M + Na), 313 (25), 242 (60); HR MS (ESI, *m*/*z*) for C_11_H_22_N_3_OSeNa: calcd: 315.0826, found: 315.0823; IR (ν, cm^−1^, KBr): 3380, 3319, 1561.

*1-(2,2,6,6-Tetramethyl-1-oxyl-4-piperidynylyl)-3-(1-cyclododecyl) selenourea* (**6b**). 
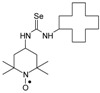
 C_22_H_42_N_3_OSe, M = 444, 0.6 g, Yield: 91.0%, beige crystalline powder, m.p. 143–146 °C (dec.), TLC: *R*_f_ = 0.19 benzene:methanol 9:1; purity (HPLC, method c): 98.9%; MS (EI, 70 eV, *m*/*z*, int [%]): 447 (16), 446 (25), 445 (78, M+1), 444 (45, M), 443 (41), 442 (33), 441 (20), 429 (7), 415 (7), 412 (8), 371 (88), 369 (47), 332 (42), 315 (22), 313 (13), 291 (31), 289 (19), 182 (43), 157 (24), 155 (30), 140 (100), 124 (54), 109 (16), 98 (36), 83 (25), 74 (19), 69 (37), 55 (50), 43 (19), 41 (33); MS (ESI, *m*/*z*, int [%]): 467 (100, M + 23), 465 (20); HR MS (ESI, *m*/*z*) for C_22_H_42_N_3_OSeNa: calcd: 467.2391, found: 467.2383; IR (ν, cm^−1^, KBr): 3434, 3320, 2932, 1542.

*1-(2,2,5,5-Tetramethyl-1-oxyl-3-pyrrolidinyl)-3-(2,2,6,6-tetramethyl-1-oxyl-4-piperidynylyl) selenourea* (**7**). 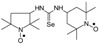 C_18_H_34_N_4_O_2_Se M = 417, 0.27 g; Yield: 93.1%, oil, TLC: *R*_f_ = 0.52 benzene:acetone 1:1, red oil; slow decomposition with evolving of selenium; purity (HPLC, method a): 99.8%; MS (EI, 70 eV, *m*/*z*, int [%]): 420 (13), 419 (16), 418 (43, M), 416 (22), 388 (4), 387 (6), 386 (7), 336 (24), 322 (11), 306 (17), 266 (11), 264 (20), 263 (11), 259 (16), 247 (16), 233 (13), 208 (22), 162 (12), 160 (32), 158 (27), 157 (11), 156 (19), 155 (16), 154 (20), 140 (89), 126 (49), 124 (100), 111 (43), 110 (46), 109 (50), 108 (19), 99 (44), 98 (91), 84 (86), 74 (47), 73 (28), 71 (22), 70 (46), 69 (68), 68 (17), 67 (29), 58 (58), 56 (99), 55 (67), 43 (73), 42 (34), 41 (67); MS (ESI, *m*/*z*, int [%]): 441(100, M + 23), 439 (10); HR MS (ESI, *m*/*z*) for C_18_H_34_N_4_O_2_SeNa: calcd: 441.1745, found: 441.1759; IR (ν, cm^−1^, film): 3316, 2973, 1545, 1461, 1364, 1330, 1242, 1180, 682.

*1-(1-Adamantyl)-3-(2,2,6,6-tetramethyl-1-oxyl-4-piperidynylyl) selenourea* (**8a**). 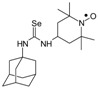 C_20_H_34_N_3_Ose, M = 412, 0.436 g, Yield: 36.0%, orange crystalline powder, m.p. 151–156 °C, TLC: *R*_f_ = 0.37 benzene:methanol 9:1; purity (HPLC, method a): 99.6%; MS (EI, 70 eV, *m*/*z*, int [%]): 318 (9), 300 (20), 177 (5), 160 (6), 151 (6), 140 (12), 135 (41), 124 (100), 98 (17), 94 (22), 79 (10), 67 (7), 58 (9), 57 (7), 56 (4), 42 (12), 41 (13); MS (ESI, *m*/*z*, int [%]): 435 (100, M + Na); HR MS (ESI): for C_20_H_34_N_3_OSeNa: calcd: 435.1765, found: 435.1746; IR (ν, cm^−1^, KBr): 3433, 2913, 1539.

*1-(3-Methylphenyl)-3-(2,2,6,6-tetramethyl-1-oxyl-4-piperidynylyl) selenourea* (**8b**). 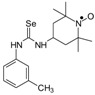 C_17_H_26_N_3_Ose, M = 368, 1.0334 g, Yield: 59.7%, brown crystalline powder, m.p. 140 °C, TLC: *R*_f_ = 0.20 benzene:methanol 9:1, purity (HPLC, method a): 95.6%; MS (EI, 70 eV, *m*/*z*, int [%]): 369 (0.5), 368 (0.5, M), 353 (1), 351 (0.5), 304 (1), 289 (3), 287 (3), 274 (15), 271 (16), 270 (8), 256 (37), 215 (10), 214 (12), 200 (19), 183 (7), 173 (5), 172 (5), 162 (12), 161 (8), 140 (18), 138 (6), 124 (100), 107 (18), 98 (33), 91 (18), 78 (98), 58 (23); MS (ESI, *m*/*z*, int [%]): 759 (5, 2*M + Na), 757 (5), 449 (7), 447 (7), 391 (85, M + Na), 389 (43), 370 (30), 369 (30, M + H), 368 (28), 367 (22), 366 (15), 365 (8), 354 (100), 352 (55), 327 (42), 290 (44); HR MS (ESI, *m*/*z*): for M + Na C_17_H_26_N_3_OSeNa, calcd.: 391.11333, found: 391.11179; IR (ν, cm^−1^, KBr): 3285, 3160, 2981, 1549.

### 3.4. Nitroxyl Selenocarbamates ***9a***–***9d*;** Reaction of the Nitroxyl Isoselenocyanates ***1a***, ***1b*** with either Sodium Methoxide or Sodium Ethoxide; a General Procedure

A sodium methoxide solution was prepared by dissolving metallic sodium (0.050 g, 0.00217 mol) in either methanol or ethanol (5 mL). The sodium methoxide (ethoxide) solution (3.5 mL, 0.0015 mol), respectively, was added dropwise to the solution of the isoselenocyanate **1a** or **1b** (0.001 mol) in methanol or ethanol (5 mL). The reagents were stirred for 1 h at room temperature. The solvent was evaporated under reduced pressure. The residue was purified by column chromatography using benzene:methanol 9:1 or benzene:ethanol 9:1, respectively, as a mobile phase. A chromatographically purified product was triturated with hexane. A crystalline precipitate was filtered off to give the appropriate nitroxyl selenocarbamate **9a**–**9d** (Scheme 3).

*Methyl N-(2,2,5,5-tetramethyl-1-oxyl-3-pyrrolidinyl) selenonocarbamate* (**9a**). 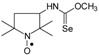 C_10_H_19_N_2_O_2_Se, M = 279, 0.37 g, Yield: 94.9%, yellow crystalline powder, m.p. 133–136 °C, TLC: *R*_f_ = 0.33 benzene:methanol 9:1; purity (HPLC, method b): 99.6%; MS (EI, 70 eV, *m*/*z*, int [%]): 280 (28), 279 (60, M), 277 (35), 249 (21), 247 (29), 245 (15), 234 (7), 222 (12), 220 (7), 206 (95), 204 (40), 203 (16), 202 (18), 193 (35), 191 (19), 178 (38), 176 (22), 168 (17), 166 (100), 164 (51), 163 (18), 162 (19), 149 (40), 147 (21), 136 (15), 126 (32), 125 (20), 124 (16), 123 (30), 112 (43), 111 (17), 110 (66), 100 (87), 98 (35), 95 (33), 82 (10), 81 (10), 69 (29), 67 (25), 58 (58), 56 (56), 55 (24), 42 (25), 41 (33); HR MS (EI, 70 eV, *m*/*z*) for C_10_H_19_N_2_O_2_Se: calcd: 279.06117, found: 279.06205; IR (ν, cm^−1^, KBr): 3420, 3235, 2973, 1537, 1458, 1386, 1208, 1138, 997.

*Ethyl N-(2,2,5,5-tetramethyl-1-oxyl-3-pyrrolidinyl) selenonocarbamate* (**9b**). 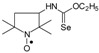 C_11_H_21_N_2_O_2_Se, M = 293, 0.365 g, Yield: 83.0%, yellow crystalline powder, m.p. 120–125 °C, TLC: *R*_f_ = 0.38 benzene:methanol 9:1; purity (HPLC, method b): 99.4%; MS (EI, 70 eV, *m*/*z*, int [%]): 293 (13, M), 291 (7), 263 (7), 261 (9), 236 (5), 234 (7), 220 (22), 218 (11), 207 (13), 205 (7), 192 (22), 190 (11), 182 (11), 180 (47), 178 (35), 176 (16), 154 (10), 152 (23), 150 (17), 149 (19), 126 (32), 125 (16), 124 (18), 110 (64), 100 (50), 98 (68), 95 (47), 84 (41), 70 (32), 69 (31), 67 (23), 58 (42), 57 (22), 56 (100), 55 (35), 43 (22), 42 (18), 41 (39); MS (ESI, *m*/*z*, int [%]): 316 (100, M + Na), 314 (15); HR MS (ESI, *m*/*z*) for C_11_H_21_N_2_O_2_SeNa: calcd: 316.0666, found: 316.0656; IR (ν, cm^−1^, KBr): 3420, 3225, 1541, 1463, 1379, 1208, 1020.

*Methyl N-((2,2,5,5-tetramethyl-1-oxyl-3-pyrrolidinyl)methyl) selenonocarbamate* (**9c**). 
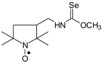
 C_11_H_21_N_2_O_2_Se, M = 293, 0.246 g, Yield: 84.0%, beige crystalline powder, m.p. 140–145 °C, TLC: *R*_f_ = 0.31 benzene:methanol 9:1; purity (HPLC, method b): 99.2%; MS (EI, 70 eV, *m*/*z*, int [%]): 295 (18), 294 (15), 293 (96, M), 291 (48), 290 (17), 289 (18), 263 (73), 261 (59), 259 (25), 220 (49), 218 (24), 207 (32), 205 (16), 182 (35), 164 (55), 162 (27), 152 (44), 150 (25), 149 (25), 140 (44), 139 (49), 126 (29), 124 (39), 123 (49), 113 (28), 109 (69), 95 (19), 81 (38), 74 (18), 69 (100), 67 (33), 58 (26), 56 (28), 55 (42), 53 (21), 44 (62), 41 (96); MS (ESI, *m*/*z*, int [%]): 316 (100, M + Na), 314 (25), 304 (35), 227 (20); HR MS (ESI, *m*/*z*) for C_11_H_21_N_2_O_2_SeNa: calcd: 316.0666, found: 316.0672; IR (ν, cm^−1^, KBr): 3200, 2972, 1552, 1205, 973, 566.

*Ethyl N-((2,2,5,5-tetramethyl-1-oxyl-3-pyrrolidinyl)methyl) selenonocarbamate* (**9d**). 
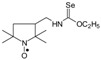
 C_12_H_23_N_2_O_2_Se, M = 307, 0.225 g, Yield: 79.8%, beige crystalline powder, m.p. 120–130 °C, TLC: *R*_f_ = 0.31 benzene:methanol 9:1; purity (HPLC, method b): 99.5%; MS (EI, 70 eV, *m*/*z*, int [%]): 309 (19), 308 (17), 307 (100, M), 305 (52), 304 (19), 303 (20), 277 (65), 275 (49), 261 (5), 247 (10), 234 (42), 232 (20), 221 (32), 219 (16), 206 (12), 196 (28), 192 (26), 190 (13), 183 (22), 178 (51), 176 (25), 166 (20), 164 (13), 154 (30), 149 (21), 140 (35), 139 (33), 138 (47), 127 (22), 126 (30), 124 (40), 123 (22), 109 (64), 98 (13), 96 (11), 95 (15), 84 (23), 83 (17), 82 (21), 81 (36), 74 (19), 69 (94), 67 (30), 58 (36), 56 (32), 55 (47), 53 (19), 41 (92); MS (ESI, *m*/*z*, int [%]): 330 (100, M + Na), 328 (25); HR MS (ESI, *m*/*z*) for C_12_H_23_N_2_O_2_SeNa: calcd 330.0822, found: 330.0808; IR (ν, cm^−1^, KBr): 3200, 2971, 1556, 1414, 1201, 1019.

### 3.5. Nitroxyl Selenocarbamates ***10a***–***10d***; Reaction of the Isoselenocyanates ***1d***–***1g*** with 4-hydroxy-2,2,6,6-tetramethyl-piperidine-1-oxyl (***3c***); a General Procedure

4-Hydroxy-2,2,6,6-tetramethyl-piperidine-1-oxyl (**3c**, 0.69 g, 0.004 mola) was dissolved in anhydrous THF (15 mL). Sodium hydride as 60% dispersion in mineral oil (0.2 g, 0.005 mol) was added. The reagents were refluxed for 1 h. The reaction mixture was allowed to cool to room temperature. Isoselenocyanate **1d**–**1g** (0.004 mola) was added. The reagents were stirred for 1 h at room temperature. Methylene chloride (15 mL) and water (15 mL) were added to the reaction mixture. The layers were separated. The organic layer was dried over anhydrous magnesium sulfate and concentrated to a constant mass. To the residue either the mixture of benzene (1 mL) and hexane (1 mL) or pure hexane was added. The mixture was stirred for 5 min. The precipitate was filtered off to give nitroxyl selenocarbamate **10a**–**10d** (Scheme 4).

*2,2,6,6-Tetramethyl-1-oxyl-4-piperidinyl N-(1-adamantyl) selenonocarbamate* (**10a**). 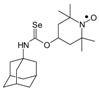 C_20_H_33_N_2_O_2_Se, M = 413, 1.18 g, Yield: 71.6%, orange crystalline powder, m.p. 198–203 °C, TLC: *R*_f_ = 0.51 benzene:ethyl acetate 4:1; purity (HPLC, method c): 99.3%; MS (EI, 70 eV, *m*/*z*, int [%]): 413 (5, M), 411 (3), 260 (8), 258 (4), 177 (16), 154 (53), 140 (15), 135 (100), 124 (98), 120 (40), 109 (45), 94 (37), 93 (17), 83 (8), 82 (10), 81 (16), 79 (19), 77 (10), 74 (18), 71 (6), 69 (14), 67 (20), 55 (28), 41 (33); HR MS (EI, 70 eV, *m*/*z*) for C_20_H_33_N_2_O_2_Se: calcd: 413.17072, found: 413.17190; IR (ν, cm^−1^, KBr): 3434, 3200, 2913, 1534, 1198, 1156.

*2,2,6,6-Tetramethyl-1-oxyl-4-piperidinyl N-(3-methylphenyl) selenonocarbamate* (**10b**). 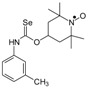 C_17_H_25_N_2_O_2_Se, M = 369, 0.4606 g, Yield: 36.1%, m.p. 48–51 °C, TLC: *R*_f_ = 0.45 benzene:ethyl acetate 9:1 *R*_f_ = 0.65 benzene:methanol 9:1, red glass; MS (EI, 70 eV, *m*/*z*, int [%]): 370 (4), 369 (6, M), 368 (2), 367 (3), 366 (2), 248 (1), 243 (2), 216 (3), 215 (2), 214 (3), 197 (19), 195 (9), 194 (4), 156 (18), 155 (29), 154 (75), 140 (39), 133 (22), 134 (25), 124 (100), 109 (83), 107 (20), 106 (18), 100 (13), 98 (11), 91 (27), 74 (20), 55 (25), 41 (24). HR MS (EI, 70 eV, *m*/*z*): for C_17_H_25_N_2_O_2_Se, calcd.: 369.10812, found: 369.10737, IR (ν, cm^−1^, film): 1634, 1531, 1364, 1179, 1140. 

*2,2,6,6-Tetramethyl-1-oxyl-4-piperidinyl N-(4-(trifluoromethyl)phenyl) selenonocarbamate* (**10c**). 
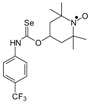
 C_17_H_22_F_3_N_2_O_2_Se, M = 423, 0.670 g, Yield: 63.5%, beige crystalline powder, m.p. 119–123 °C (dec.), TLC: *R*_f_ = 0.59 hexane:ethyl acetate 9:1; purity (HPLC, method a): 99.6%; MS (EI, 70 eV, *m*/*z*, int [%]): 424 (5), 423 (4, M), 422 (3), 421 (3), 251 (12), 249 (6), 188 (12), 187 (20), 156 (37), 155 (65), 154 (42), 145 (27), 140 (45), 124 (71), 109 (54), 100 (48), 98 (22), 83 (32), 82 (20), 81 (23), 74 (100), 70 (6), 68 (10), 56 (42), 55 (75), 41 (59); HR MS (EI, 70 eV, *m*/*z*): for C_17_H_22_N_2_O_2_F_3_Se, calcd.: 423.07986, found: 423.08047; IR (ν, cm^−1^, KBr): 3436, 1618, 1525, 1324, 1166, 1120, 1068, 840.

*2,2,6,6-Tetramethyl-1-oxyl-4-piperidinyl N-(4-chloro-2-methylphenyl) selenonocarbamate* (**10d**). 
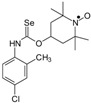
 C_17_H_24_ClN_2_O_2_Se, M = 403, 0.595 g, Yield: 36.9%, yellow crystalline powder, m.p. 115–122 °C, TLC: *R*_f_ = 0.49 benzene:ethyl acetate 4:1; MS (EI, 70 eV, *m*/*z*, int [%]): 406 (5), 405 (7), 404 (10), 403 (16, M), 402 (6), 401 (9), 250 (5), 248 (3), 233 (13), 231 (24), 229 (10), 196 (4), 169 (14), 168 (14), 167 (47), 155 (43), 154 (60), 141 (35), 140 (44), 132 (22), 124 (100), 116 (17), 109 (75), 106 (18), 100 (21), 98 (27), 89 (18), 83 (18), 82 (3), 81 (20), 78 (26), 77 (25), 74 (46), 71 (25), 69 (34), 67 (37), 57 (50), 56 (52), 55 (59), 53 (10), 52 (12), 51 (16), 50 (13), 43 (43), 42 (33), 41 (92), 39 (35); MS (ESI, *m*/*z*, int [%]): 827 (10), 428 (20), 426 (100, M + 23), 424 (30); HR MS (ESI, 70 eV, *m*/*z*) for C_17_H_24_ClN_2_O_2_NaSe: calcd: 426.0589, found: 426.0574; IR (ν, cm^−1^, KBr): 3434, 1634, 1510, 1363, 1200, 1149.

### 3.6. Antifungal Activity Assays

Fungitoxicity of the tested compounds against phytopathogenic fungi was assessed in vitro using agar growth medium poison technique. PDA media in 100 mm Petri plates containing the acetone solutions of the tested compounds in the defined concentrations were infected with agar disks with thin mycelium of fungi cultures and allowed the solvent to evaporate. Linear growth of each colony was determined after 3–5 days. The effect of each compound on mycelial growth was assessed by calculating the percentage of growth reduction, where: percentage of linear growth reduction = [(colony diameter of the control plate – colony diameter of the tested plate)/(colony diameter of the control plate)] × 100.

### 3.7. Antibacterial Activity Assays

Antibacterial tests were performed by dilution method on a solid support. The test results were read after 48 h incubation of the plates at 25 °C with bacterial strains. The antibacterial activity of the compounds was expressed in terms of the minimum growth inhibitory concentration of the test strain (MIC) in mg/L. Plant pathogenic strains: *Erwinia carotovora*, *Pseudomonas phaseolicola Pseudomonas lachrymans*, *Pseudomonas syringae*, were used. 

## 4. Conclusions

Selenourea and selenocarbamate nitroxides **4**–**10** were synthesized in the reaction of corresponding isoselenocyanates and either amines or alcohols, respectively. The investigated selenium compounds **4**–**10** were tested in vitro against nine pathogenic fungi, and against four phytopathogenic bacteria. Significant fungistatic and bacteriostatic activities of the investigated compounds were found. Ten nitroxide selenoureas **4c**, **4d**, **4h**, **5a**–**5d**, **5g**, **5h**, and **7** were shown the fungistatic activity at the concentration of 20 mg/L at 100% level (MIC ≤ 20). Twelve nitroxide selenoureas **4a**, **4c**, **4d**, **4f**, **4g**, **5b**, **5c**, **5g**, **5h**, **6a**, **6b**, and **8a**, and two nitroxide selenocarbamates **9b**, and **10d**, have shown bacteriostatic activity at the concentration of 100 mg/L (MIC ≤ 100). 

## Figures and Tables

**Table 1:** Fungistatic activities ^a^ of selenoureas **4**–**8** at 200 mg/L and 20 mg/L.

Cmpd	*B. cinerea*	*F. culmorum*	*P. cactorum*	*R. solani*	*B. graminis*	*A. alternata*	*F. oxysporum*	*P. infestans*	*A. apis*
	In Vitro	In Vitro	In Vitro	In Vitro	In Vivo	In Vitro	In Vitro	In Vitro	In Vitro
	200 mg/L/20 mg/L	200 mg/L/20 mg/L	200 mg/L/20 mg/L	200 mg/L/20 mg/L	1000 mg/L	200 mg/L/20 mg/L	200 mg/L/20 mg/L	200 mg/L/20 mg/L	200 mg/L/20 mg/L
**4a**	100/68	100/28	78/50	100/40	24.6	−/26	−/76	−/22	−/68
**4b**	100/73	100/19	100/42	100/66	24.6	−/30	−/71	−/20	−/−
**4c**	100/100	54/21	100/100	100/44	26.7	−/33	−/69	−/26	−/72
**4d**	100/74	100/21	100/100	100/36	25	−/23	−/69	−/20	−/48
**4e**	65/−	24/−	67/−	26/−	46.5	−/−	−/−	−/−	−/−
**4f**	31/3	49/0	100/19	100/0	1.6	−/2	−/14	−/14	−/0
**4g**	91/0	18/6	100/65	100/0	11.4	−/9	−/43	−/20	−/36
**4h**	100/74	72/19	100/100	100/50	22	−/30	−/80	−/28	−/46
**5a**	100/64	3/24	100/100	100/16	31.8	−/21	−/66	−/20	−/48
**5b**	100/82	100/51	100/100	100/60	26.7	−/37	−/85	−/42	−/72
**5c**	100/77	100/56	100/100	100/50	4.8	−/42	−/85	−/46	−/60
**5d**	100/79	100/38	100/100	100/72	4	−/35	−/82	−/46	−/66
**5e**	81/−	56/−	100/−	62/−	12/−	−/−	−/−	−/−	−/−
**5f**	49/−	54/−	61/−	100/−	3/−	−/−	−/−	−/−	−/−
**5g**	100/49	51/24	100/100	100/0	8.9	−/23	−/52	−/20	−/−
**5h**	100/77	100/33	100/100	100/28	4	−/30	−/61	−/20	−/72
**6a**	100/49	100/19	48.1/42	100/0	18.4	−/19	−/56	−/32	−/32
**6b**	100/60	100/21	100/62	100/0	2.7	−/26	−/37	−/30	−/10
**7**	100/74	100/33	100/100	100/26	6.5	−/23	−/70	−/30	−/−
**8a**	62/−	34/−	39/−	46/−	0	−/−	−/−	−/−	−/−
**8b**	100/−	25/−	100/−	68/−	0	−/−	−/−	−/−	−/−
									
C ^b^	100/74	34/19	88/69	83/76	−	56/42	62/49	67/50	100/100
T ^b^	100/100	100/100	58/11	100/78	−	100/78	77/42	100/100	100/83
Th ^b^	−	−	−	−	−	−	−	−	100/100
A ^b^	−	−	−	−	100	−	−	−	−

^a^ Percentage of linear growth inhibition: [(colony diameter of a control plate – colony diameter of a tested plate)/(colony diameter of a control plate)]*100. ^b^ Reference compounds. C = Chlorothalonil, T = Tebuconazole, Th = Thiabendazole, A = Azoxystrobin.

**Table 2:** Fungistatic activities ^a^ of selenocarbamates **9**, **10** at 200 mg/L and 20 mg/L.

Cmpd	*B. cinerea*	*F. culmorum*	*P. cactorum*	*R. solani*	*B. graminis*	*A. alterneta*	*F. oxysporum*	*P. infestans*	*A. apis*
	In Vitro	In Vitro	In Vitro	In Vitro	In Vivo	In Vitro	In Vitro	In Vitro	In Vitro
	200 mg/L/20 mg/L	200 mg/L/20 mg/L	200 mg/L/20 mg/L	200 mg/L/20 mg/L	1000 mg/L	200 mg/L/20 mg/L	200 mg/L/20 mg/L	200 mg/L/20 mg/L	200 mg/L/20 mg/L
**9a**	100/62	100/10	93/27	100/0	27.1	−/56	−/55	−/28	−/50
**9b**	100/77	100/24	91/57	100/60	18.9	−/30	−/69	−/30	−/56
**9c**	100/79	100/38	100/62	100/54	3.4	−/30	−/70	−/36	−/60
**9d**	100/81	100/33	100/62	100/60	42.6	−/42	−/69	−/34	−/−
**10a**	20/−	3/−	0/−	42/−	3.1	−/−	−/−	−/−	−/−
**10b**	49/−	0/−	43/−	0/−	0	−/−	−/−	−/−	−/−
**10c**	17/−	0/−	0/−	0/−	0	−/−	−/−	−/−	−/−
**10d**	0/−	11/−	0/−	0/−	8.2	−/−	−/−	−/−	−/−
									
C ^b^	100/74	34/19	88/69	83/76	−	56/42	62/49	67/50	100/100
T ^b^	100/100	100/100	58/11	100/78	−	100/78	77/42	100/100	100/83
Th ^b^	−	−	−	−	−	−	−	−	100/100
A ^b^	−	−	−	−	100	−	−	−	−

^a^ Percentage of linear growth inhibition: [(colony diameter of a control plate – colony diameter of a tested plate)/(colony diameter of a control plate)]*100. ^b^ Reference compounds. C = Chlorothalonil, T = Tebuconazole, Th = Thiabendazole, A = Azoxystrobin.

**Table 3:** Bacteriostatic activity MIC of selenoureas **4**–**8** at 100 mg/L.

Cmpd	*E. carotovora*	*P. phaseolicola*	*P. lachrymans*	*P. syringae*
	mg/L	mg/L	mg/L	mg/L
**4a**	>100	>100	<100	<100
**4b**	>100	>100	>100	>100
**4c**	<100	<100	<100	<100
**4d**	<100	>100	>100	<100
**4e**	>100	>100	>100	>100
**4f**	>100	>100	>100	<100
**4g**	<100	>100	>100	<100
**4h**	>100	>100	>100	>100
**5a**	−	−	−	−
**5b**	<100	<100	<100	<100
**5c**	<100	<100	<100	<100
**5d**	>100	>100	>100	>100
**5e**	−	−	−	−
**5f**	>100	>100	<100	<100
**5g**	<100	>100	<100	<100
**5h**	<100	<100	<100	<100
**6a**	<100	<100	<100	<100
**6b**	<100	<100	<100	<100
**7**	−	−	−	−
**8a**	<100	>100	>100	>100
**8b**	>100	>100	>100	>100
				
CGA ^a^	1	−	1	1

^a^ 7-chloro-1-ethyl-6-fluoro-1,4-dihydro-4-oxo-3-quinolinecarboxylic acid used as a reference compound.

**Table 4:** Bacteriostatic activity MIC of selenocarbamates **9**, **10** at 100 mg/L.

Cmpd	*E. carotovora*	*P. phaseolicola*	*P. lachrymans*	*P. syringae*
	mg/L	mg/L	mg/L	mg/L
**9a**	>100	>100	>100	>100
**9b**	<100	<100	>100	<100
**9c**	>100	>100	>100	>100
**9d**	−	−	−	−
**10a**	>100	>100	>100	>100
**10b**	>100	>100	>100	>100
**10c**	>100	>100	>100	>100
**10d**	>100	>100	<100	<100
				
CGA ^a^	1	−	1	1

^a^ 7-chloro-1-ethyl-6-fluoro-1,4-dihydro-4-oxo-3-quinolinecarboxylic acid used as a reference compound.

## Supplementary Materials

The supplementary materials are available online. Mass spectrometry EI MS, ESI MS, and IR spectroscopy of the synthesized compounds.
