# Supplementary material for: Reactions of Nitroxides, Part 17. Synthesis, Fungistatic and Bacteriostatic Activity of Novel Five- and Six-Membered Nitroxyl Selenoureas and Selenocarbamates
Source: Molecules. 2019 Jul 4;24(13):2457. doi: 10.3390/molecules24132457 (PMC6650962; doi:10.3390/molecules24132457)
Supplement: Supplementary file 1 [file molecules-24-02457-s001.pdf]

Jerzy Zakrzewski,* Bogumiła Huras, Anna Kiełczewska, Maria Krawczyk, Jarosław Hupko, Katarzyna Jaszczuk

Reactions of nitroxides, Part 17. Synthesis, fungistatic and bacteriostatic activity of novel five- and six-membered nitroxide selenoureas and selenocarbamates

Supplementary Material

Spectroscopic data of synthesized nitroxide selenoureas 4 - 8, and nitroxide selenocarbamates 9, 10

File :C:\msdchem\1\data\marzec12\BS\Snapshot\BH_19_2012_.D
Operator : A. Kielczewska
Acquired : 6 Mar 2012 13:27 using AcqMethod DI250.m
Instrument : SIS_DIP-5975B
Sample Name: BH-19/2012
Misc Info :
Vial Number: 1

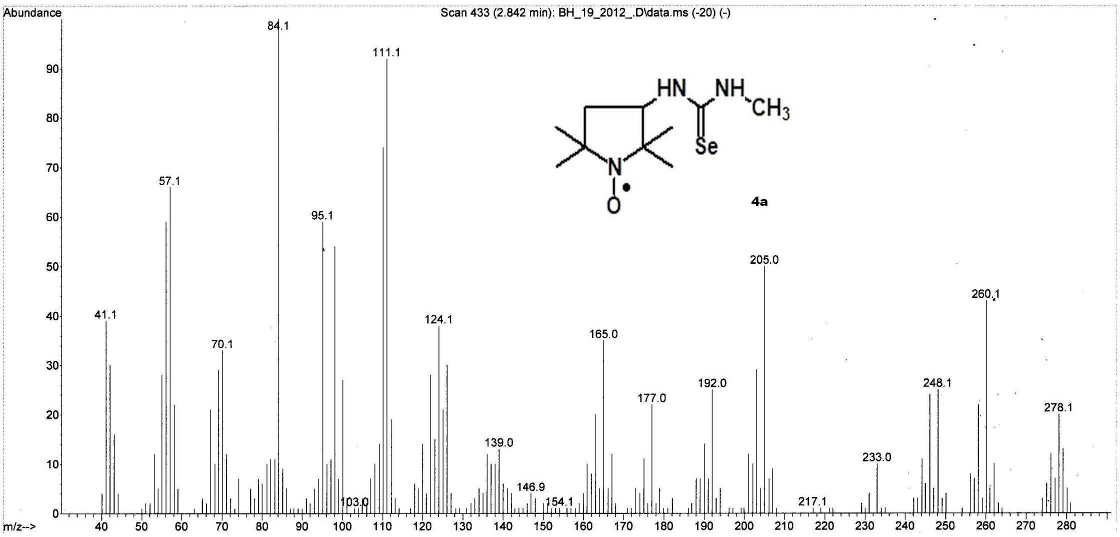

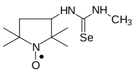

1-(2,2,5,5-Tetramethyl-1-oxyl-3-pyrrolidinyl)-3-methyl selenourea, **4a**, EI MS.

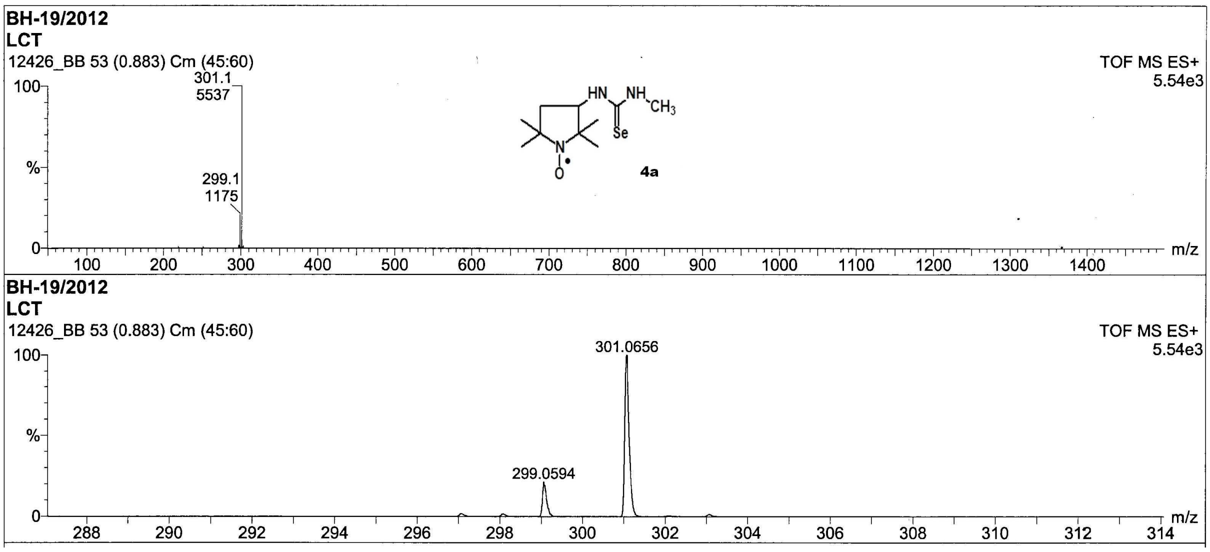

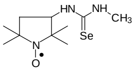

1-(2,2,5,5-Tetramethyl-1-oxyl-3-pyrrolidinyl)-3-methyl selenourea, **4a**, ESI MS.

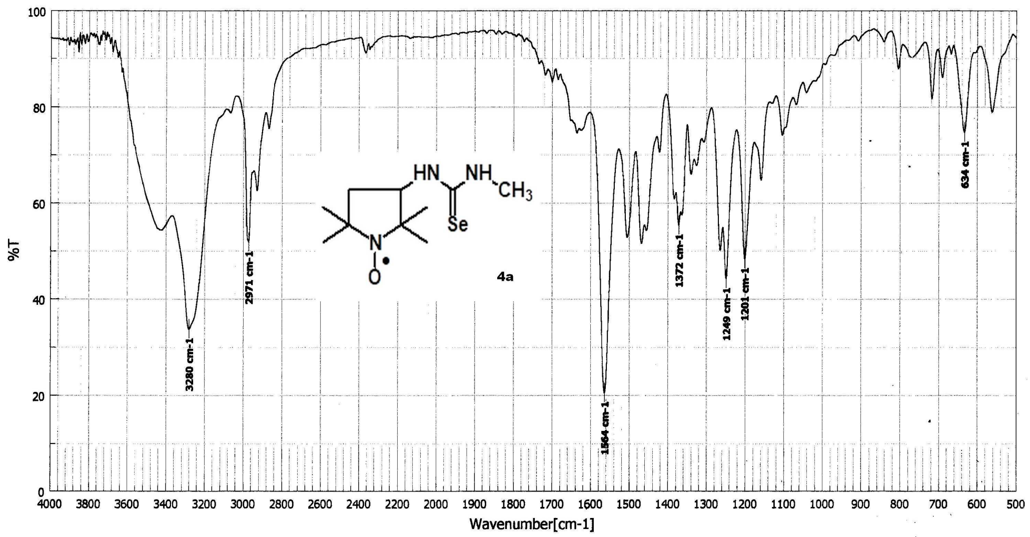

Sample Name	BH-19/2012 (0,5mg/270mg KBr)
Resolution	1 cm-1
Accumulation	30
Apodization	Cosine
Date/Time	112-02-21 13:41

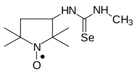

1-(2,2,5,5-Tetramethyl-1-oxyl-3-pyrrolidinyl)-3-methyl selenourea, **4a**, IR.

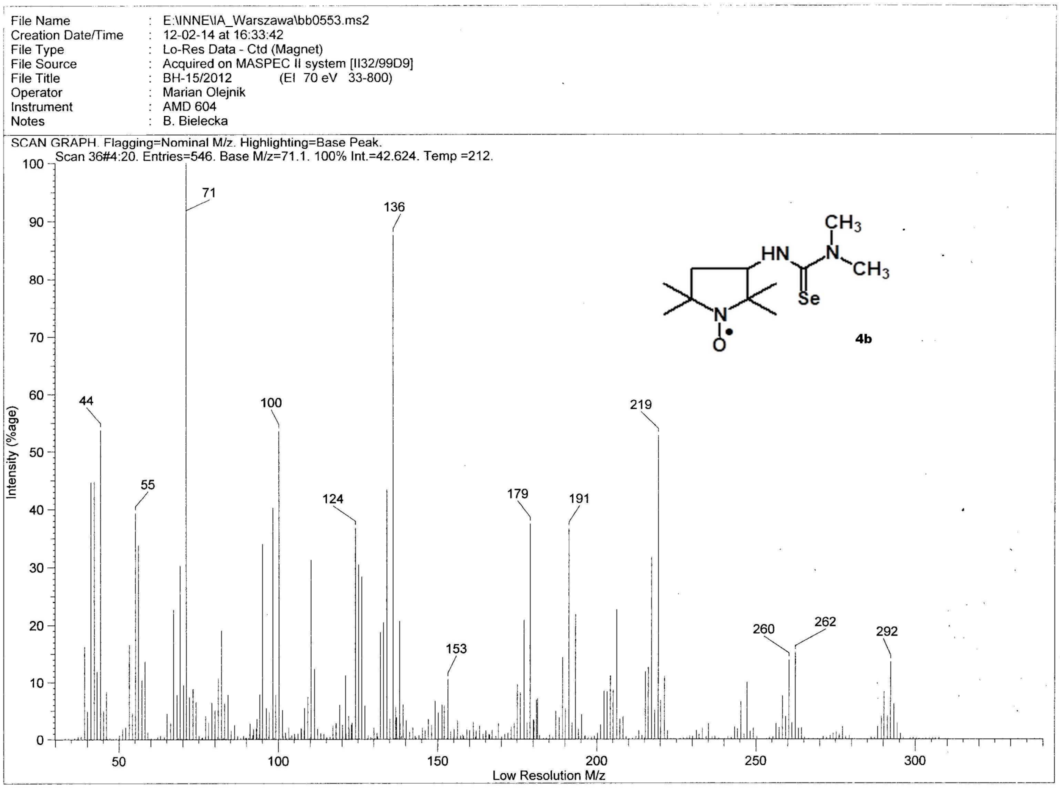

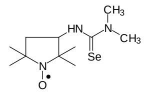

1-(2,2,5,5-Tetramethyl-1-oxyl-3-pyrrolidinyl)-3,3-dimethyl selenourea, **4b**, EI MS.

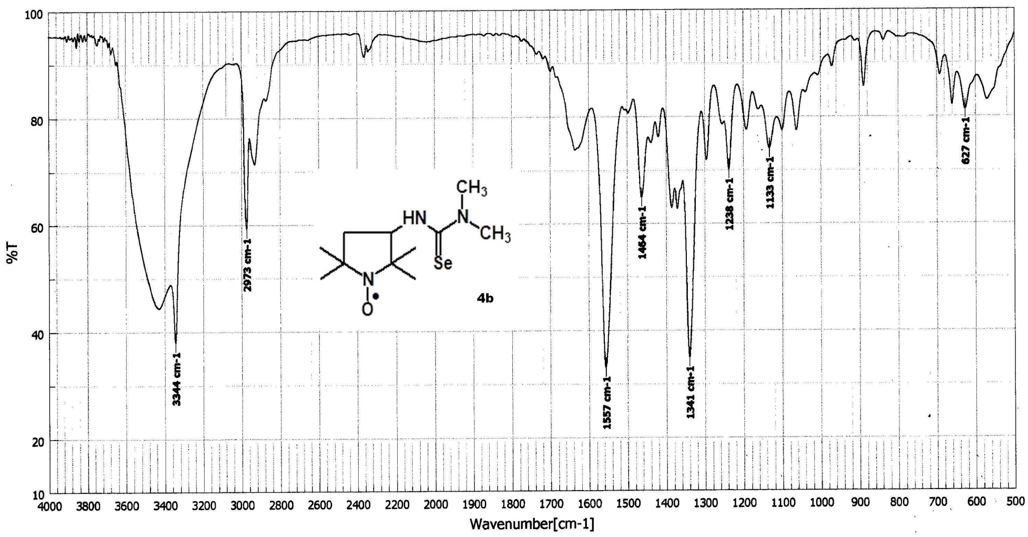


```
Sample Name         BH-15/2012  (0,4mg/270mg KBr)
Resolution          1 cm-1
Accumulation        30
Apodization         Cosine
Date/Time           112-02-08 9:08
```

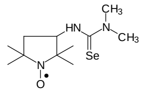

1-(2,2,5,5-Tetramethyl-1-oxyl-3-pyrrolidinyl)-3,3-dimethyl selenourea, **4b**, IR.

File :C:\msdchem\1\data\kwiecien2012\BS\BH_37_2012.D
Operator : A. Kielczewska
Acquired : 12 Apr 2012 9:56 using AcqMethod DI250.m
Instrument : SIS_DIP-5975B
Sample Name: BH/37/2012 B Huras
Misc Info :
Vial Number: 1

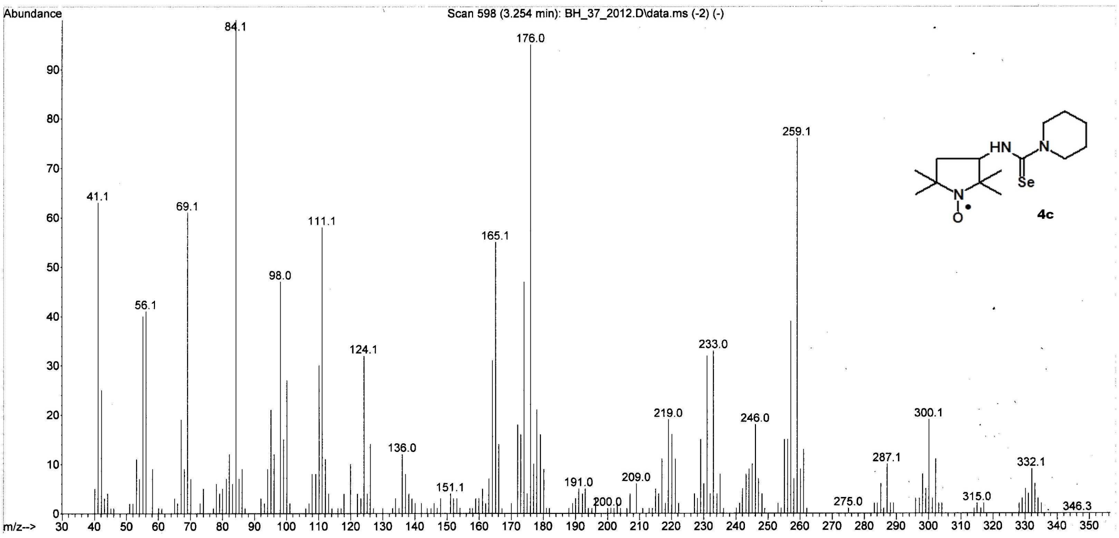

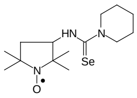

1-(2,2,5,5-Tetramethyl-1-oxyl-3-pyrrolidinyl)-3,3-pentyleno selenourea, **4c**, EI MS.

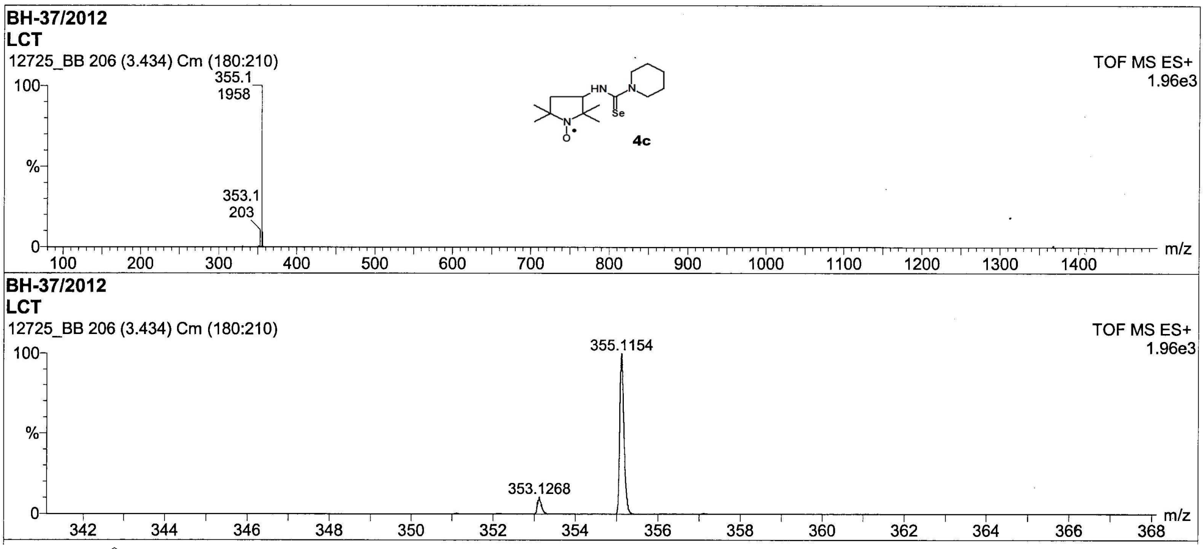

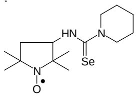

1-(2,2,5,5-Tetramethyl-1-oxyl-3-pyrrolidinyl)-3,3-pentyleno selenourea, **4c**, ESI MS.

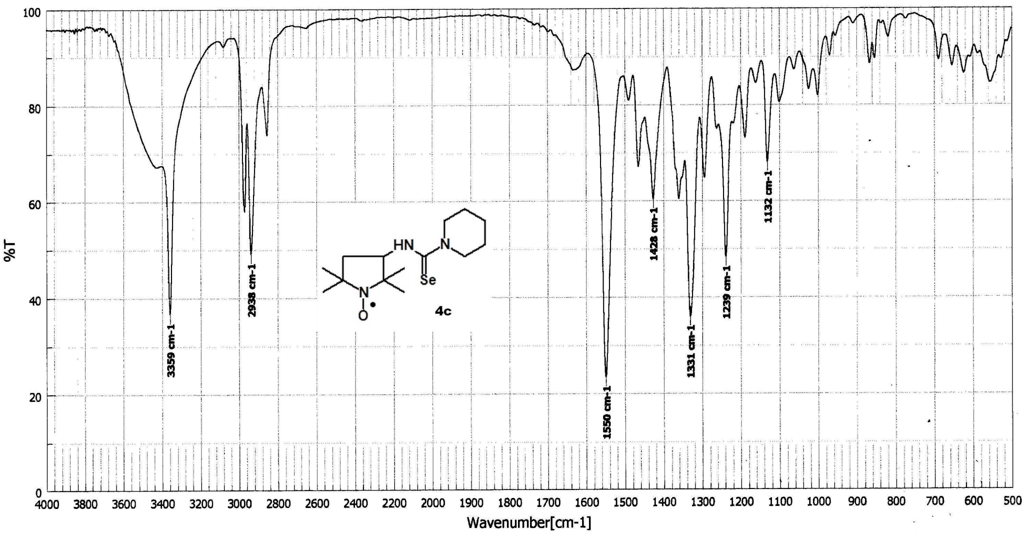

Sample Name	BH-37/2012 (0,5mg/270mg KBr)
Resolution	1 cm-1
Accumulation	30
Apodization	Cosine
Date/Time	112-04-13 10:15

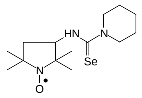

1-(2,2,5,5-Tetramethyl-1-oxyl-3-pyrrolidinyl)-3,3-pentyleno selenourea, **4c**, IR.

File :C:\msdchem\1\data\kwiecien2012\BS\BH_38_2012.D
Operator : A. Kielczewska
Acquired : 12 Apr 2012 10:26 using AcqMethod DI250.m
Instrument : SIS_DIP-5975B
Sample Name: BH/38/2012 B Huras
Misc Info :
Vial Number: 1

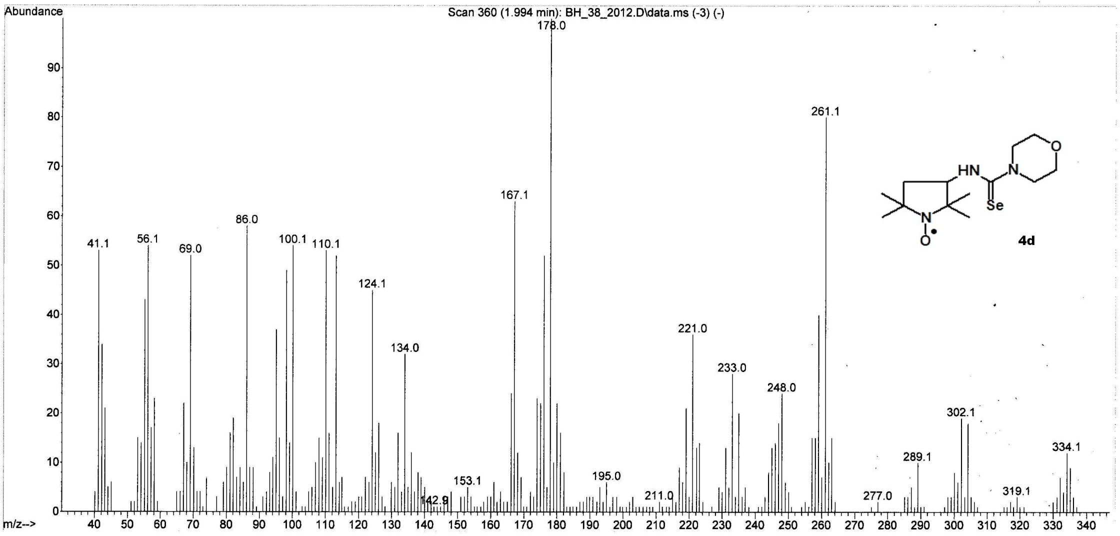

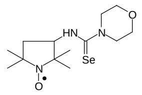

1-(2,2,5,5-Tetramethyl-1-oxyl-3-pyrrolidinyl)-3,3-(3-oksapentyleno) selenourea, **4d**, EI MS.

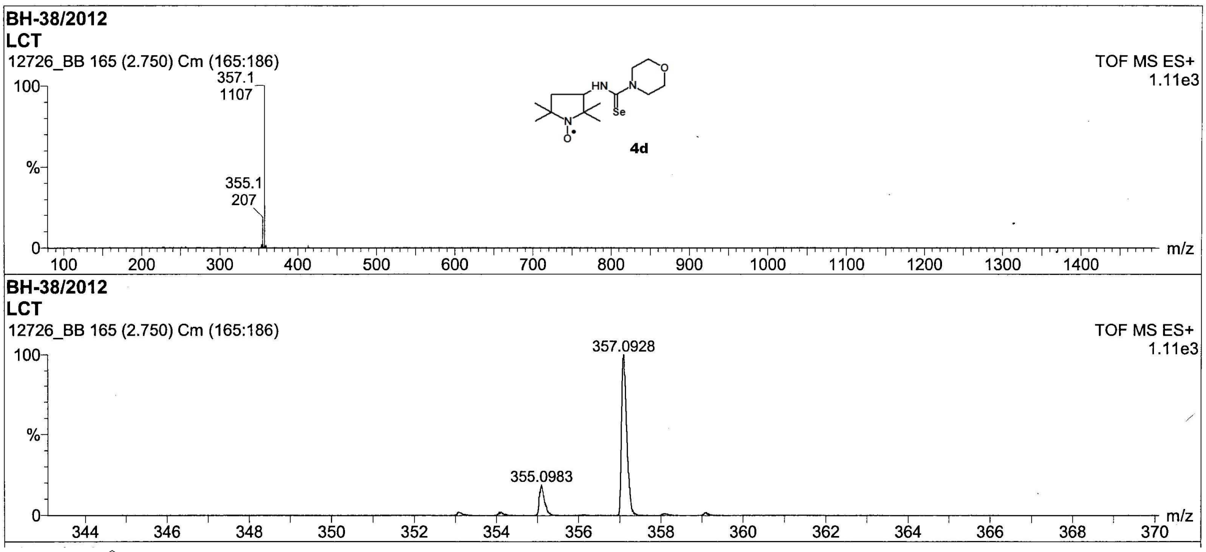

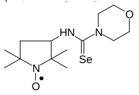

1-(2,2,5,5-Tetramethyl-1-oxyl-3-pyrrolidinyl)-3,3-(3-oksapentyleno) selenourea, **4d**, ESI MS.

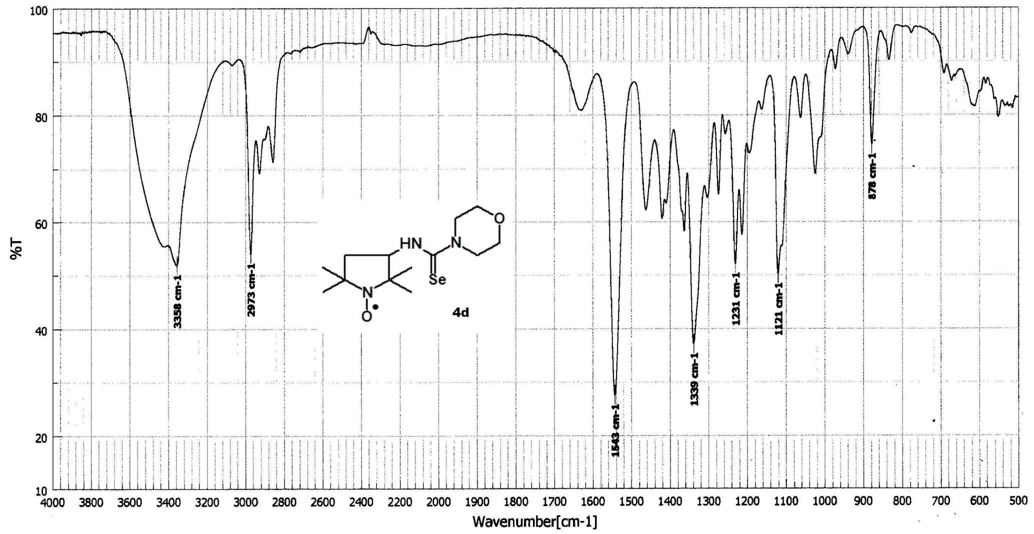


```
Sample Name        BH-38/2012 (0,5mg/270mg KBr)
Resolution         1 cm-1
Accumulation       30
Apodization        Cosine
Date/Time          112-04-13 11:17
```

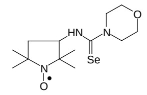

1-(2,2,5,5-Tetramethyl-1-oxyl-3-pyrrolidinyl)-3,3-(3-oksapentyleno) selenourea, **4d**, IR.

File :C:\msdchem\1\data\marzec12\BS\BH_20_2012.D
Operator : A Kielczewska
Acquired : 7 Mar 2012 11:28 using AcqMethod DI250.m
Instrument : SIS_DIP-5975B
Sample Name: BH/20/2012
Misc Info :
Vial Number: 1

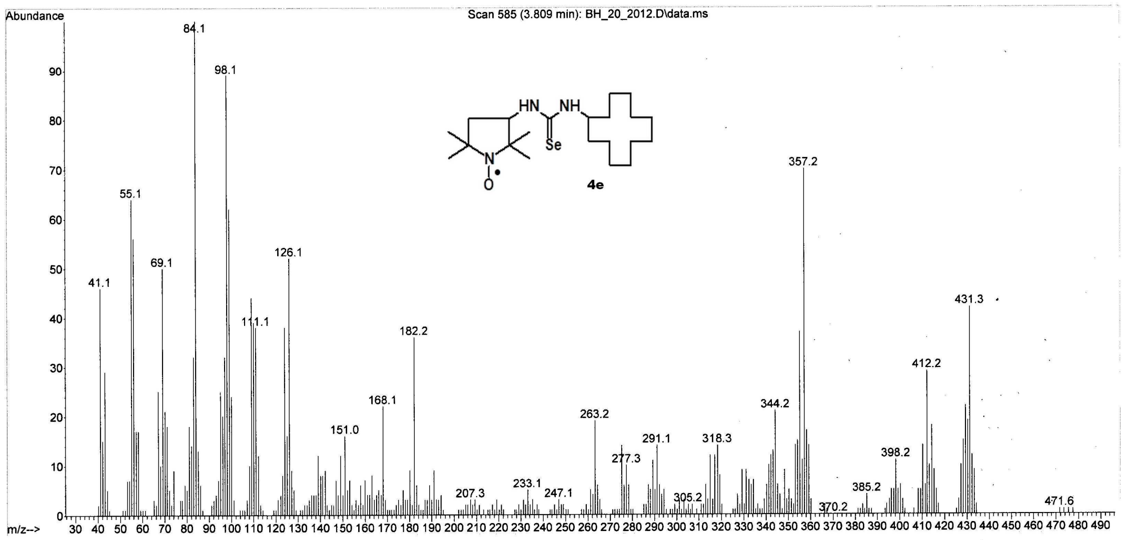

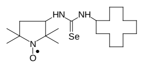

1-(2,2,5,5-Tetramethyl-1-oxyl-3-pyrrolidinyl)-3-cyclododecyl selenourea, **4e**, EI MS.

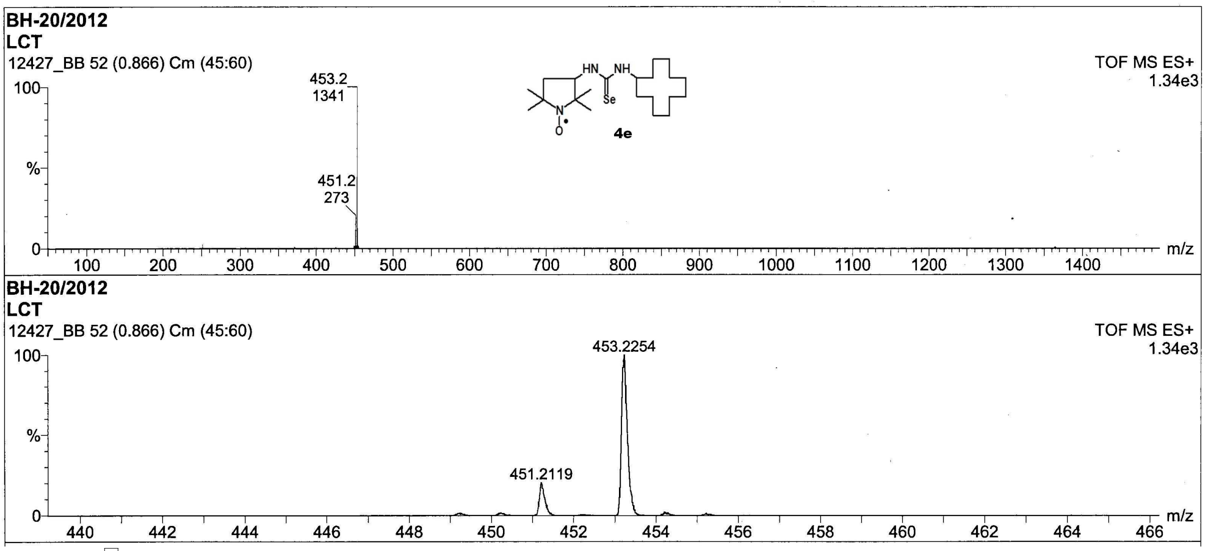

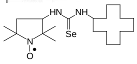

1-(2,2,5,5-Tetramethyl-1-oxyl-3-pyrrolidinyl)-3-cyclododecyl selenourea, **4e**, ESI MS.

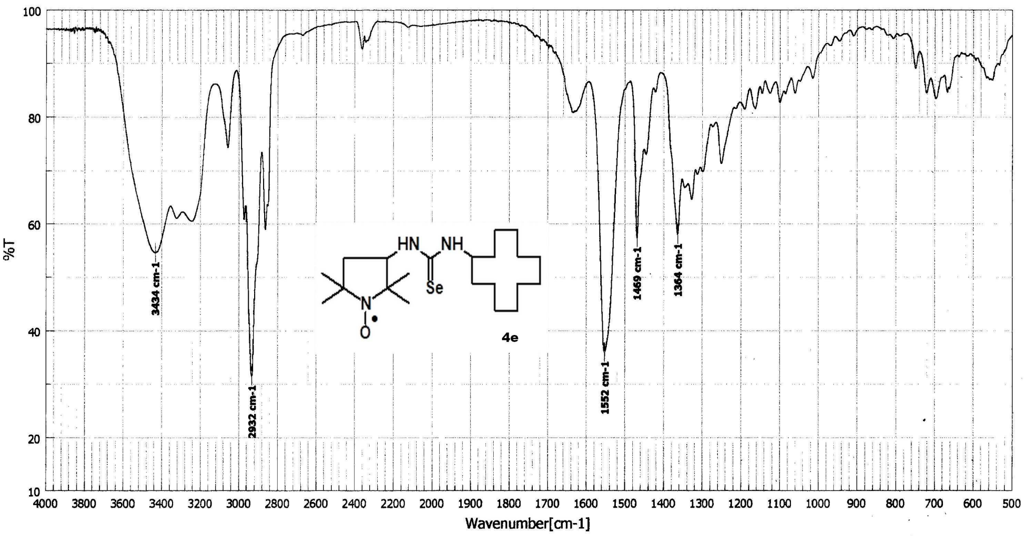


```
Sample Name        BH-20/2012/2  (0,2mg/270mg KBr)
Resolution         1 cm-1
Accumulation       30
Apodization        Cosine
Date/Time          112-02-27 9:59
```

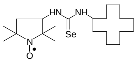

1-(2,2,5,5-Tetramethyl-1-oxyl-3-pyrrolidinyl)-3-cyclododecyl selenourea, **4e**, IR.

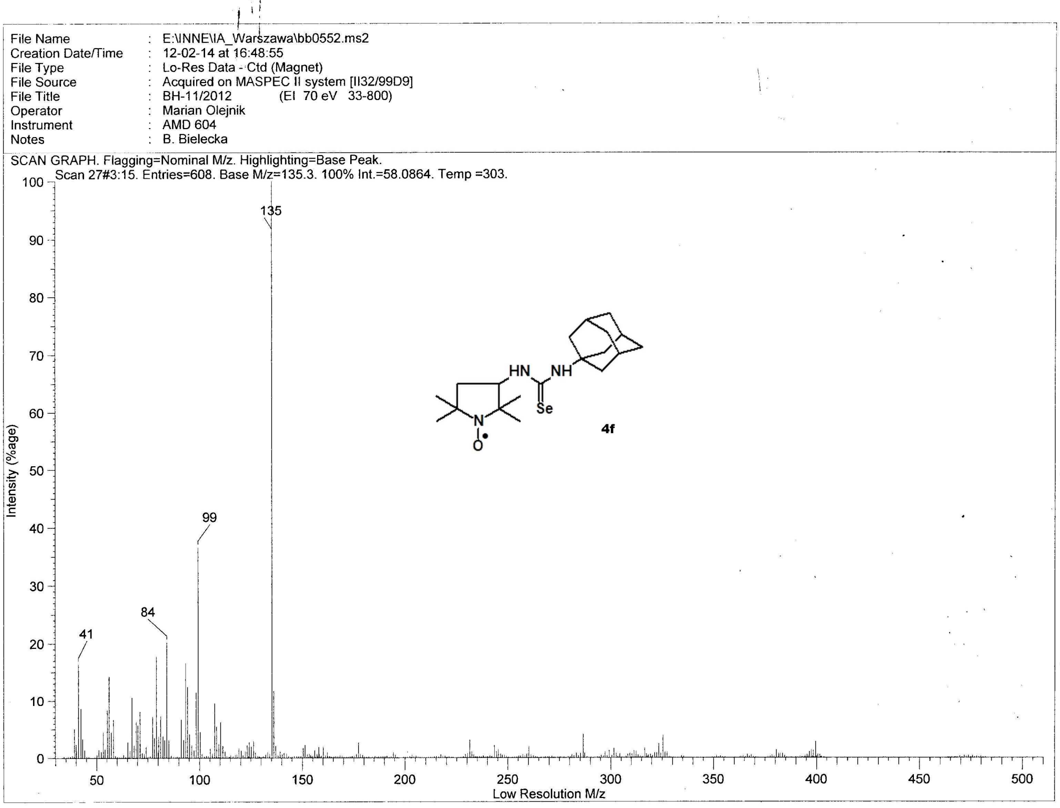

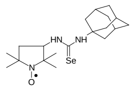

1-(2,2,5,5-Tetramethyl-1-oxyl-3-pyrrolidinyl)-3-(1-adamantyl) selenourea, **4f**, EI MS.

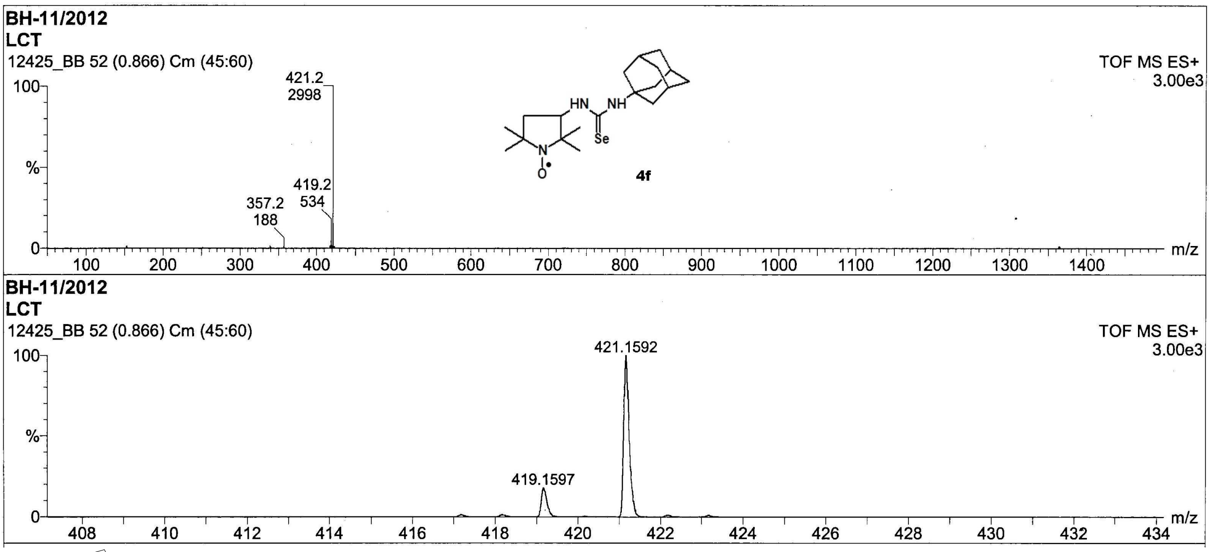

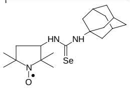

1-(2,2,5,5-Tetramethyl-1-oxyl-3-pyrrolidinyl)-3-(1-adamantyl) selenourea, **4f**, ESI MS.

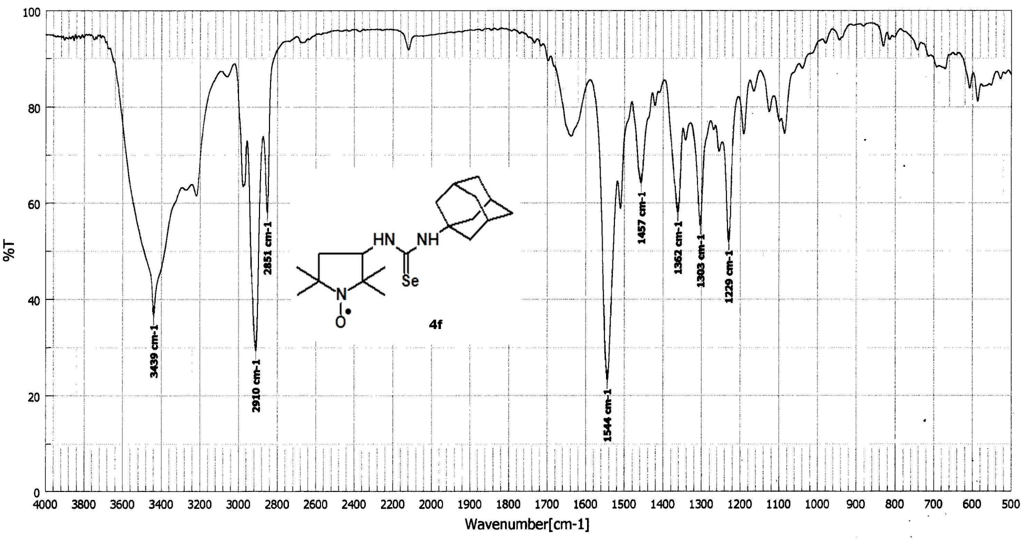

Sample Name	BH-11/2012/01 (0,5mg/270mg KBr)
Resolution	1 cm-1
Accumulation	30
Apodization	Cosine
Date/Time	112-02-06 10:09

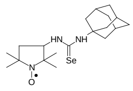

1-(2,2,5,5-Tetramethyl-1-oxyl-3-pyrrolidinyl)-3-(1-adamantyl) selenourea, **4f**, IR.

File :C:\msdchem\1\data\marzec12\BS\BH_30_2012.D
Operator : A Kielczewska
Acquired : 23 Mar 2012 9:12 using AcqMethod DI250.m
Instrument : SIS_DIP-5975B
Sample Name: BH/30/2012
Misc Info :
Vial Number: 1

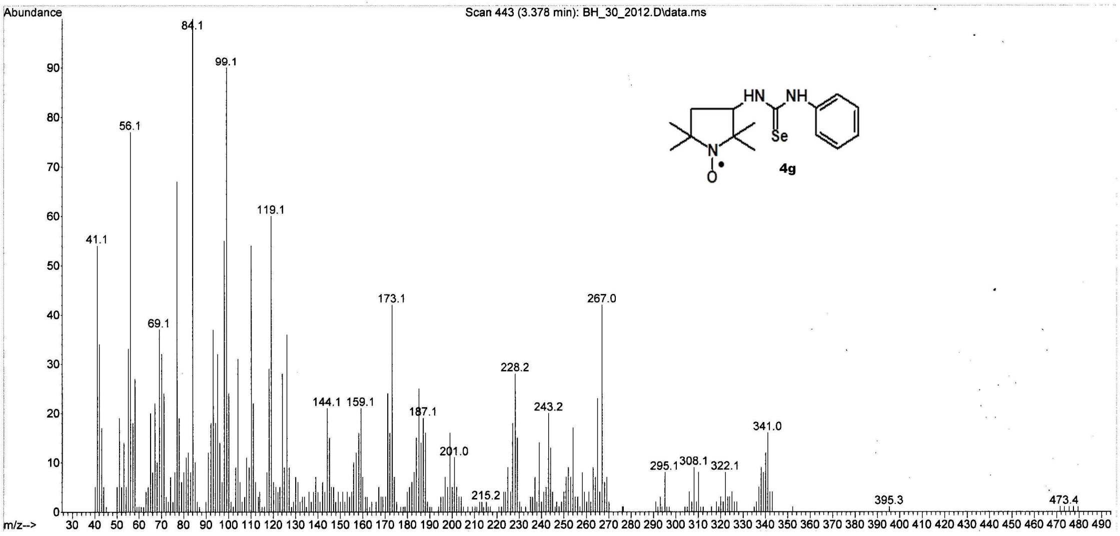

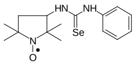

1-(2,2,5,5-Tetramethyl-1-oxyl-3-pyrrolidinyl)-3-phenyl selenourea, **4g**, EI MS.

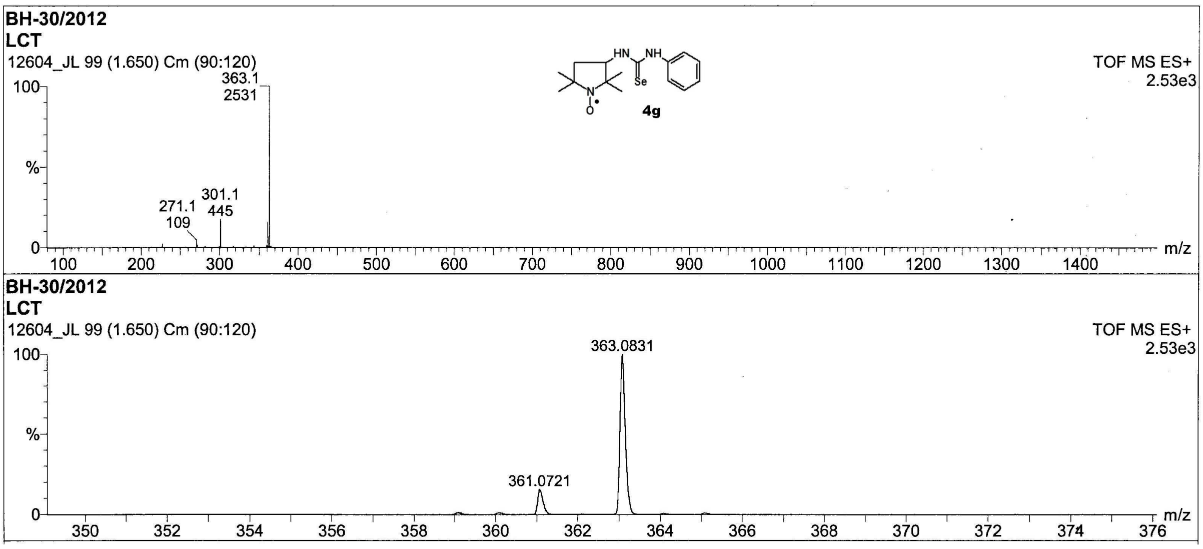

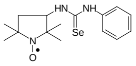

1-(2,2,5,5-Tetramethyl-1-oxyl-3-pyrrolidinyl)-3-phenyl selenourea, **4g**, ESI MS.

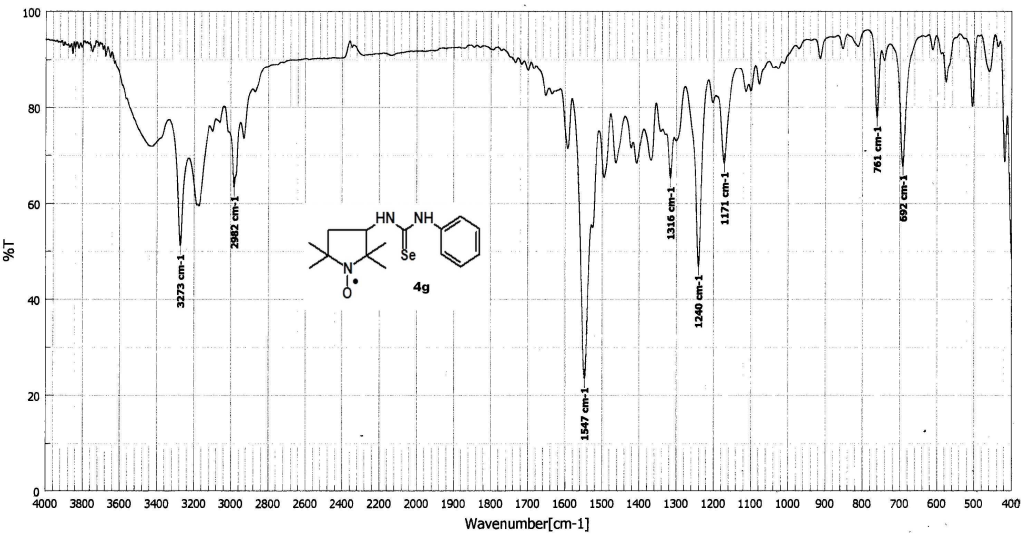

Sample Name	BH-30/2012 (0,5mg/270mg KBr)
Resolution	1 cm-1
Accumulation	30
Apodization	Cosine
Date/Time	113-10-28 10:55

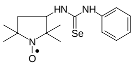

1-(2,2,5,5-Tetramethyl-1-oxyl-3-pyrrolidinyl)-3-phenyl selenourea, **4g**, IR.

File :C:\msdchem\1\data\marzec12\BS\BH_23_2012.D
Operator : A Kielczewska
Acquired : 8 Mar 2012 12:05 using AcqMethod DI250.m
Instrument : SIS_DIP-5975B
Sample Name: BH/23/2012
Misc Info :
Vial Number: 1

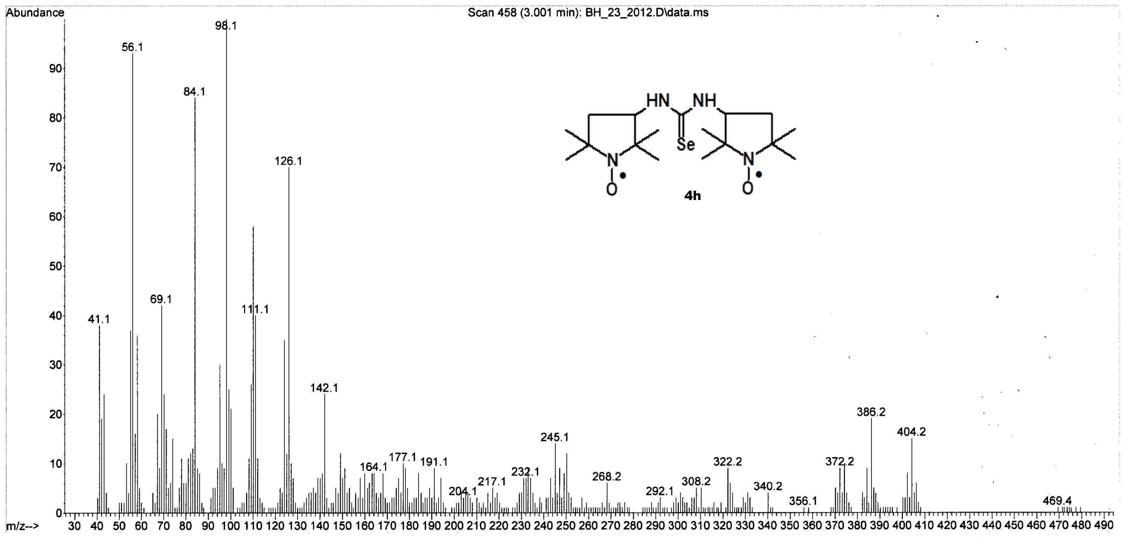

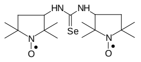
1,3-Bis(2,2,5,5-tetramethyl-1-oxyl-3-pyrrolidinyl) selenourea, **4h**, EI MS.

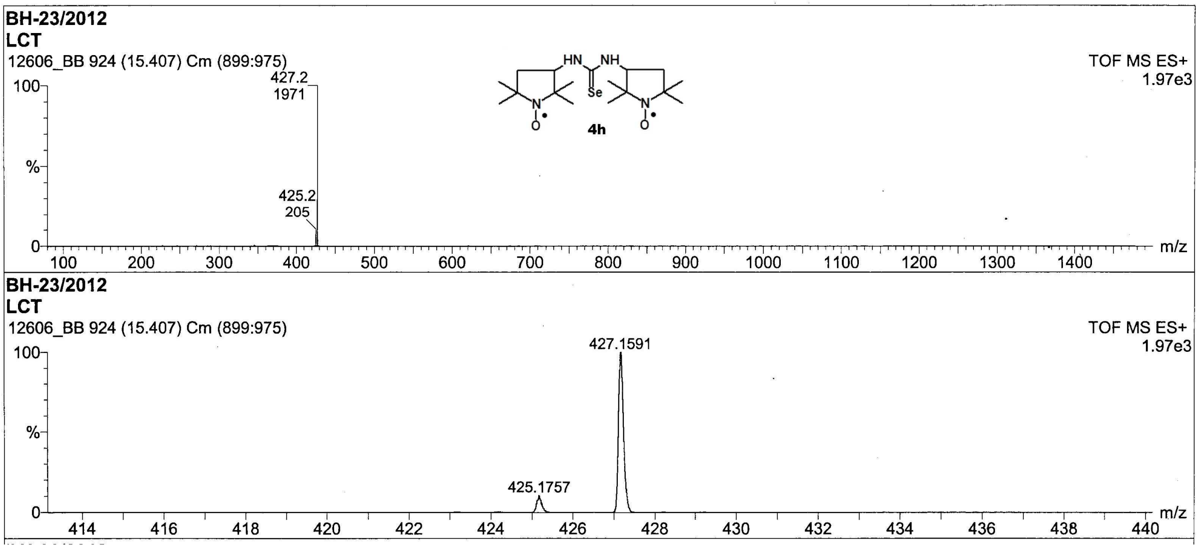

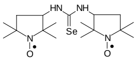

1,3-Bis(2,2,5,5-tetramethyl-1-oxyl-3-pyrrolidinyl) selenourea, **4h**, ESI MS.

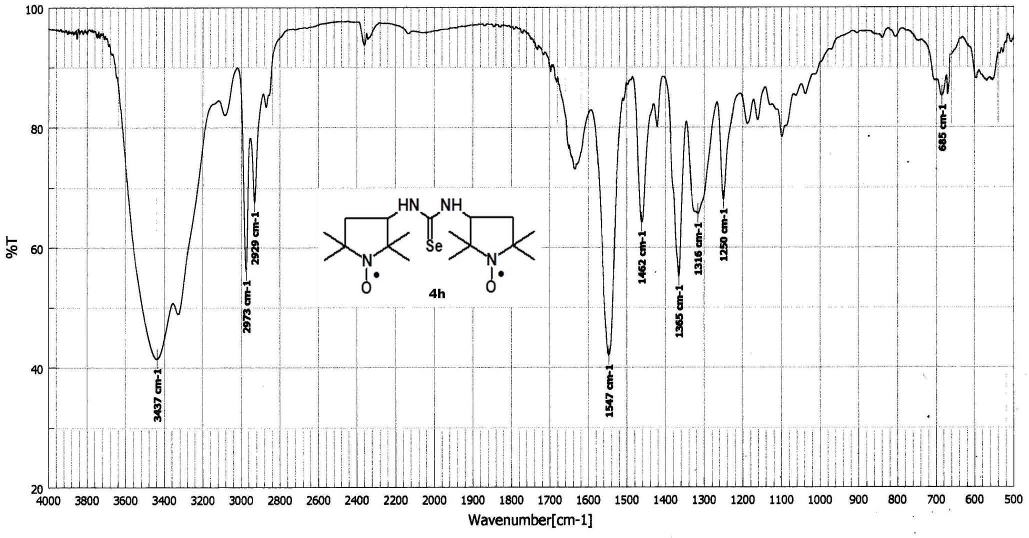

Sample Name	BH-23/2012 (0,4mg/270mg KBr)
Resolution	1 cm-1
Accumulation	30
Apodization	Cosine
Date/Time	112-03-13 10:17

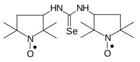

1,3-Bis(2,2,5,5-tetramethyl-1-oxyl-3-pyrrolidinyl) selenourea, **4h**, IR.

File :C:\msdchem\1\data\kwiecien2012\BS\BH_35_2012_.D
Operator : A Kielczewska
Acquired : 3 Apr 2012 9:37 using AcqMethod DI250.m
Instrument : SIS_DIP-5975B
Sample Name: BH-35/2012
Misc Info :
Vial Number: 1

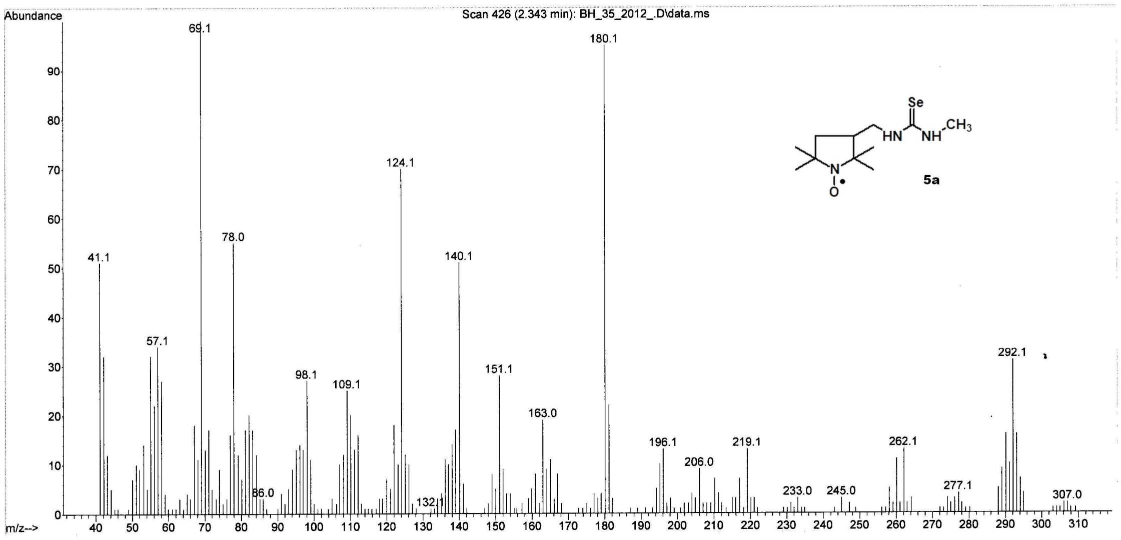

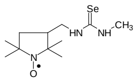

1-[(2,2,5,5-Tetramethyl-1-oxyl-3-pyrrolidinyl)methyl]-3-methyl selenourea, **5a**, EI MS.

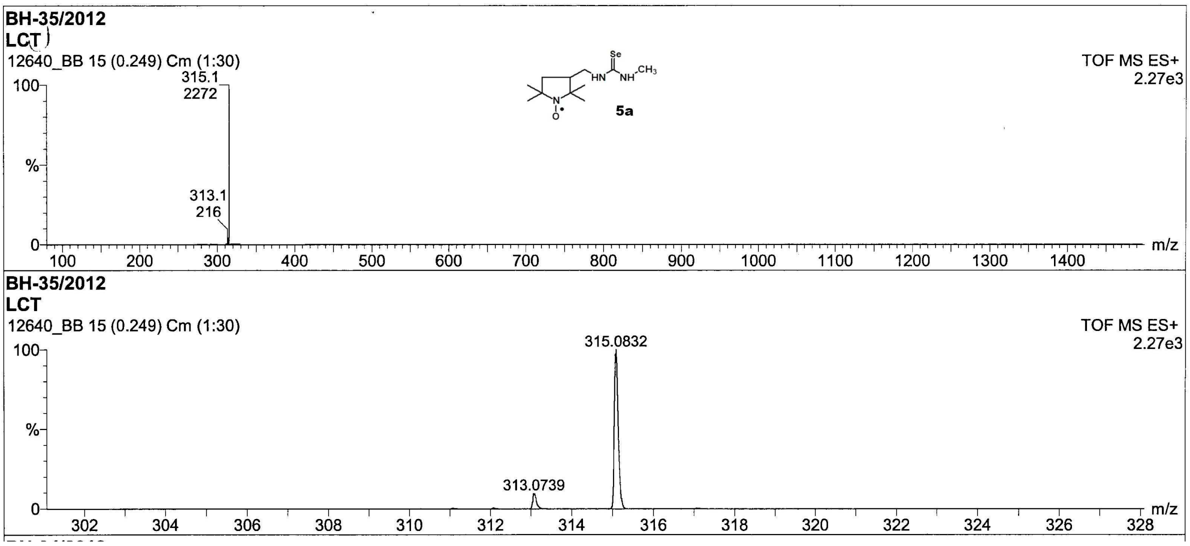

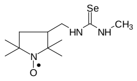 1-[(2,2,5,5-Tetramethyl-1-oxyl-3-pyrrolidinyl)methyl]-3-methyl selenourea, **5a**, ESI MS.

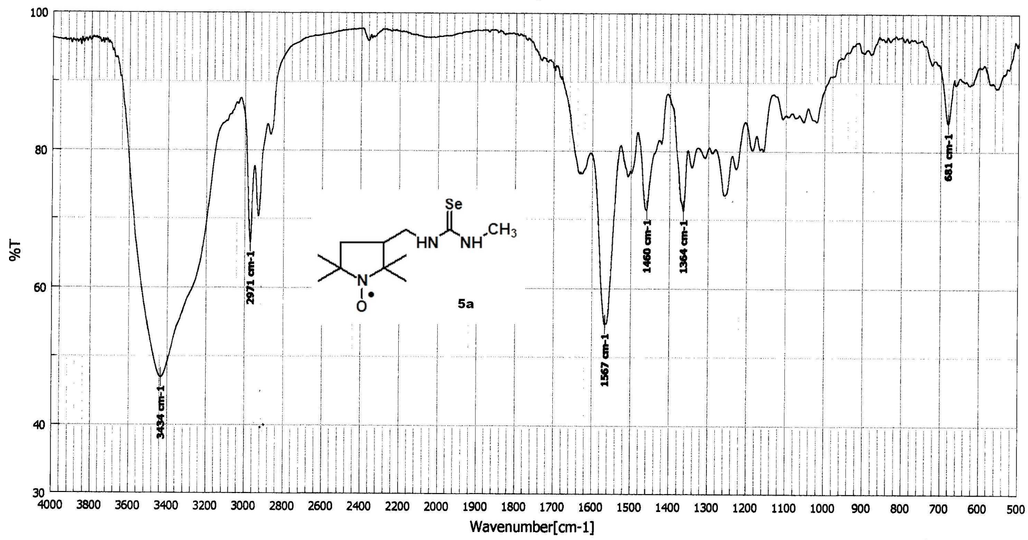

Sample Name	BH-35/2012 (0,4mg/270mgKBr)
Resolution	1 cm-1
Accumulation	30
Apodization	Cosine
Date/Time	112-04-03 9:42

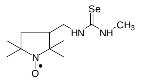

1-[(2,2,5,5-Tetramethyl-1-oxyl-3-pyrrolidinyl)methyl]-3-methyl selenourea, **5a**, IR.

File :C:\msdchem\1\data\kwiecien2012\BS\BH_34_2012.D
Operator : A Kielczewska
Acquired : 2 Apr 2012 13:42 using AcqMethod DI250.m
Instrument : SIS_DIP-5975B
Sample Name: BH/34/2012
Misc Info :
Vial Number: 1

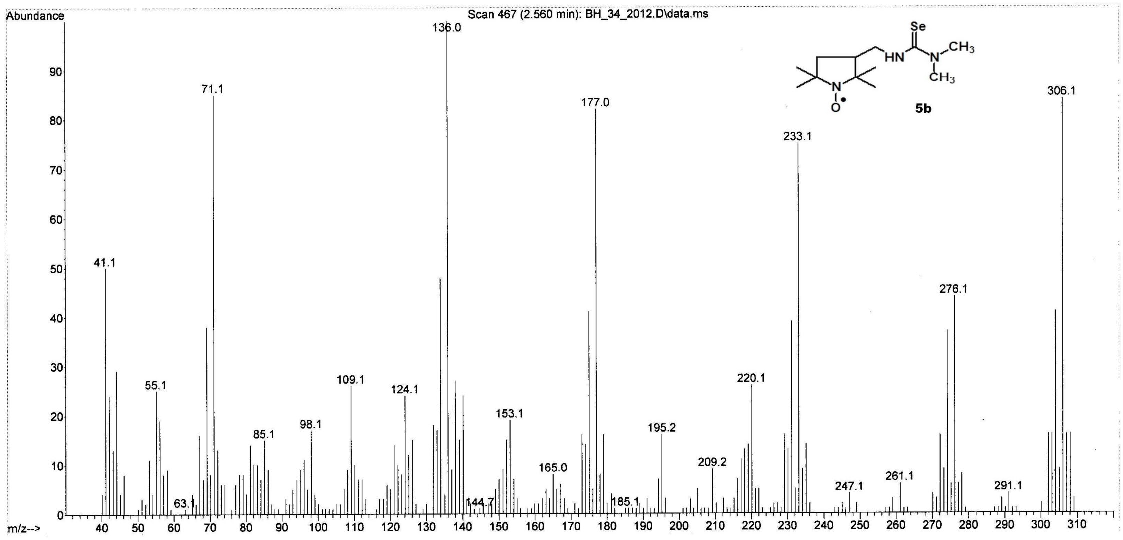

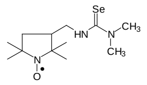

1-[(2,2,5,5-Tetramethyl-1-oxyl-3-pyrrolidinyl)methyl]-3,3-dimethyl selenourea, **5b**, EI MS.

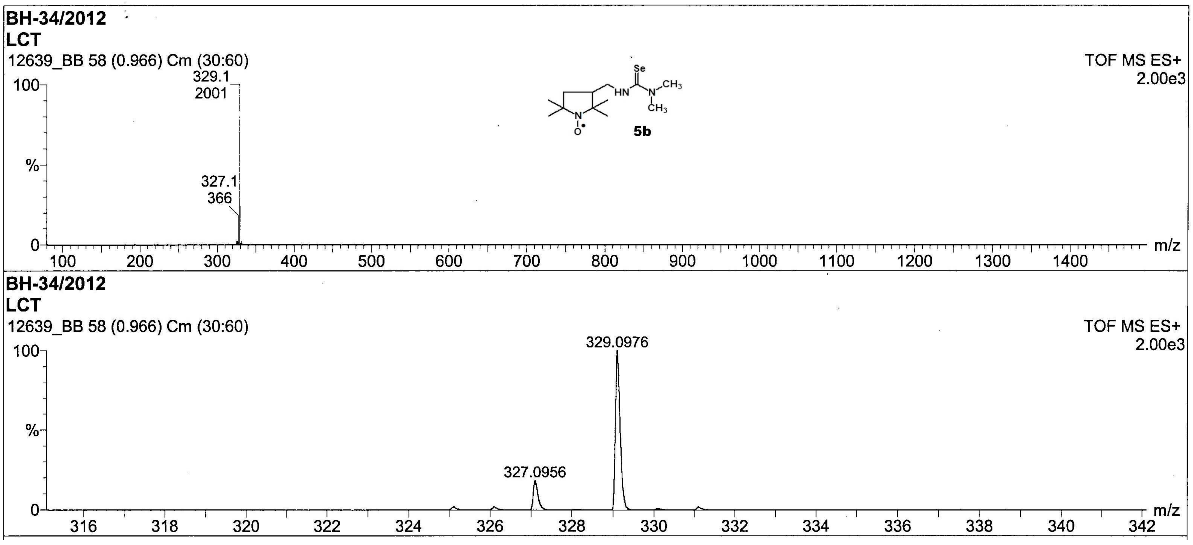

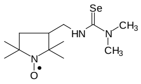

1-[(2,2,5,5-Tetramethyl-1-oxyl-3-pyrrolidinyl)methyl]-3,3-dimethyl selenourea, **5b**, ESI MS.

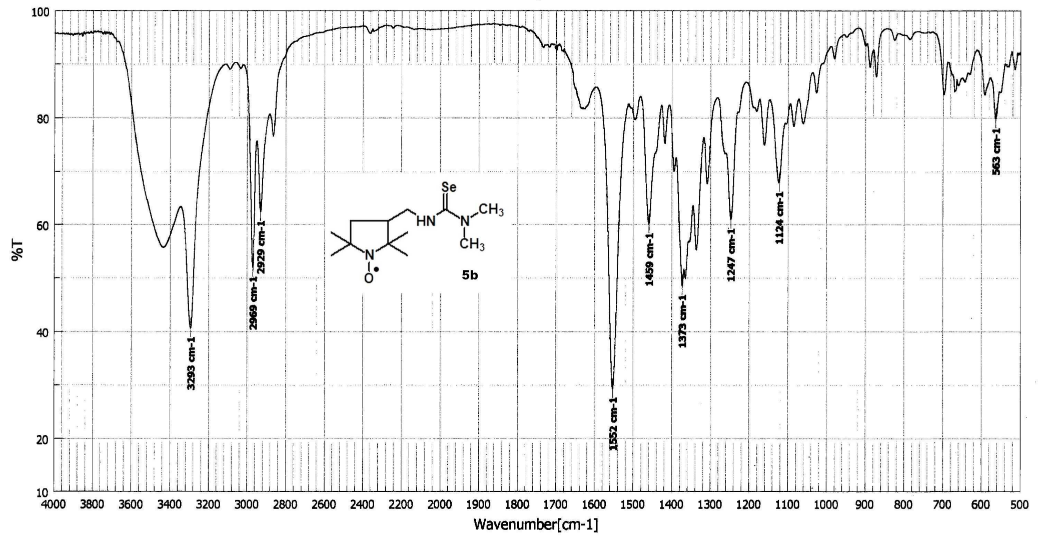

Sample Name	BH-34/2012 (0,5mg/270mg KBr)
Resolution	1 cm-1
Accumulation	30
Apodization	Cosine
Date/Time	112-04-02 12:56

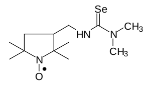

1-[(2,2,5,5-Tetramethyl-1-oxyl-3-pyrrolidinyl)methyl]-3,3-dimethyl selenourea, **5b**, IR.

File :C:\msdchem\1\data\maj2012\BS\BH_44_2012.D
Operator : A. Kielczewska
Acquired : 17 May 2012 12:48 using AcqMethod DI250.m
Instrument : SIS_DIP-5975B
Sample Name: BH/44/2012
Misc Info :
Vial Number: 1

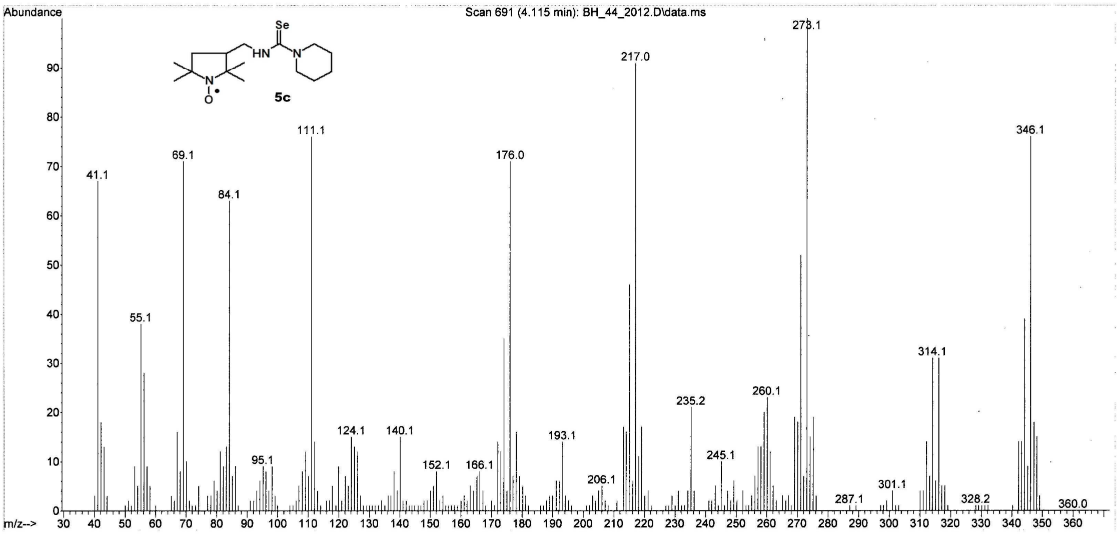

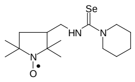

1-[(2,2,5,5-Tetramethyl-1-oxyl-3-pyrrolidinyl)methyl]-3,3-pentyleno selenourea, **5c**, EI MS.

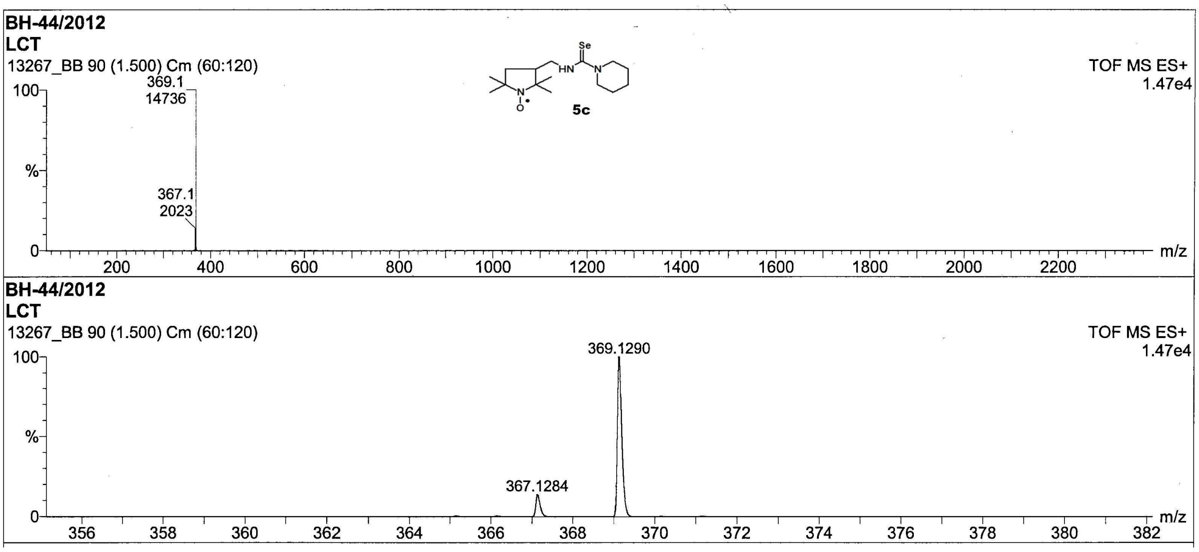

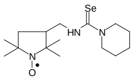 1-[(2,2,5,5-Tetramethyl-1-oxyl-3-pyrrolidinyl)methyl]-3,3-pentyleno selenourea, **5c**, ESI MS.

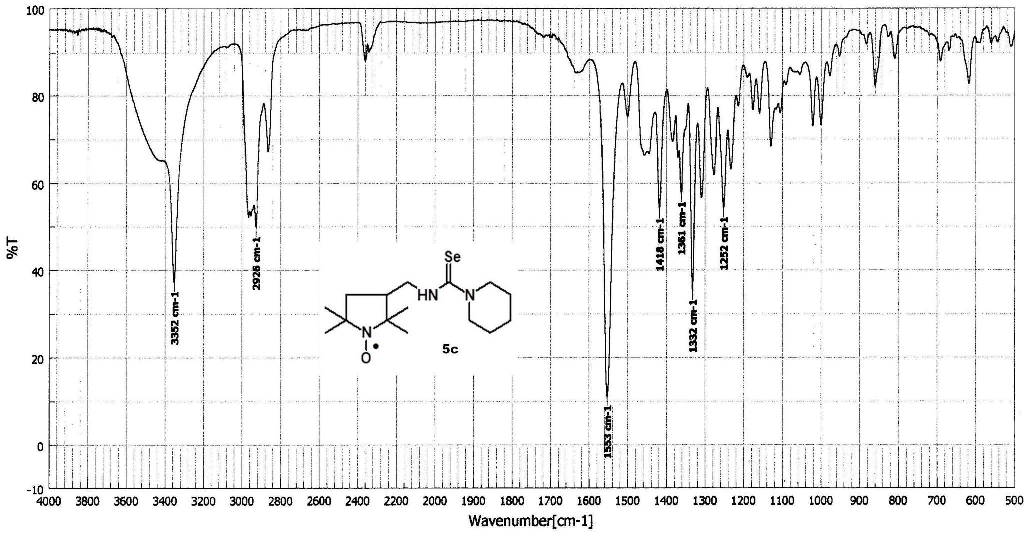

Sample Name	BH-44/2012 (0,6mg/270mg KBr)
Resolution	1 cm-1
Accumulation	30
Apodization	Cosine
Date/Time	112-05-21 13:42

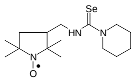

1-[(2,2,5,5-Tetramethyl-1-oxyl-3-pyrrolidinyl)methyl]-3,3-pentyleno selenourea, **5c**, IR.

File :C:\msdchem\1\data\maj2012\BS\BH_43_2012.D
Operator : A. Kielczewska
Acquired : 17 May 2012 11:07 using AcqMethod DI250.m
Instrument : SIS_DIP-5975B
Sample Name: BH/43/2012
Misc Info :
Vial Number: 1

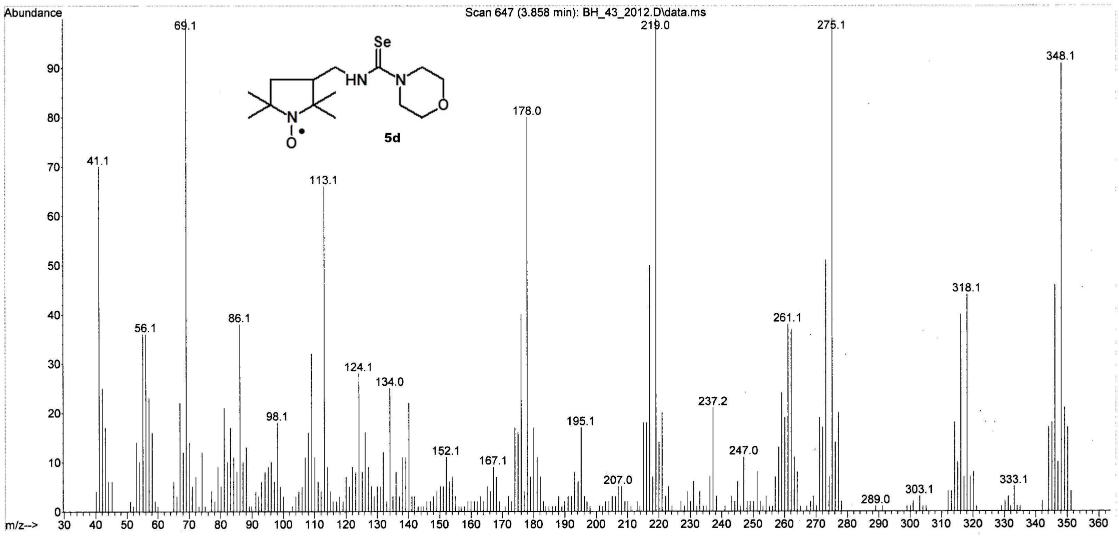

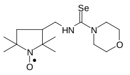

1-[(2,2,5,5-Tetramethyl-1-oxyl-3-pyrrolidinyl)methyl]-3,3-(3-oksapentyleno) selenourea, **5d**, EI MS.

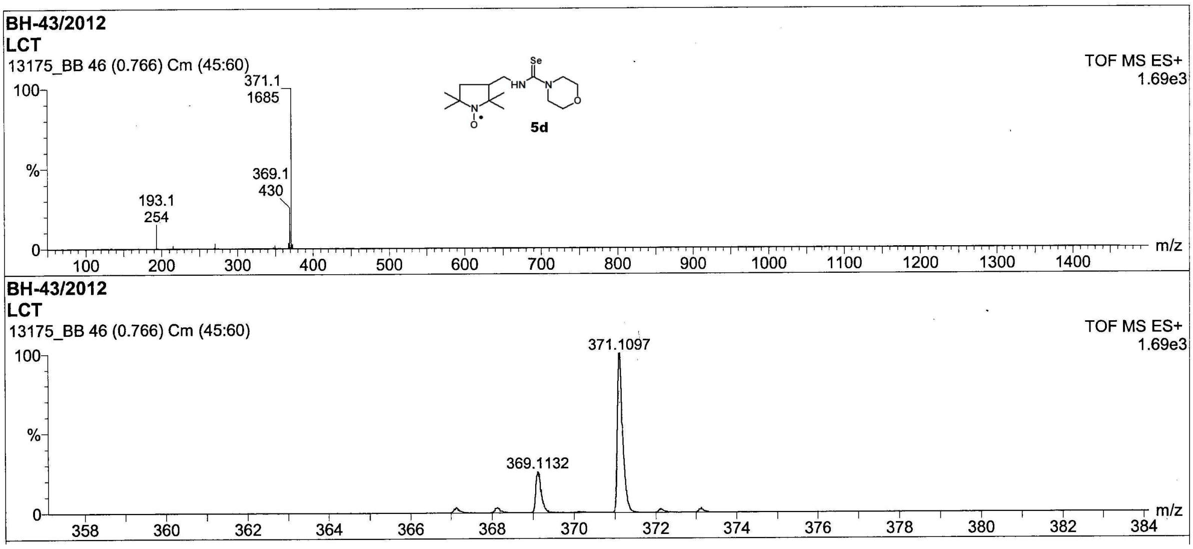

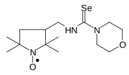

1-[(2,2,5,5-Tetramethyl-1-oxyl-3-pyrrolidinyl)methyl]-3,3-(3-oksapentyleno) selenourea, **5d**, ESI MS.

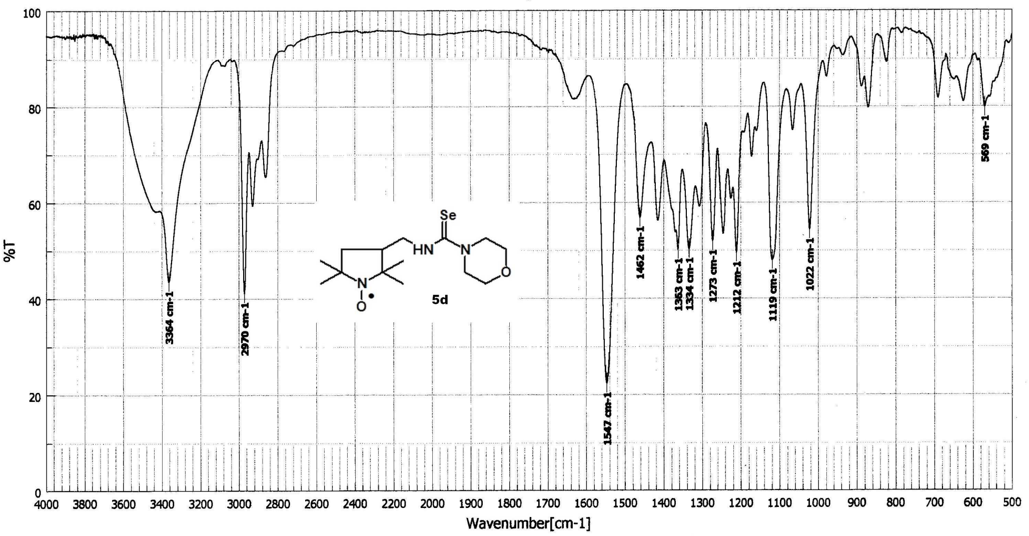

Sample Name	BH-43/2012 (0,6mg/270mg KBr)
Resolution	1 cm-1
Accumulation	30
Apodization	Cosine
Date/Time	112-05-09 8:42

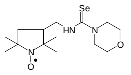 1-[(2,2,5,5-Tetramethyl-1-oxyl-3-pyrrolidinyl)methyl]-3,3-(3-oksapentyleno) selenourea, **5d**, IR.

File :C:\msdchem\1\data\maj2012\BS\BH_49_2012.D
Operator : A. Kielczewska
Acquired : 17 May 2012 13:52 using AcqMethod DI250.m
Instrument : SIS_DIP-5975B
Sample Name: BH/49/2012
Misc Info :
Vial Number: 1

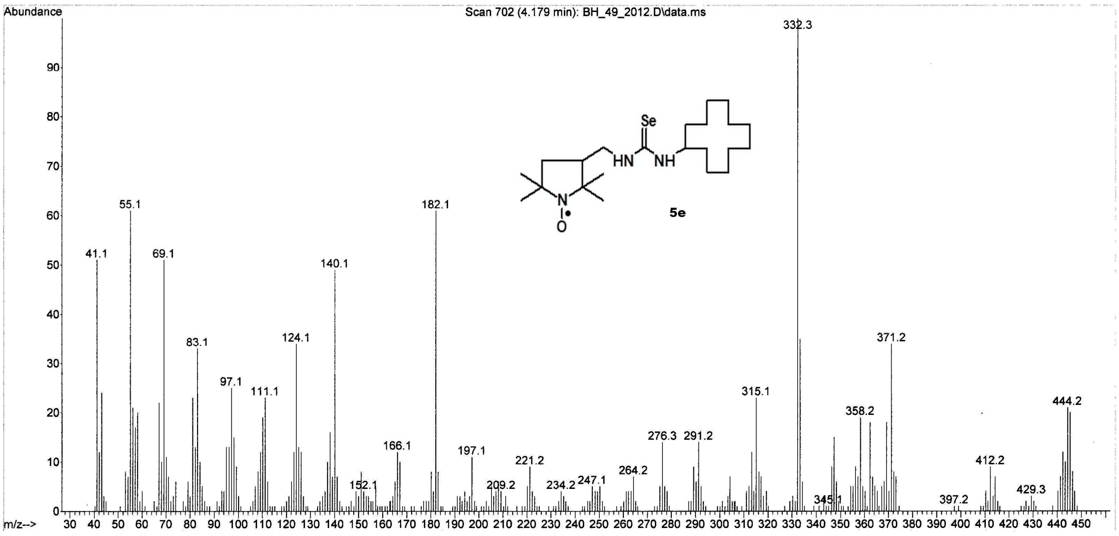

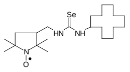
1-[(2,2,5,5-Tetramethyl-1-oxyl-3-pyrrolidinyl)methyl]-3-cyclododecyl selenourea, **5e**, EI MS.

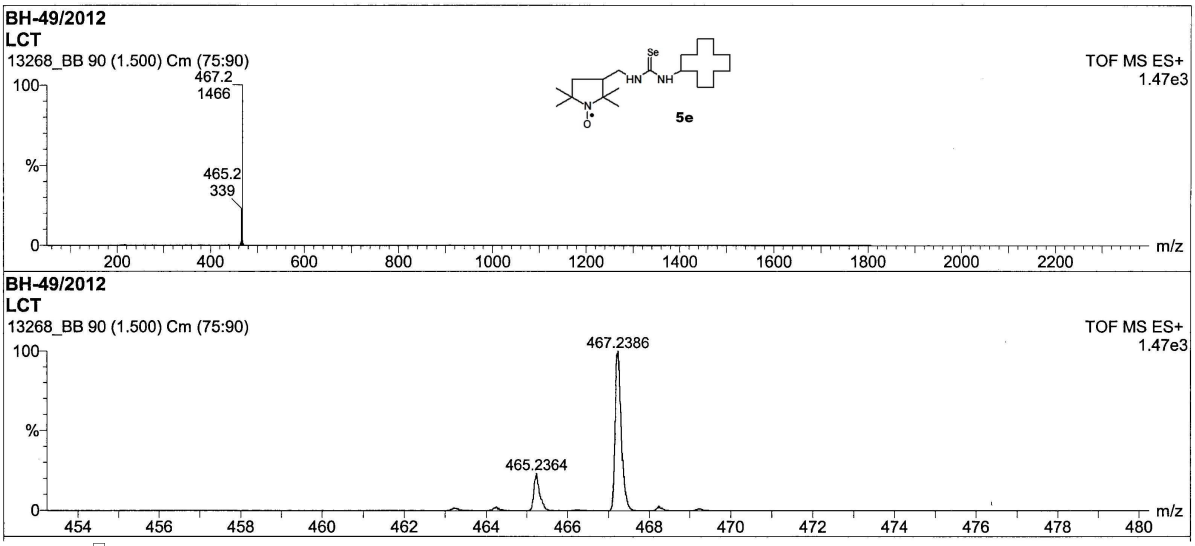

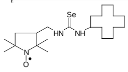 1-[(2,2,5,5-Tetramethyl-1-oxyl-3-pyrrolidinyl)methyl]-3-cyclododecyl selenourea, **5e**, ESI MS.

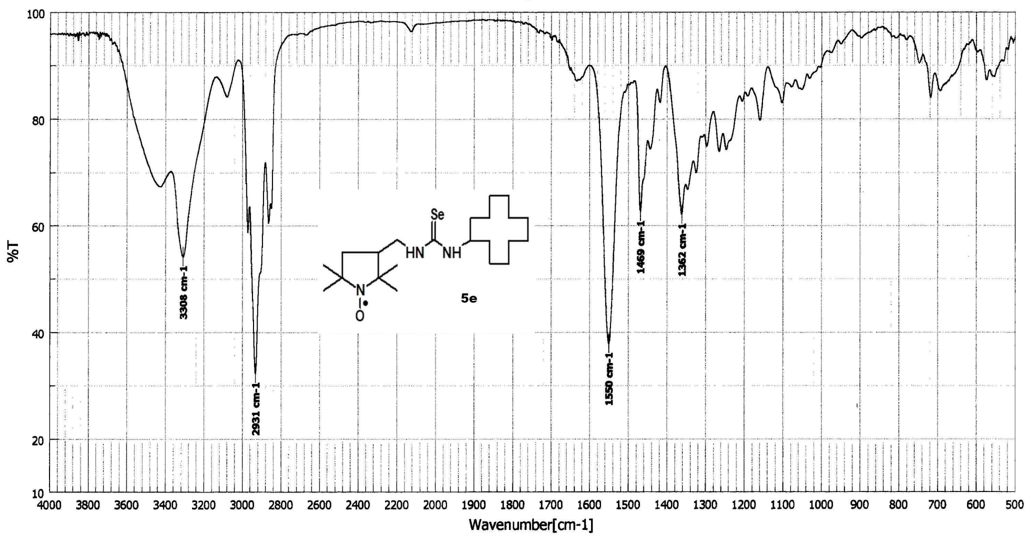

Sample Name	BH-49/2012 (0,5mg/270mg KBr)
Resolution	1 cm-1
Accumulation	30
Apodization	Cosine
Date/Time	112-05-21 14:50

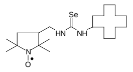

1-[(2,2,5,5-Tetramethyl-1-oxyl-3-pyrrolidinyl)methyl]-3-cyclododecyl selenourea, **5e**, IR.

File :C:\msdchem\1\data\kwiecien2012\BS\BH_36_2012.D
Operator : A. Kielczewska
Acquired : 12 Apr 2012 11:12 using AcqMethod DI250.m
Instrument : SIS_DIP-5975B
Sample Name: BH/36/2012 B Huras
Misc Info :
Vial Number: 1

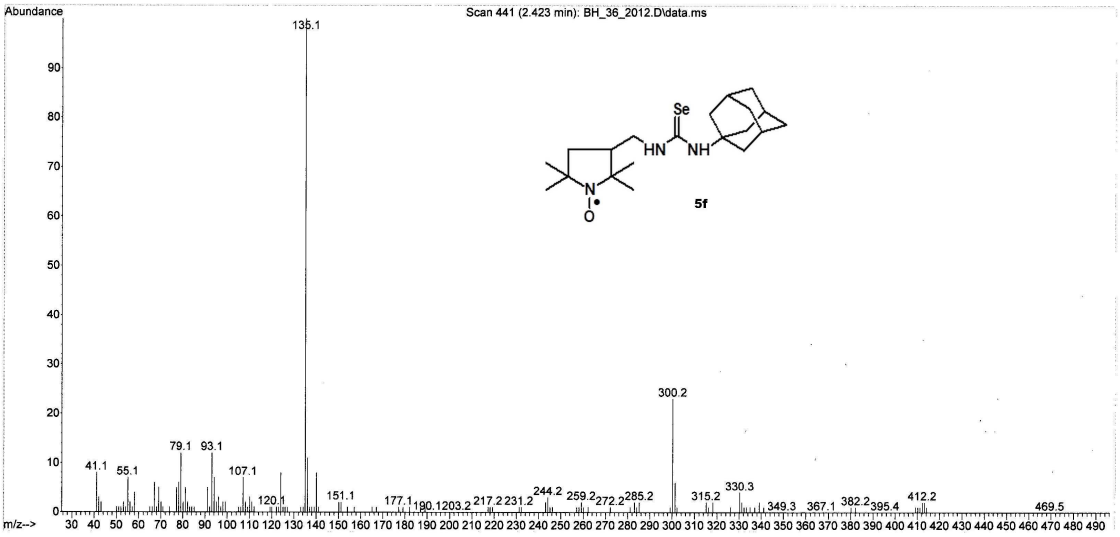

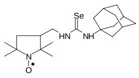

1-[(2,2,5,5-Tetramethyl-1-oxyl-3-pyrrolidinyl)methyl]-3-(1-adamantyl) selenourea, **5f**, EI MS.

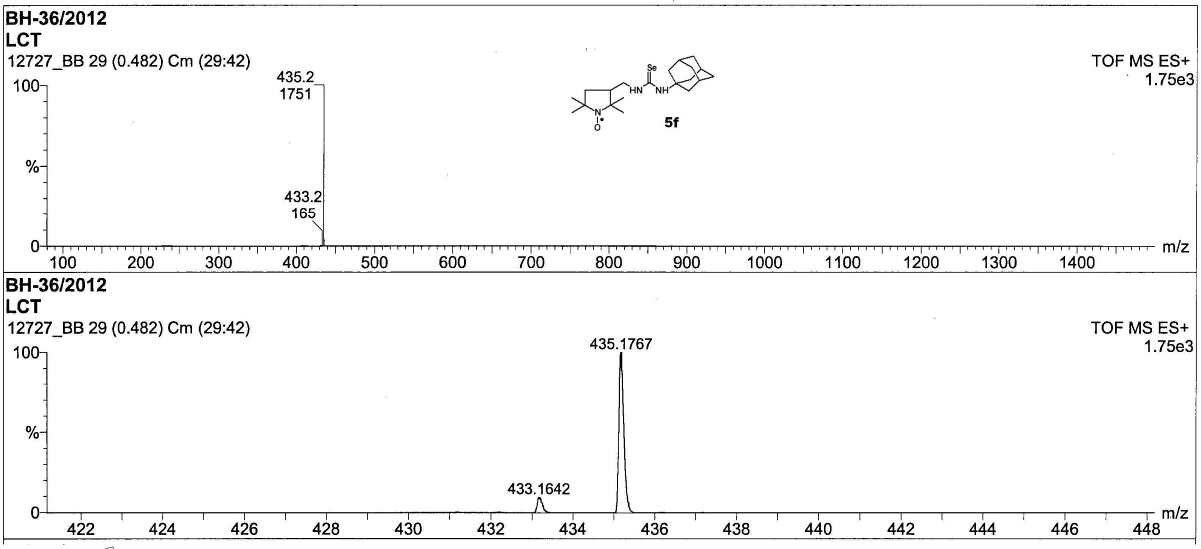

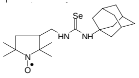

1-[(2,2,5,5-Tetramethyl-1-oxyl-3-pyrrolidinyl)methyl]-3-(1-adamantyl) selenourea, **5f**, ESI MS.

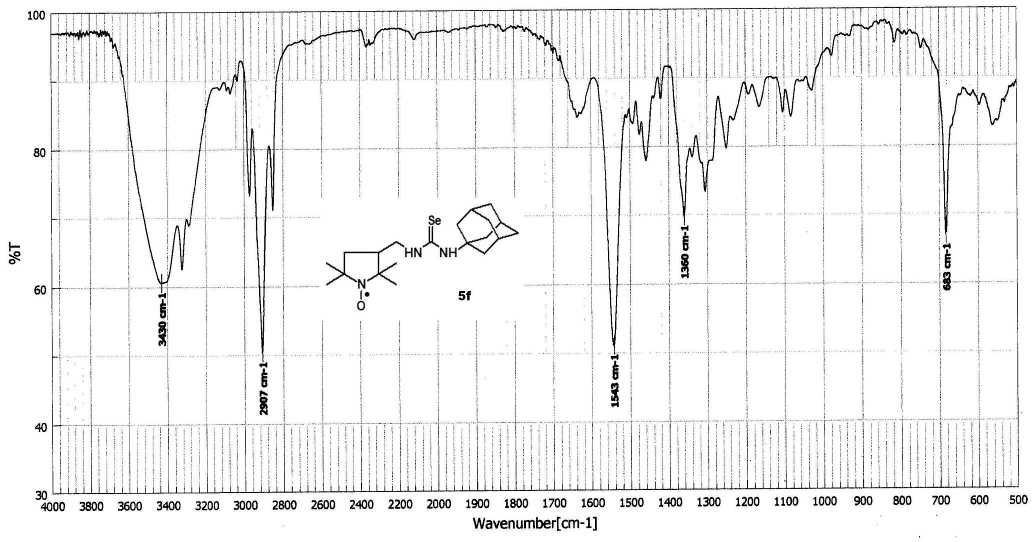


```
Sample Name        BH-36/2012 (0,4mg/270mg KBr)
Resolution         1 cm-1
Accumulation       30
Apodization        Cosine
Date/Time          112-04-13 9:14
```

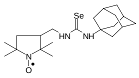

1-[(2,2,5,5-Tetramethyl-1-oxyl-3-pyrrolidinyl)methyl]-3-(1-adamantyl) selenourea, **5f**, IR.

File :C:\msdchem\1\data\maj2012\BS\BH_45_2012.D
Operator : A. Kielczewska
Acquired : 17 May 2012 12:10 using AcqMethod DI250.m
Instrument : SIS_DIP-5975B
Sample Name: BH/45/2012
Misc Info :
Vial Number: 1

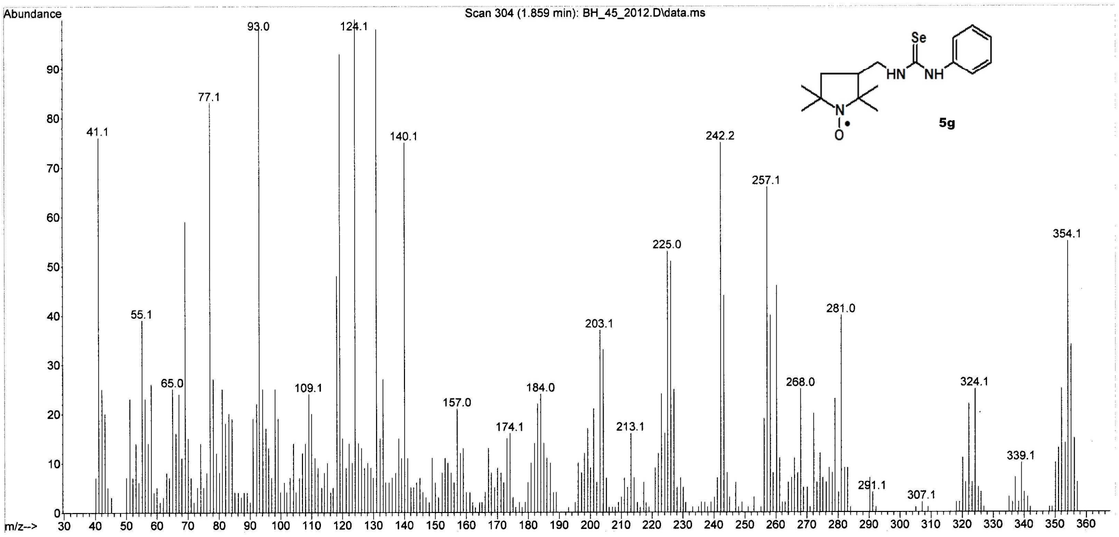

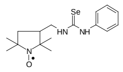

1-[(2,2,5,5-Tetramethyl-1-oxyl-3-pyrrolidinyl)methyl]-3-phenyl selenourea, **5g**, EI MS.

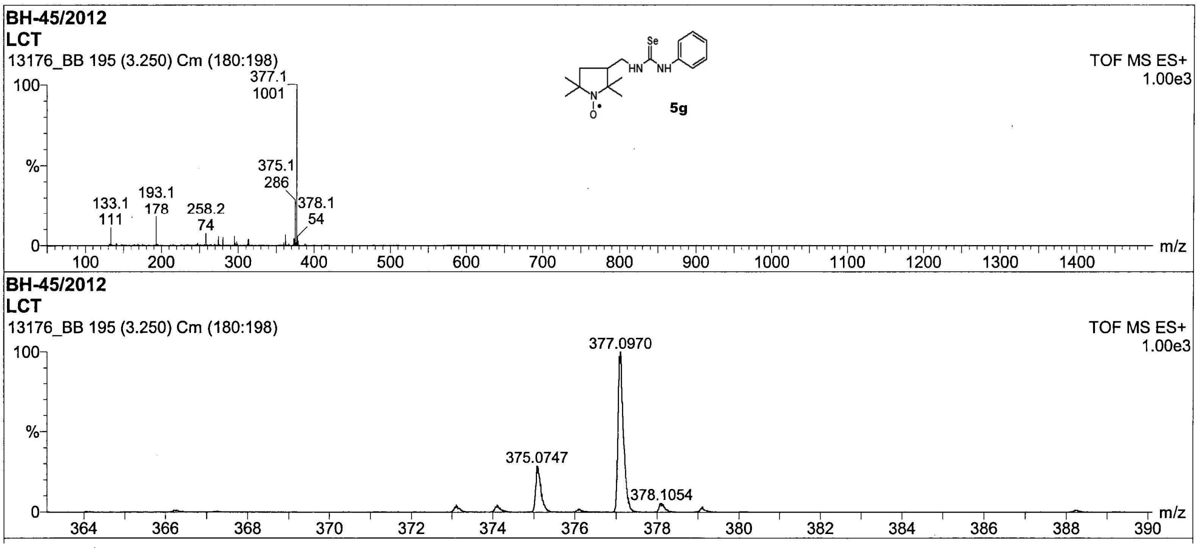

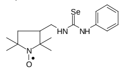

1-[(2,2,5,5-Tetramethyl-1-oxyl-3-pyrrolidinyl)methyl]-3-phenyl selenourea, **5g**, ESI MS.

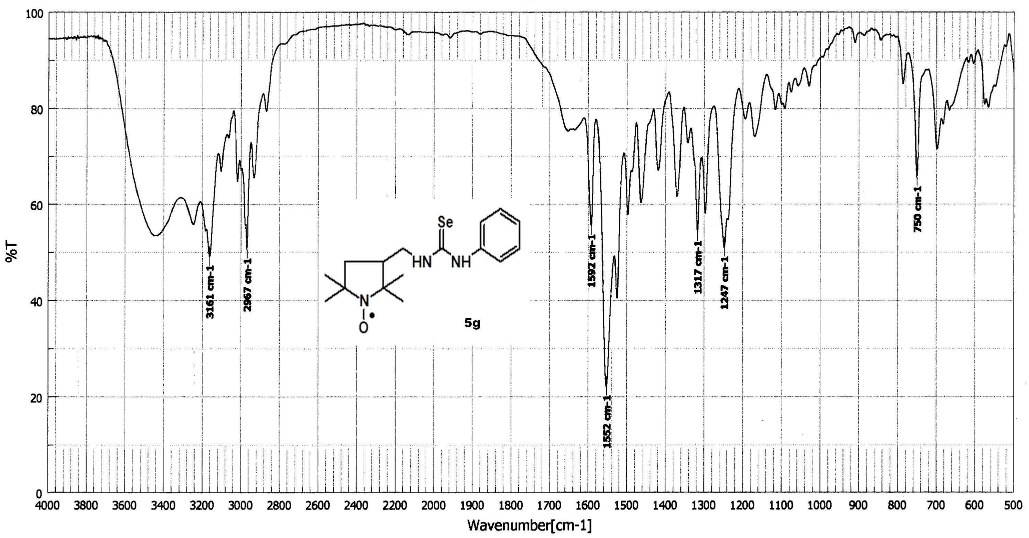

Sample Name	BH-45/2012 Fr.4/I (0,5mg/270mg KBr)
Resolution	1 cm-1
Accumulation	30
Apodization	Cosine
Date/Time	112-05-21 14:36

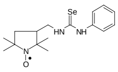

1-[(2,2,5,5-Tetramethyl-1-oxyl-3-pyrrolidinyl)methyl]-3-phenyl selenourea, **5g**, IR.

File :C:\msdchem\1\data\maj2012\BS\BH_42_2012.D
Operator : A. Kielczewska
Acquired : 17 May 2012 11:36 using AcqMethod DI250.m
Instrument : SIS_DIP-5975B
Sample Name: BH/42/2012
Misc Info :
Vial Number: 1

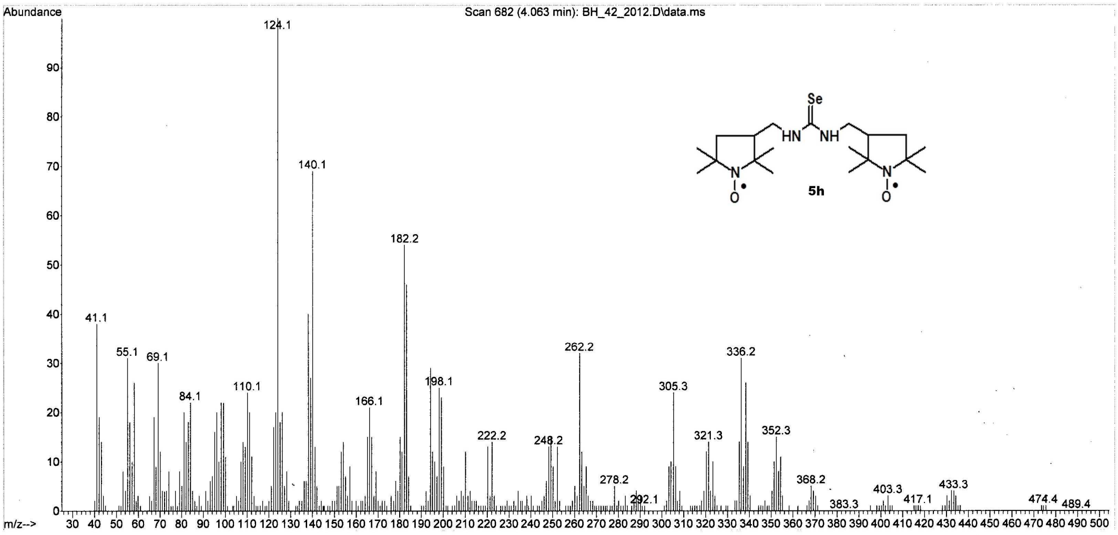

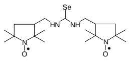 1,3-bis[(2,2,5,5-tetramethyl-1-oxyl-3-pyrrolidinyl)methyl] selenourea, **5h**, EI MS.

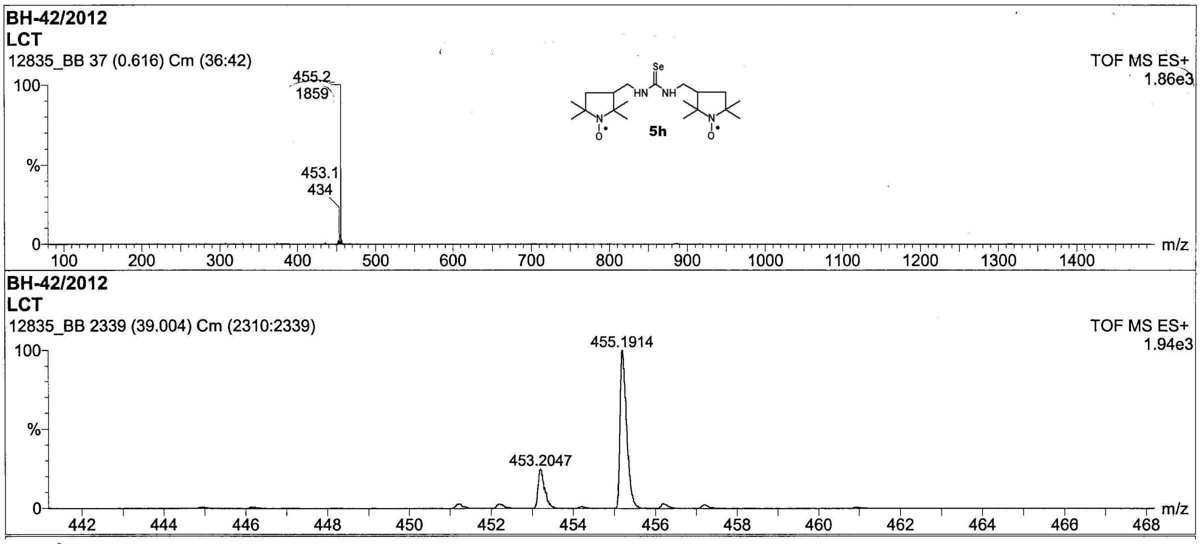

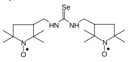

1,3-bis[(2,2,5,5-tetramethyl-1-oxyl-3-pyrrolidinyl)methyl] selenourea, **5h**, ESI MS.

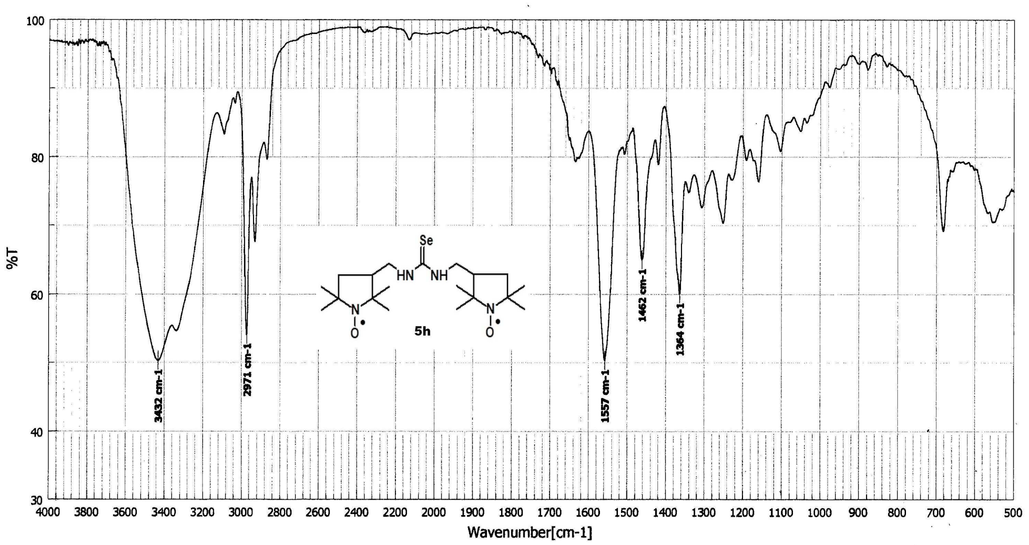

Sample Name	BH-42/2012 (0,5mg/270mg KBr)
Resolution	1 cm-1
Accumulation	30
Apodization	Cosine
Date/Time	112-05-08 10:01

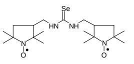

1,3-bis[(2,2,5,5-tetramethyl-1-oxyl-3-pyrrolidinyl)methyl] selenourea, **5h**, IR.

File :C:\msdchem\1\data\marzec12\BS\BH_24_2012.D
Operator :
Acquired : 13 Mar 2012 12:16 using AcqMethod DI250.m
Instrument : SIS_DIP-5975B
Sample Name: BH-24/2012
Misc Info :
Vial Number: 1

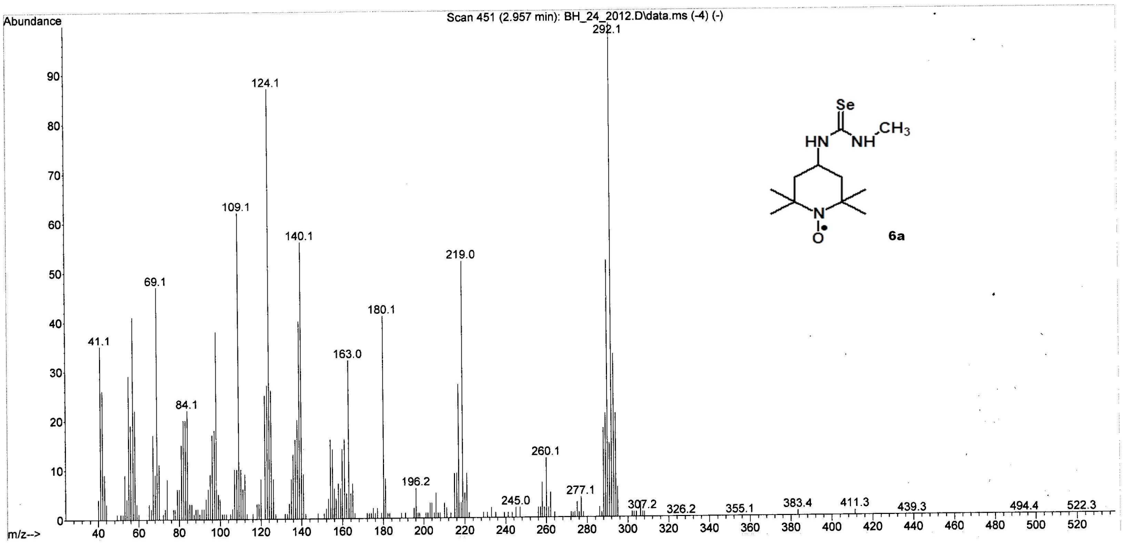

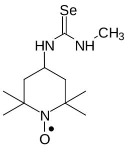

1-(2,2,6,6-Tetramethyl-1-oxyl-4-piperidynylyl)-3-methyl selenourea, **6a**, EI MS.

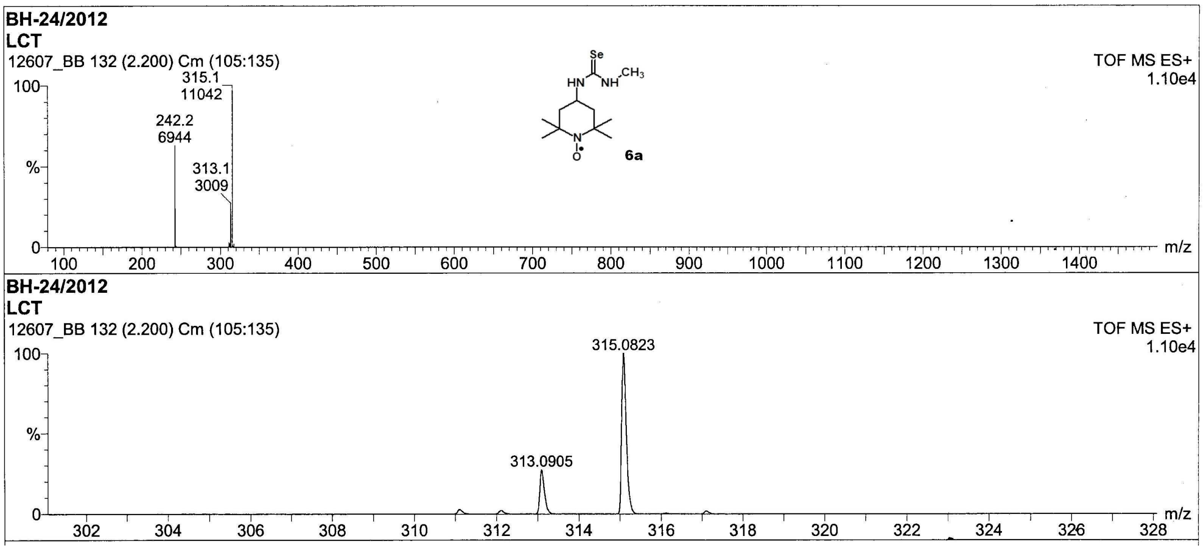

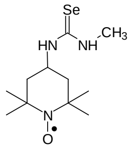

1-(2,2,6,6-Tetramethyl-1-oxyl-4-piperidynylyl)-3-methyl selenourea, **6a**, ESI MS.

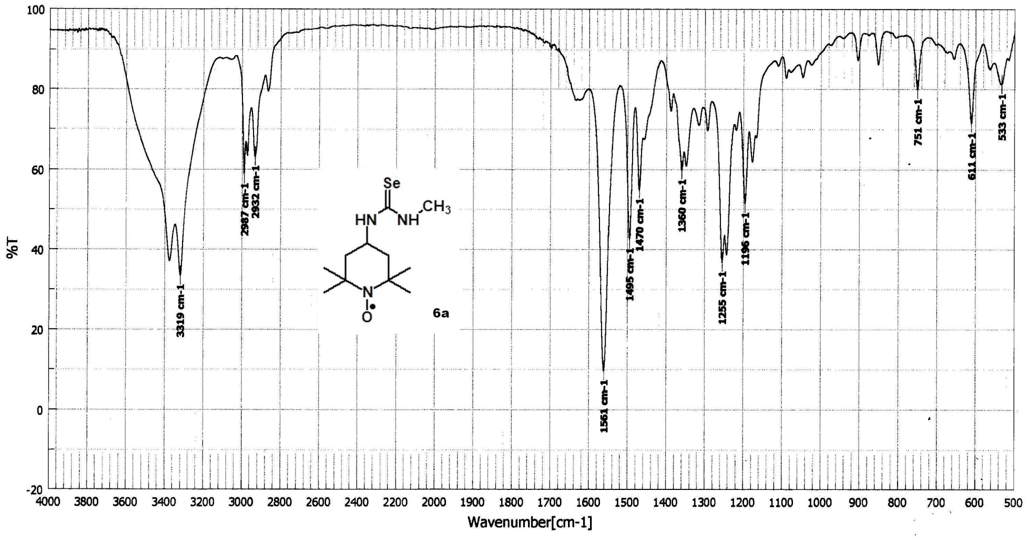

Sample Name	BH-24/2012 (0,5mg/270mg KBr)
Resolution	1 cm-1
Accumulation	30
Apodization	Cosine
Date/Time	112-03-13 14:10

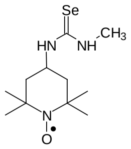

1-(2,2,6,6-Tetramethyl-1-oxyl-4-piperidynylyl)-3-methyl selenourea, **6a**, IR.

File :C:\msdchem\1\data\marzec12\BS\BH_9_2012.D
Operator : A Kielczewska
Acquired : 7 Mar 2012 12:35 using AcqMethod DI250.m
Instrument : SIS_DIP-5975B
Sample Name: BH/9/2012
Misc Info :
Vial Number: 1

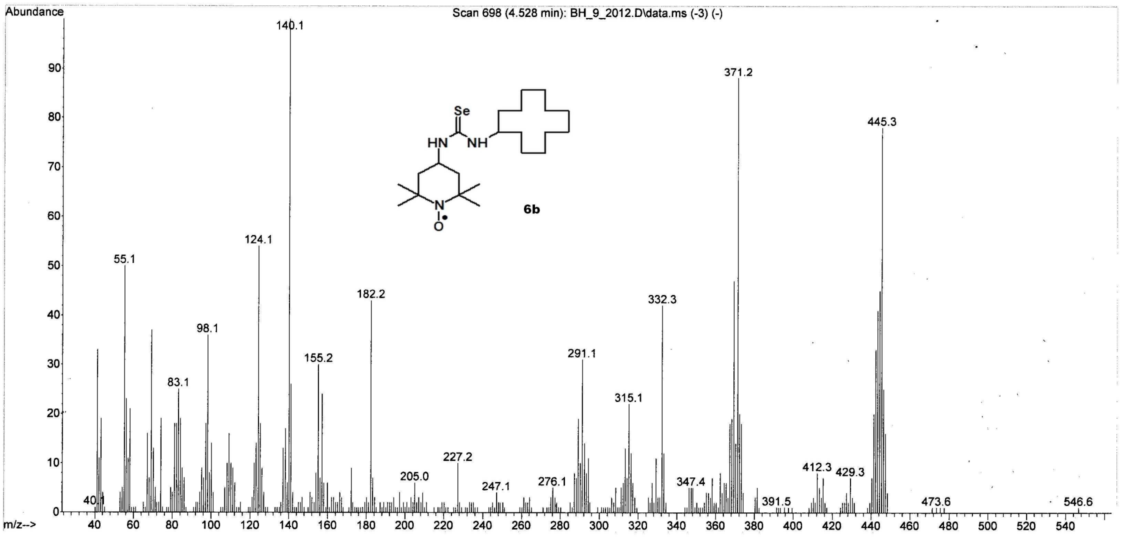

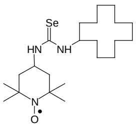

1-(2,2,6,6-Tetramethyl-1-oxyl-4-piperidynylyl)-3-(1-cyclododecyl) selenourea, **6b**, EI MS.

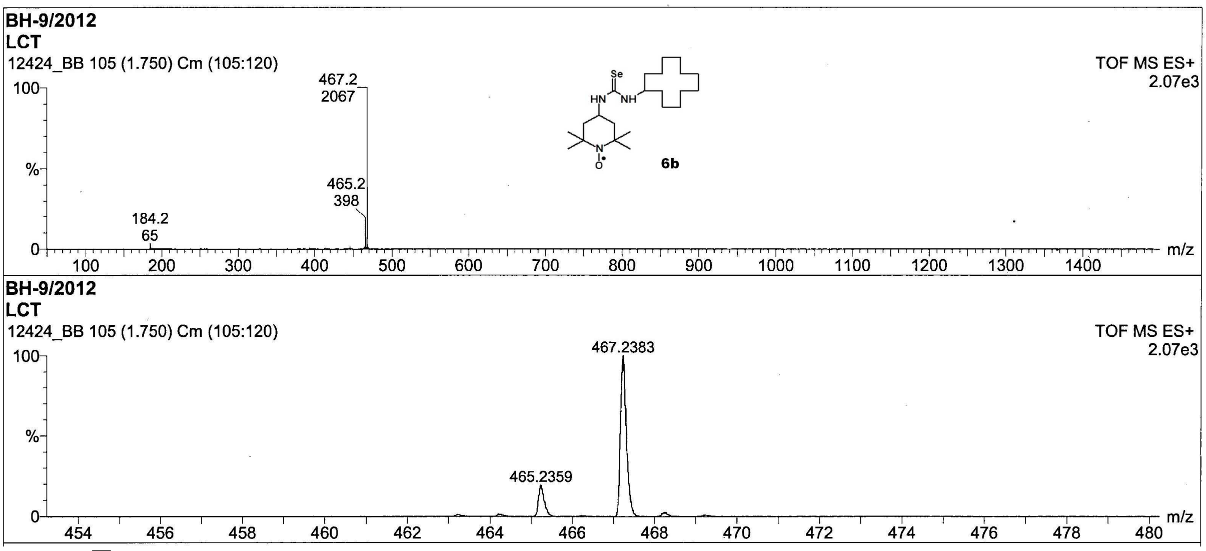

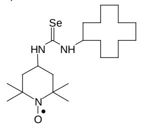

1-(2,2,6,6-Tetramethyl-1-oxyl-4-piperidynylyl)-3-(1-cyclododecyl) selenourea, **6b**, ESI MS.

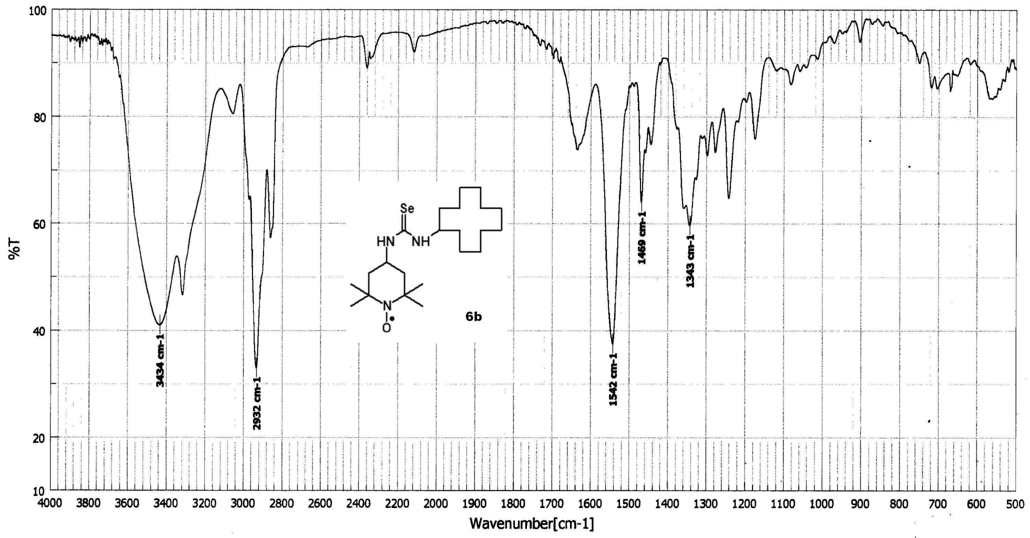


```
Sample Name      BH-9/2012  (0,5mg/270mg KBr)
Resolution       1 cm-1
Accumulation     30
Apodization      Cosine
Date/Time        112-01-25 13:15
```

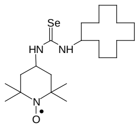

1-(2,2,6,6-Tetramethyl-1-oxyl-4-piperidynylyl)-3-(1-cyclododecyl) selenourea, **6b**, IR.

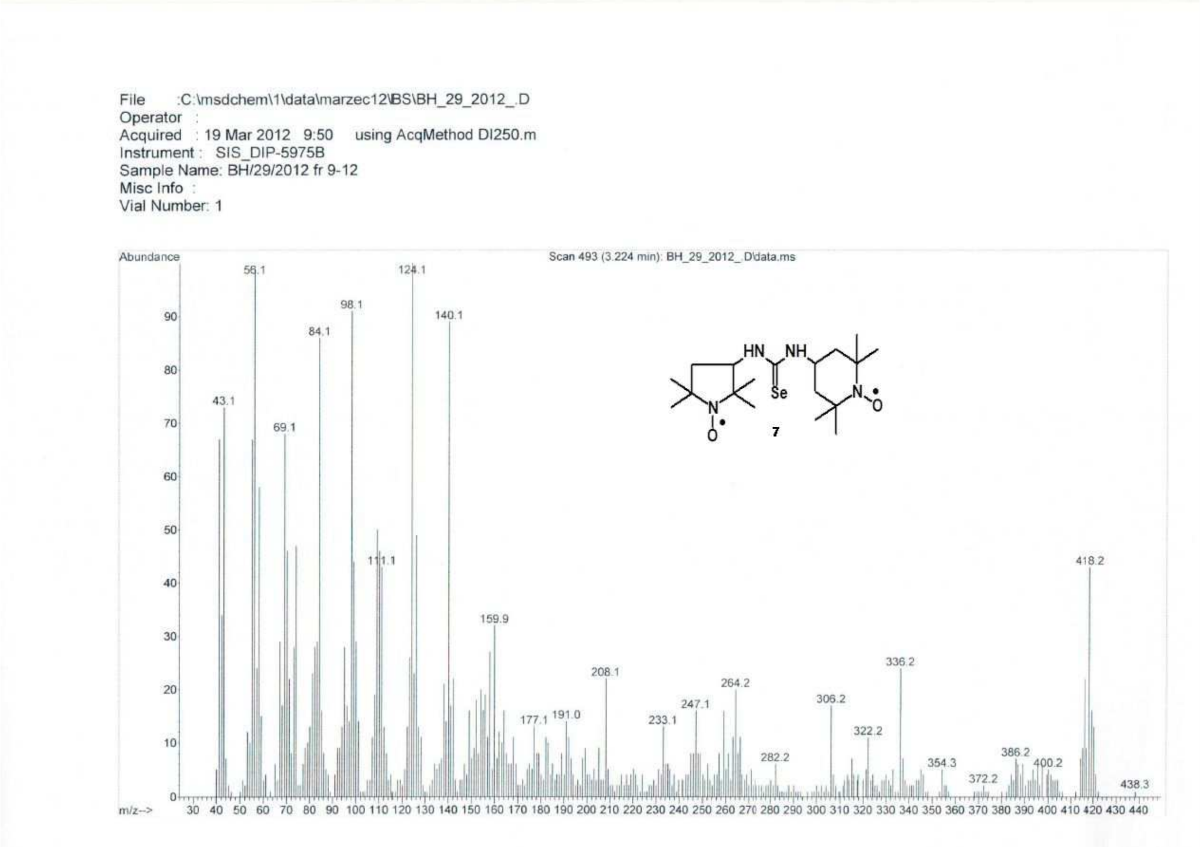

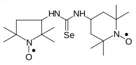 1-(2,2,5,5-Tetramethyl-1-oxyl-3-pyrrolidinyl)-3-(2,2,6,6-tetramethyl-1-oxyl-4-piperidynylyl) selenourea, **7**, EI MS.

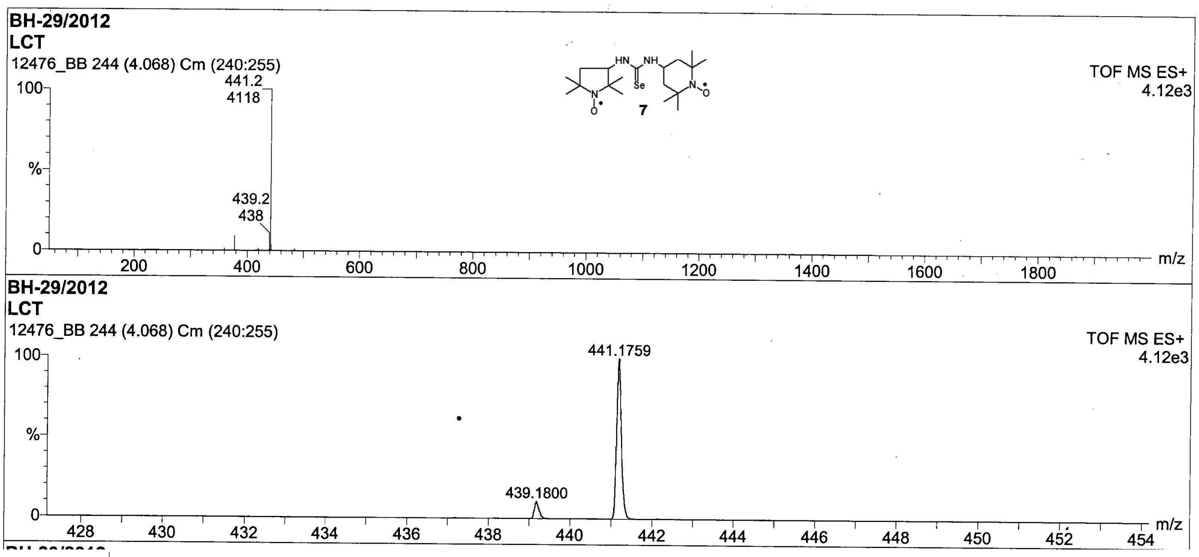

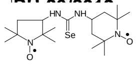 1-(2,2,5,5-Tetramethyl-1-oxyl-3-pyrrolidinyl)-3-(2,2,6,6-tetramethyl-1-oxyl-4-piperidynylyl) selenourea, **7**, ESI MS.

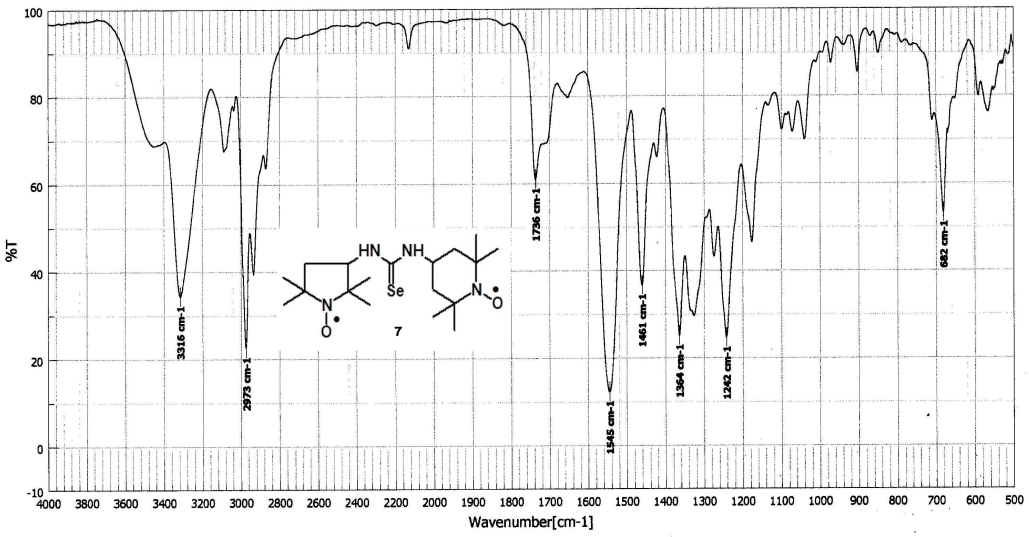

Sample Name	BH-29/2012 Fr 9-12 (film na płytce KBr)
Resolution	1 cm-1
Accumulation	30
Apodization	Cosine
Date/Time	112-03-19 11:44

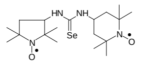
1-(2,2,5,5-Tetramethyl-1-oxyl-3-pyrrolidinyl)-3-(2,2,6,6-tetramethyl-1-oxyl-4-piperidynylyl) selenourea, **7**, IR.

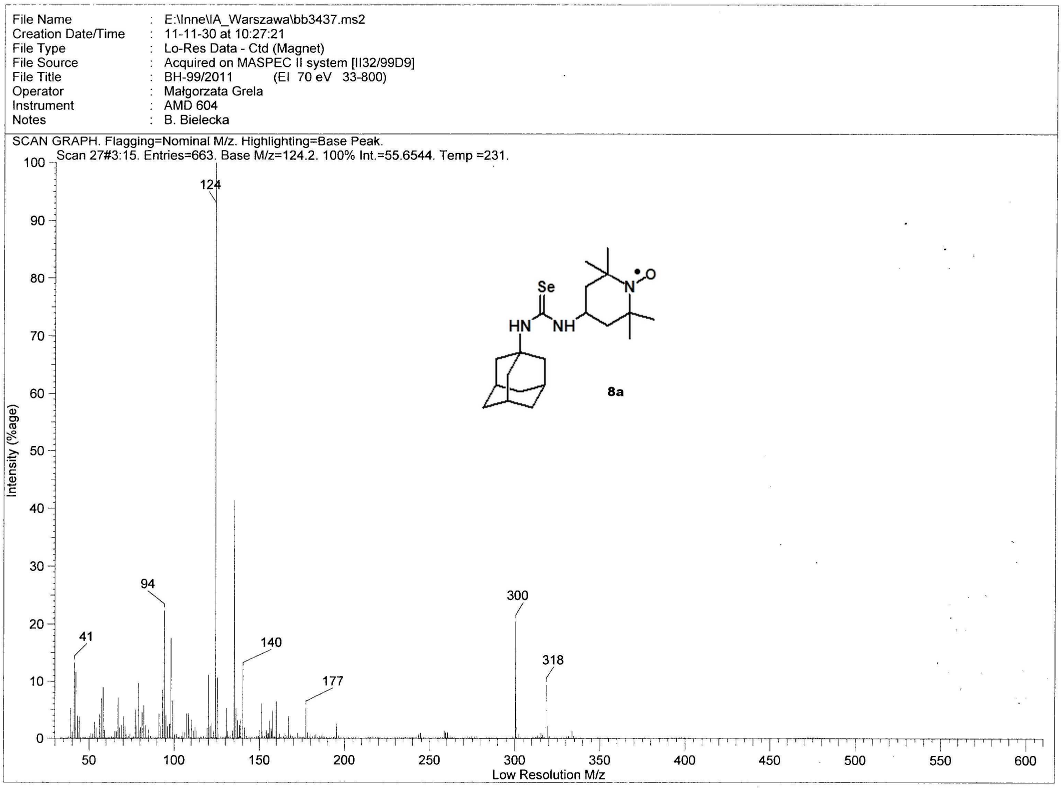

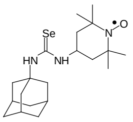

1-(1-Adamantyl)-3-(2,2,6,6-tetramethyl-1-oxyl-4-piperidynylyl) selenourea, **8a**, EI MS.

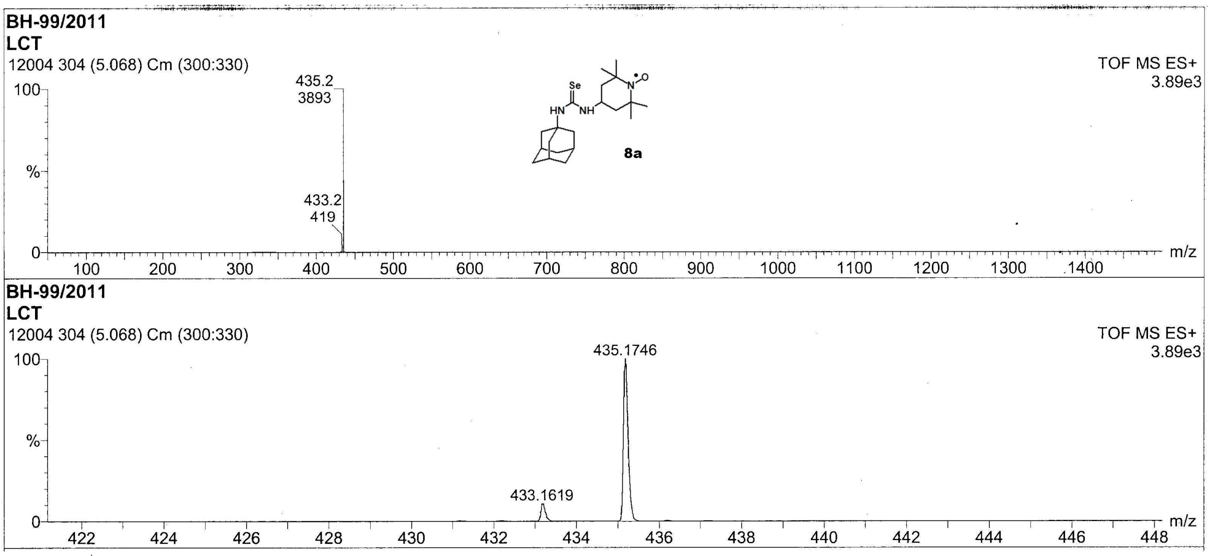

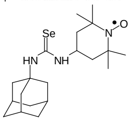

1-(1-Adamantyl)-3-(2,2,6,6-tetramethyl-1-oxyl-4-piperidynylyl) selenourea, **8a**, ESI MS.

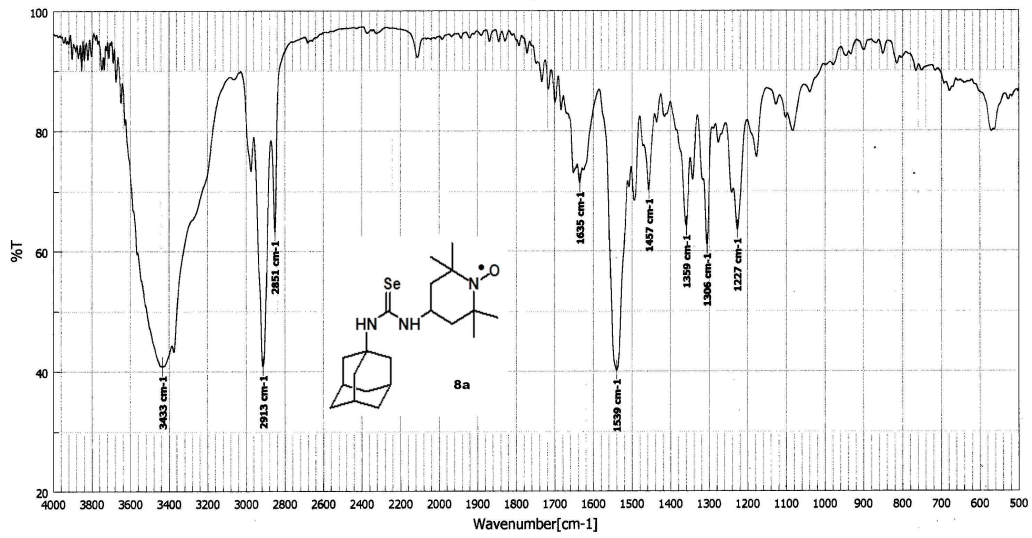

Sample Name	BH-99/2011 (0,4mg/270mg KBr)
Resolution	1 cm-1
Accumulation	30
Apodization	Cosine
Date/Time	111-11-14 11:21

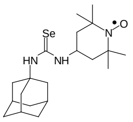

1-(1-Adamantyl)-3-(2,2,6,6-tetramethyl-1-oxyl-4-piperidynylyl) selenourea, **8a**, IR.

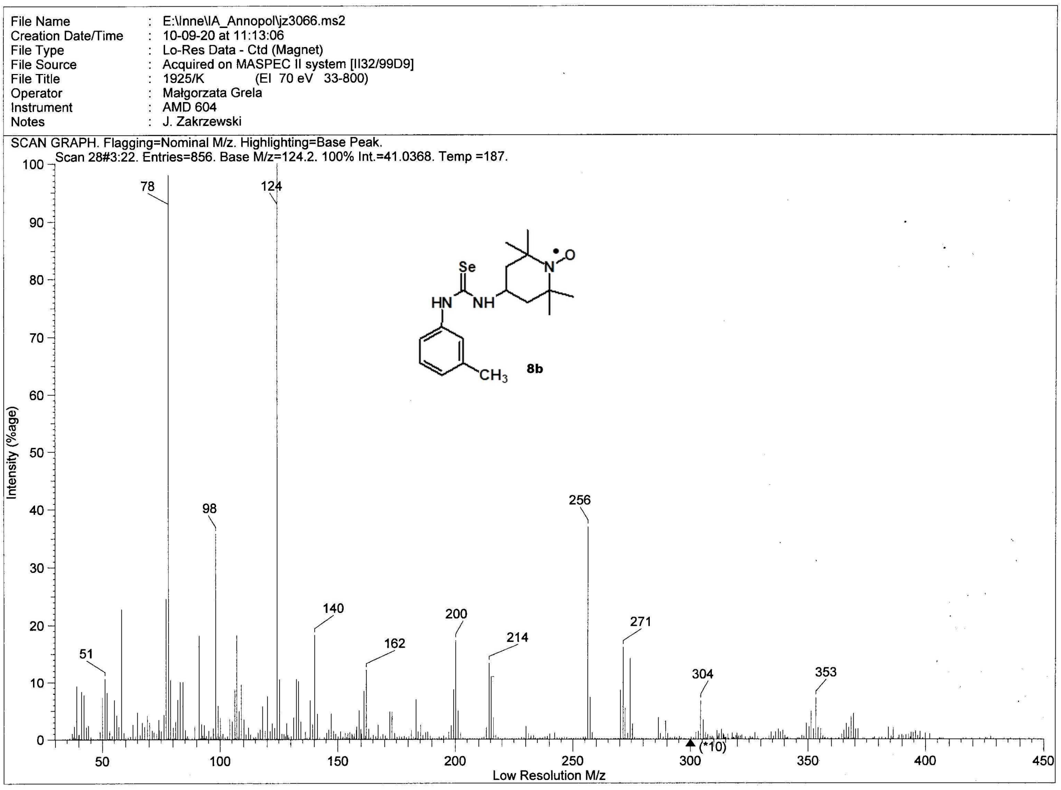

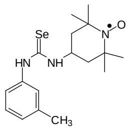

1-(3-Methylphenyl)-3-(2,2,6,6-tetramethyl-1-oxyl-4-piperidynylyl) selenourea, **8b**, EI MS.

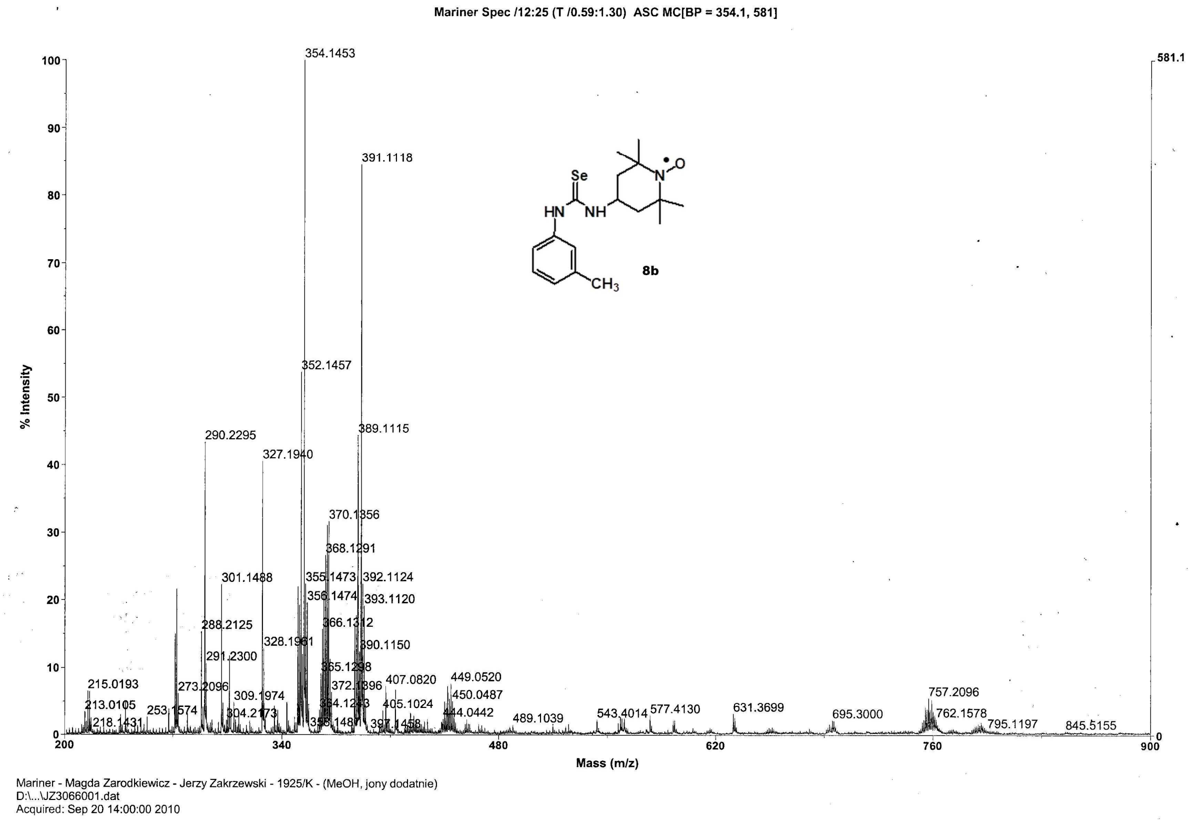

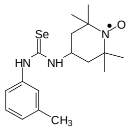

1-(3-Methylphenyl)-3-(2,2,6,6-tetramethyl-1-oxyl-4-piperidynylyl) selenourea, **8b**, ESI MS.

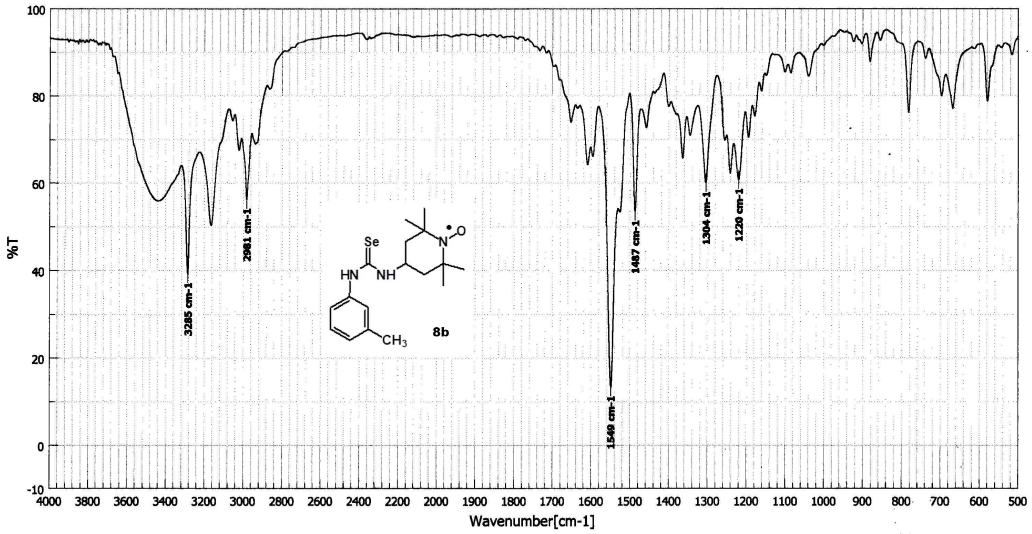


```
Sample Name          1925/K   (0,25mg/270mg KBr)
Resolution           1 cm-1
Accumulation         30
Apodization          Cosine
Date/Time            110-09-15 11:31
```

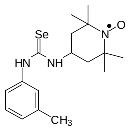

1-(3-Methylphenyl)-3-(2,2,6,6-tetramethyl-1-oxyl-4-piperidynylyl) selenourea, **8b**, IR.

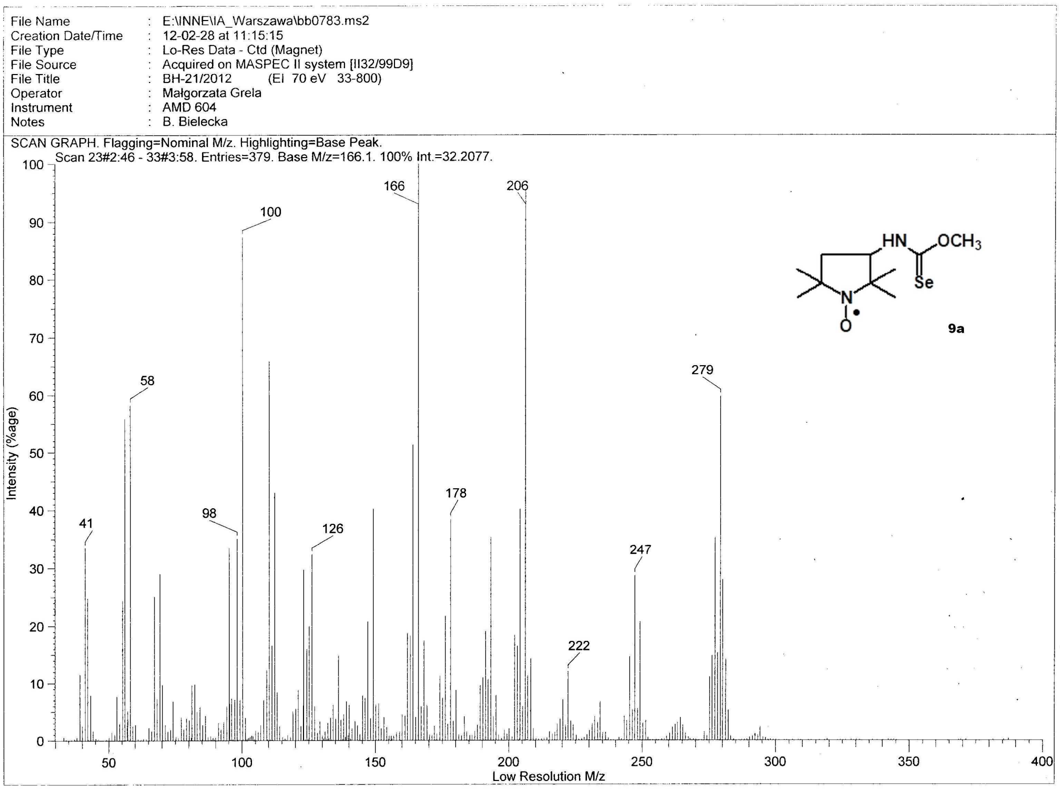

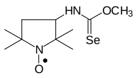

Methyl *N*-(2,2,5,5-tetramethyl-1-oxyl-3-pyrrolidinyl) selenonocarbamate, **9a**, EI MS.

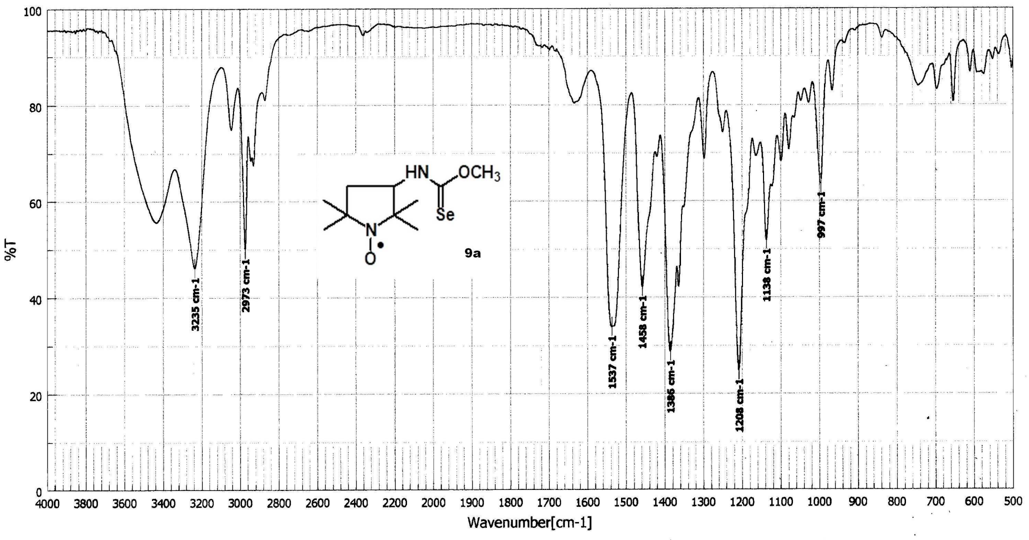


```
Sample Name       BH-21/2012  (0,5mg/270mg KBr)
Resolution        1 cm-1
Accumulation      30
Apodization       Cosine
Date/Time         112-02-27 11:33
```

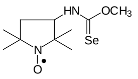

Methyl *N*-(2,2,5,5-tetramethyl-1-oxyl-3-pyrrolidinyl) selenonocarbamate, **9a**, IR.

File :C:\msdchem\1\data\luty2012\BS\BH_22_2012.D
Operator : E. Lacka
Acquired : 28 Feb 2012 10:49 using AcqMethod DI250.m
Instrument : SIS_DIP-5975B
Sample Name: BH/22/2012
Misc Info :
Vial Number: 1

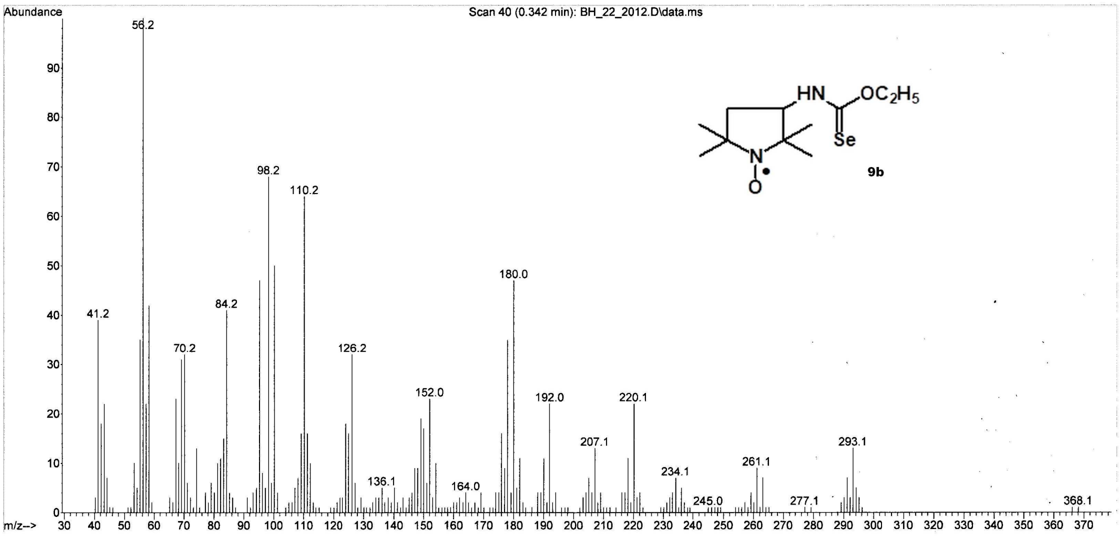

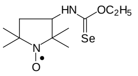

Ethyl *N*-(2,2,5,5-tetramethyl-1-oxyl-3-pyrrolidinyl) selenonocarbamate, **9b**, EI MS.

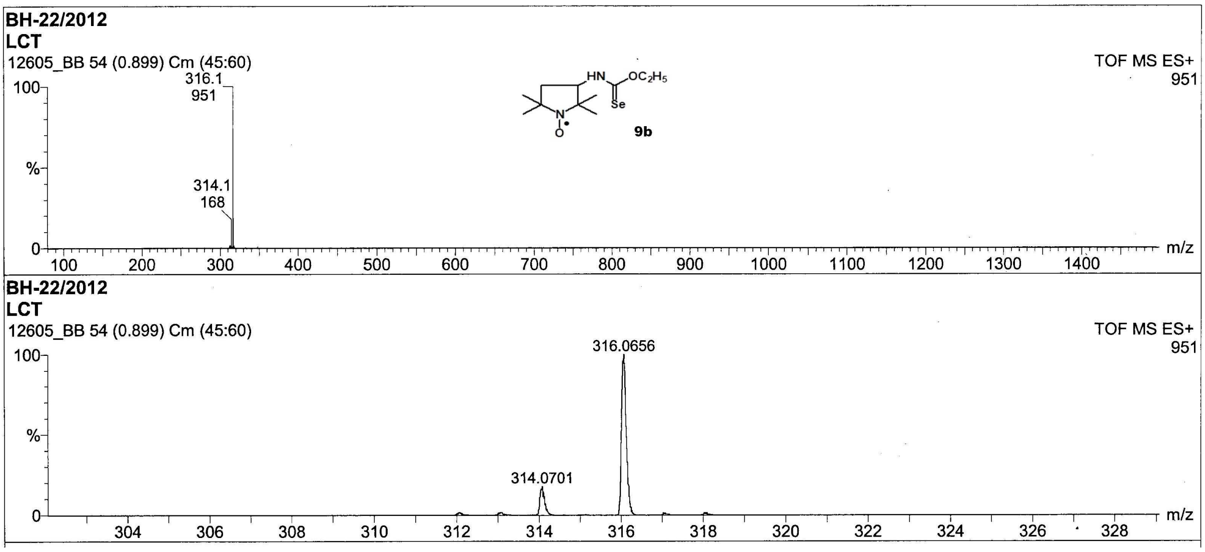

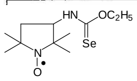

Ethyl *N*-(2,2,5,5-tetramethyl-1-oxyl-3-pyrrolidinyl) selenonocarbamate, **9b**, ESI MS.

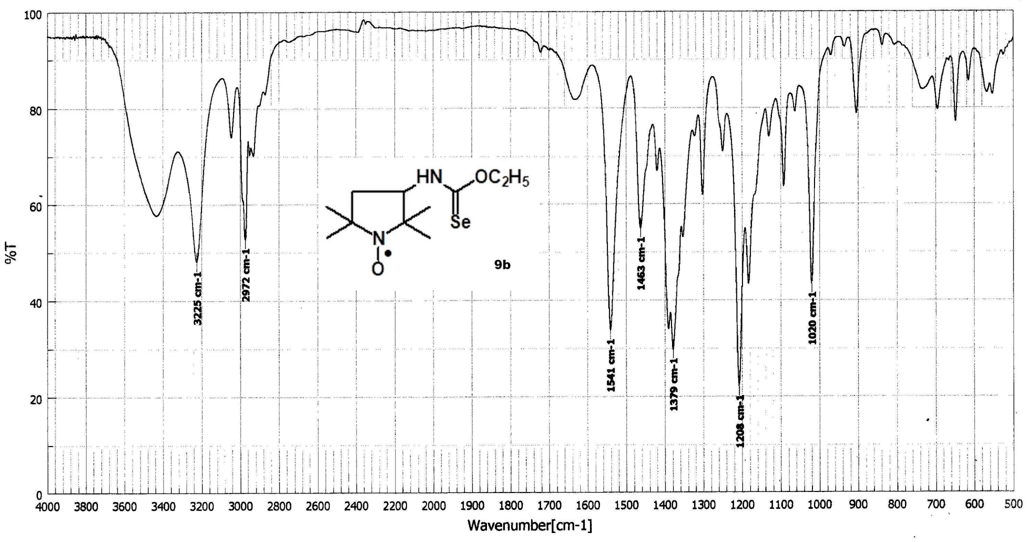

Sample Name	BH-22/2012 (0,5mg/270mg KBr)
Resolution	1 cm-1
Accumulation	30
Apodization	Cosine
Date/Time	112-02-27 11:06

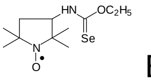

Ethyl *N*-(2,2,5,5-tetramethyl-1-oxyl-3-pyrrolidinyl) selenonocarbamate, **9b**, IR.

File :C:\msdchem\1\data\maj2012\BS\BH_50_2012.D
Operator : A Kielczewska
Acquired : 18 May 2012 9:23 using AcqMethod DI250.m
Instrument : SIS_DIP-5975B
Sample Name: BH/50/2012
Misc Info :
Vial Number: 1

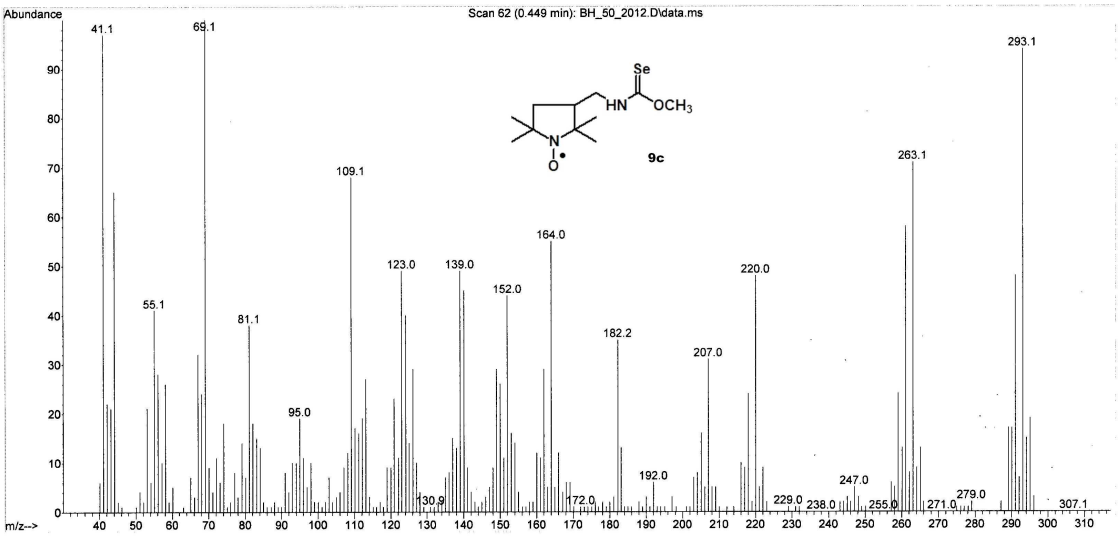

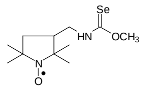

Methyl *N*-((2,2,5,5-tetramethyl-1-oxyl-3-pyrrolidinyl)methyl) selenonocarbamate, **9c**, EI MS.

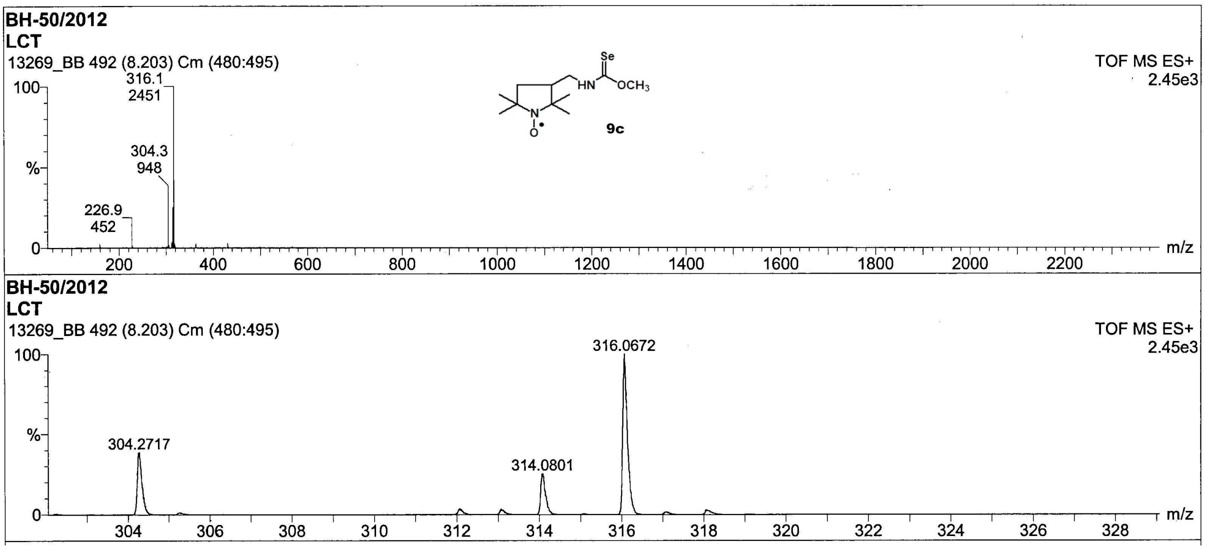

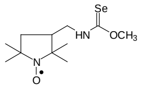

Methyl *N*-((2,2,5,5-tetramethyl-1-oxyl-3-pyrrolidinyl)methyl) selenonocarbamate, **9c**, ESI MS.

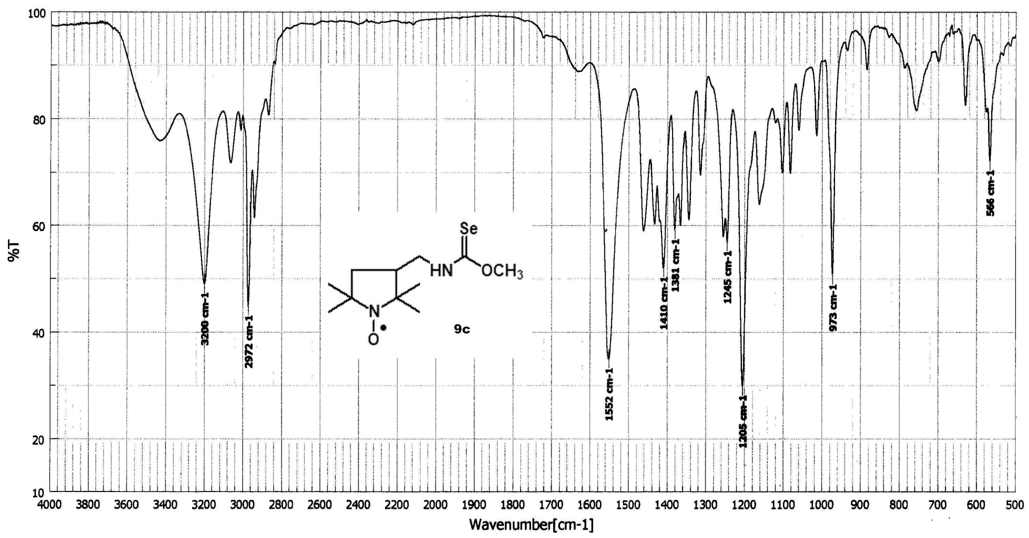

Sample Name	BH-50/2012 (0,5mg/270mg KBr)
Resolution	1 cm-1
Accumulation	30
Apodization	Cosine
Date/Time	112-05-22 9:34

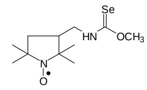

Methyl *N*-((2,2,5,5-tetramethyl-1-oxyl-3-pyrrolidinyl)methyl) selenonocarbamate, **9c**, IR.

File :C:\msdchem\1\data\maj2012\BS\BH_51_2012.D
Operator : A Kielczewska
Acquired : 18 May 2012 9:56 using AcqMethod DI250.m
Instrument : SIS_DIP-5975B
Sample Name: BH/51/2012
Misc Info :
Vial Number: 1

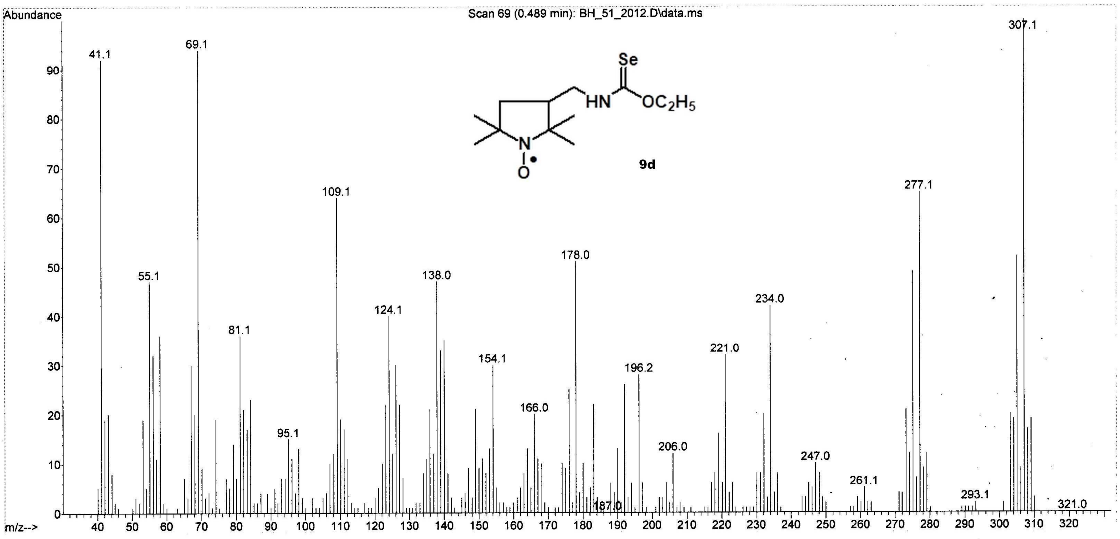

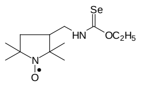
Ethyl *N*-((2,2,5,5-tetramethyl-1-oxyl-3-pyrrolidinyl)methyl) selenonocarbamate, **9d**, EI MS.

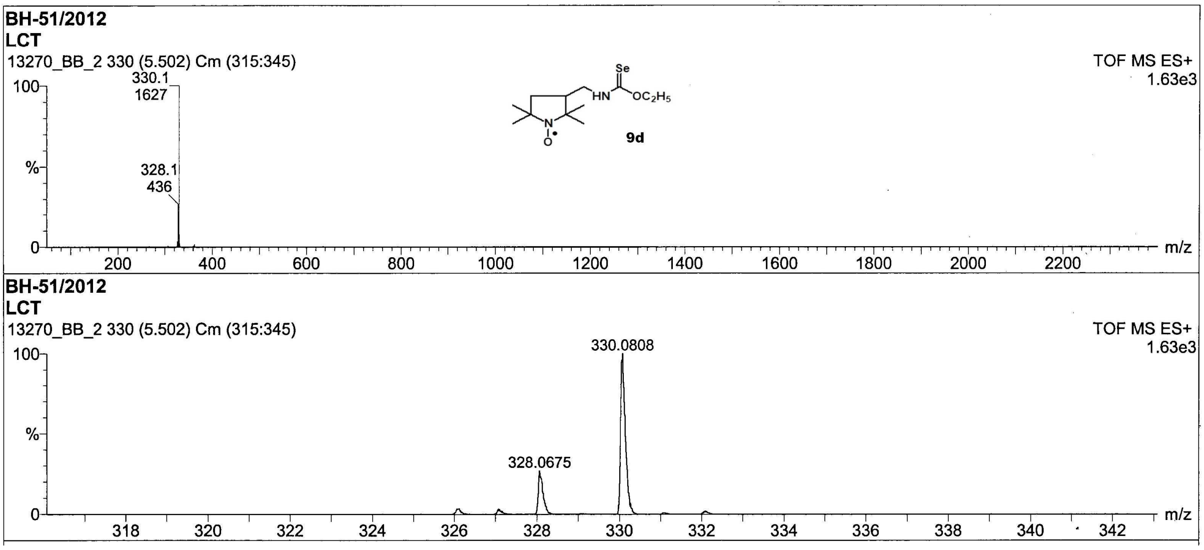

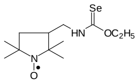

Ethyl *N*-((2,2,5,5-tetramethyl-1-oxyl-3-pyrrolidinyl)methyl) selenonocarbamate**, 9d**, ESI MS.

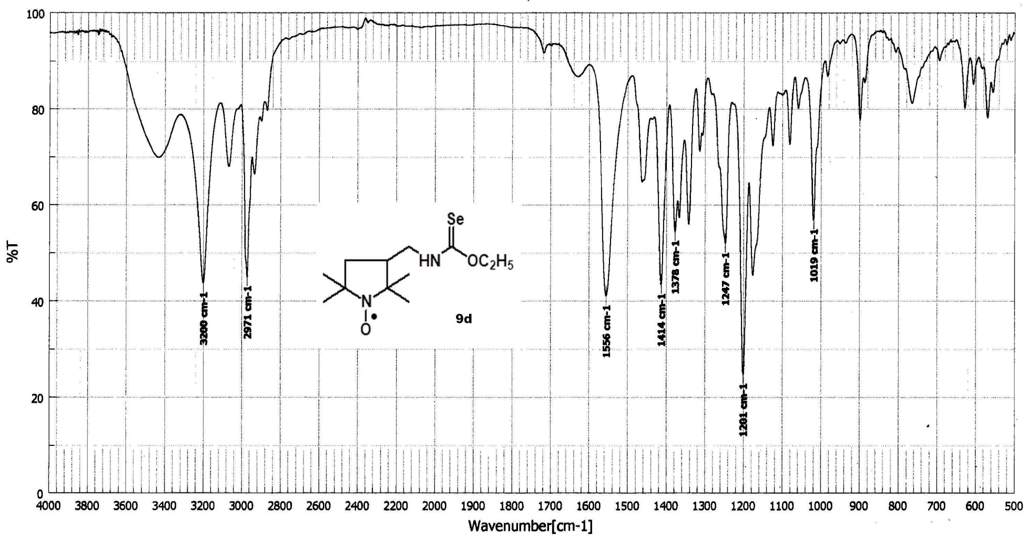


```
Sample Name          BH-51/2012  (0,5mg/270mg KBr)
Resolution           1 cm-1
Accumulation         30
Apodization          Cosine
Date/Time            112-05-22 10:01
```

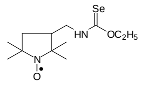

Ethyl *N*-((2,2,5,5-tetramethyl-1-oxyl-3-pyrrolidinyl)methyl) selenonocarbamate**, 9d**, IR.

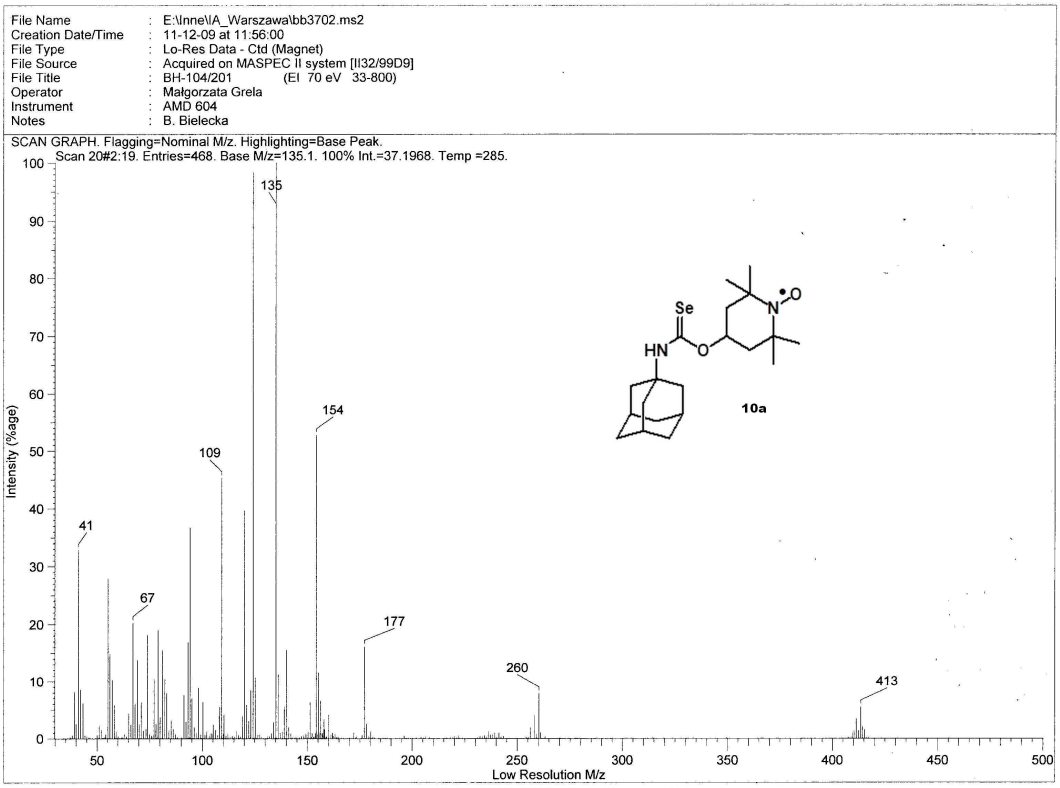

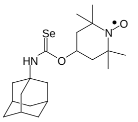

2,2,6,6-Tetramethyl-1-oxyl-4-piperidinyl *N*-(1-adamantyl) selenonocarbamate, **10a**, EI MS.

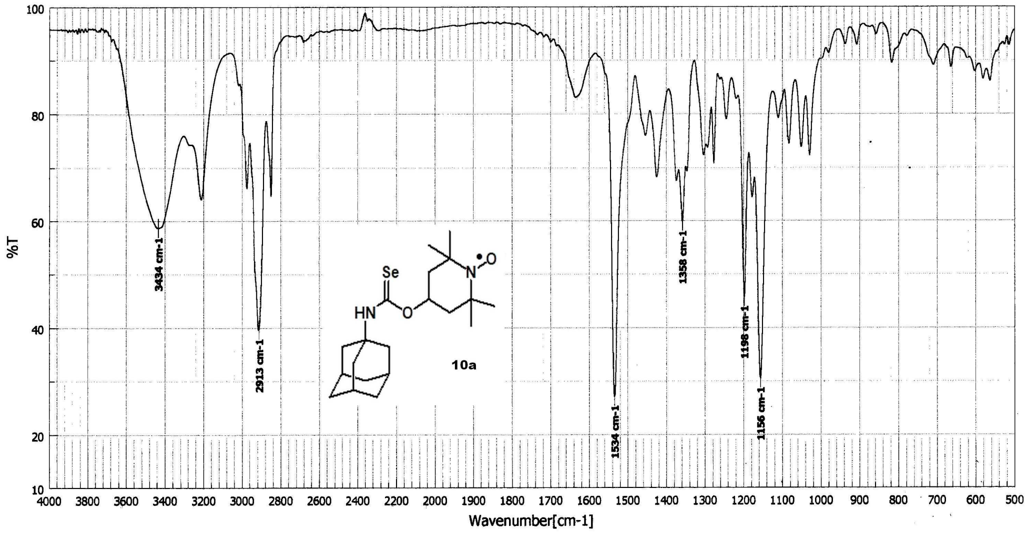


```
Sample Name       BH-104/2011  (0,5mg/270mg KBr)
Resolution        1 cm-1
Accumulation      30
Apodization       Cosine
Date/Time         111-12-05 12:44
```

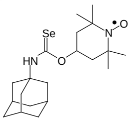

2,2,6,6-Tetramethyl-1-oxyl-4-piperidinyl *N*-(1-adamantyl) selenonocarbamate, **10a**, IR.

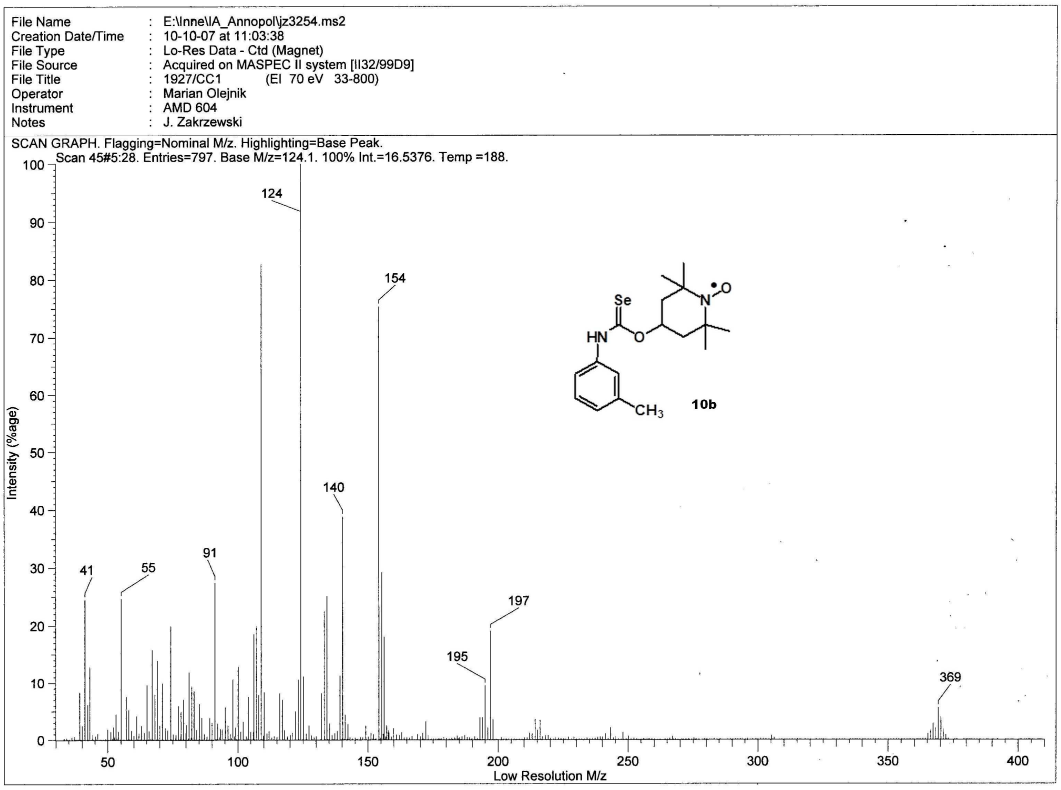

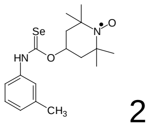

2,2,6,6-Tetramethyl-1-oxyl-4-piperidinyl *N*-(3-methylphenyl) selenonocarbamate, **10b**, EI MS.

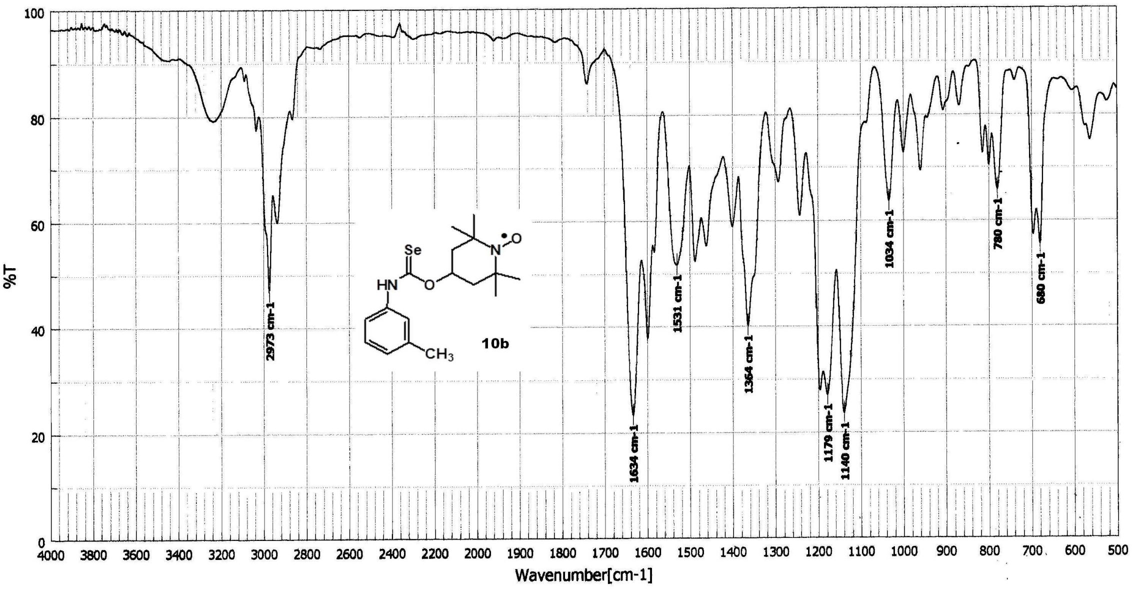


```
Sample Name       1927/CC1  (film na płytce KBr)
Resolution        1 cm-1
Accumulation      30
Apodization       Cosine
Date/Time         110-10-06 9:53
```

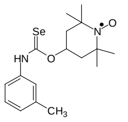

2,2,6,6-Tetramethyl-1-oxyl-4-piperidinyl *N*-(3-methylphenyl) selenonocarbamate, **10b**, IR.

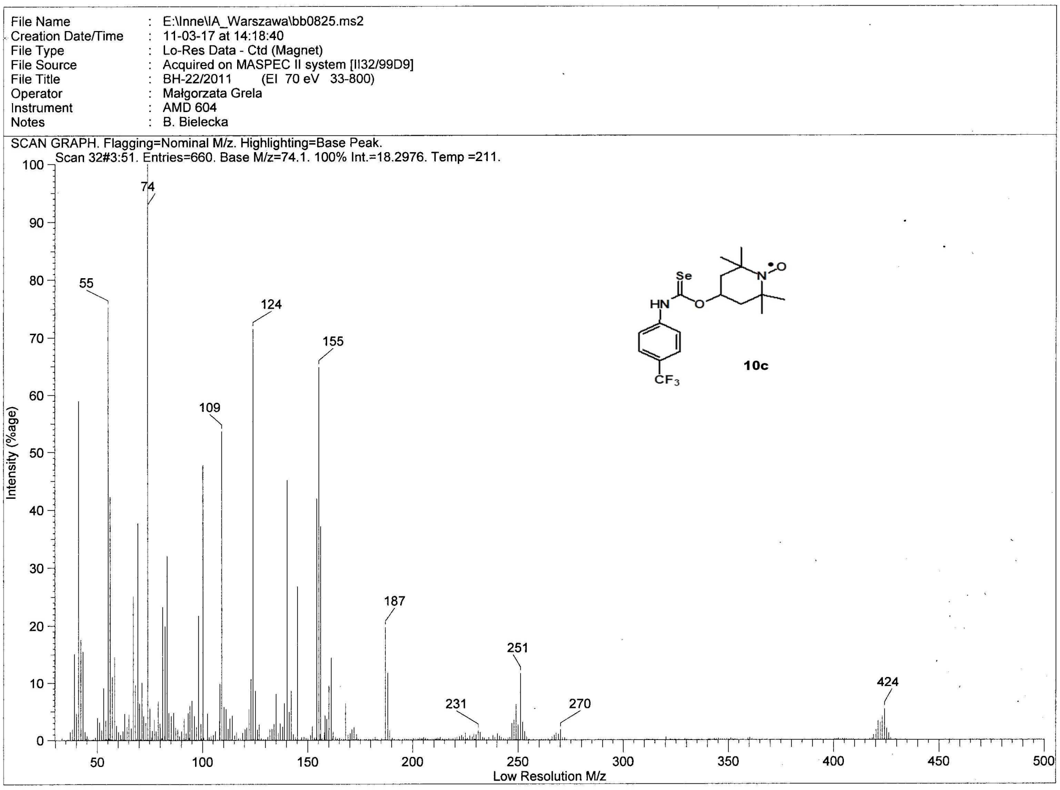

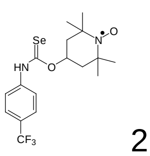

2,2,6,6-Tetramethyl-1-oxyl-4-piperidinyl *N*-(4-(trifluoromethyl)phenyl) selenonocarbamate, **10c**, EI MS.

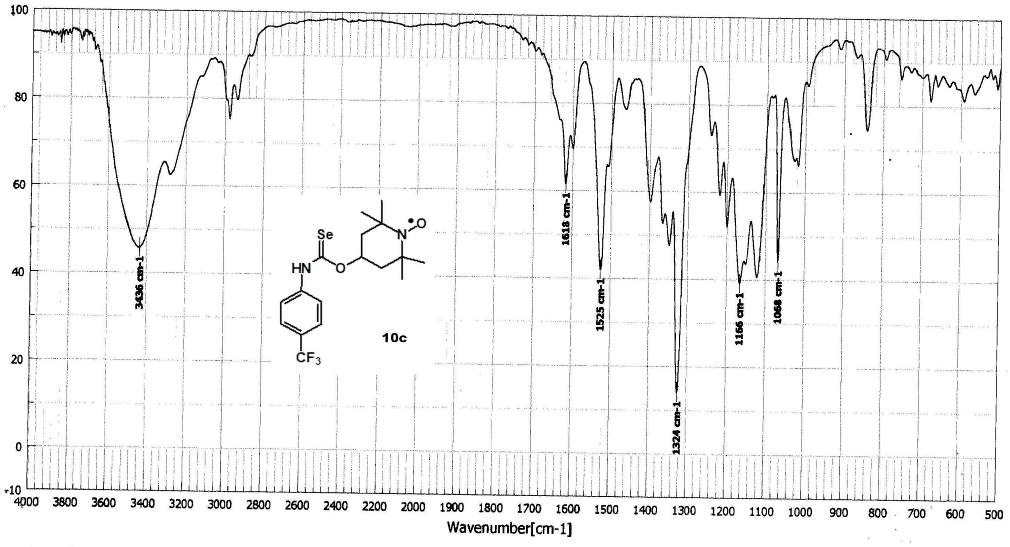

Sample Name	BH-22/2011 (0,5mg/270mg KBr)
Resolution	1 cm-1
Accumulation	30
Apodization	Cosine
Date/Time	111-03-16 12:42

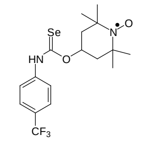

2,2,6,6-Tetramethyl-1-oxyl-4-piperidinyl *N*-(4-(trifluoromethyl)phenyl) selenonocarbamate, **10c**, IR.

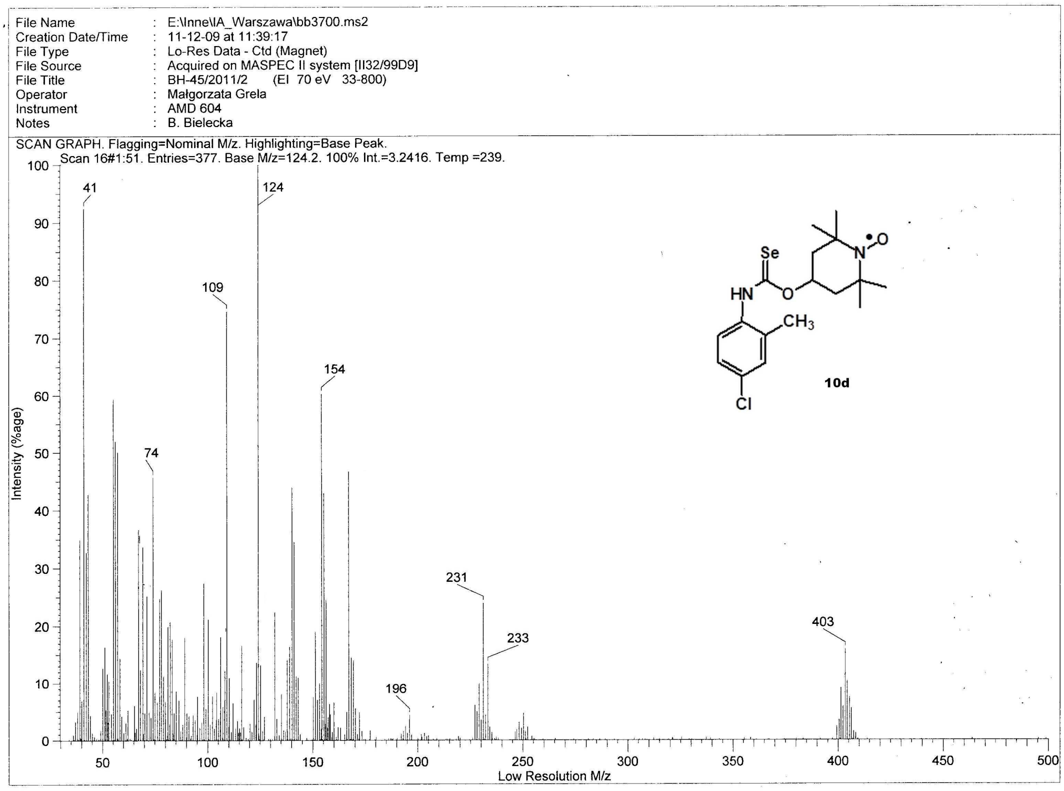

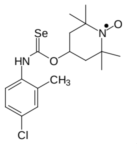

2,2,6,6-Tetramethyl-1-oxyl-4-piperidinyl *N*-(4-chloro-2-methylphenyl) selenonocarbamate, **10d**, EI MS.

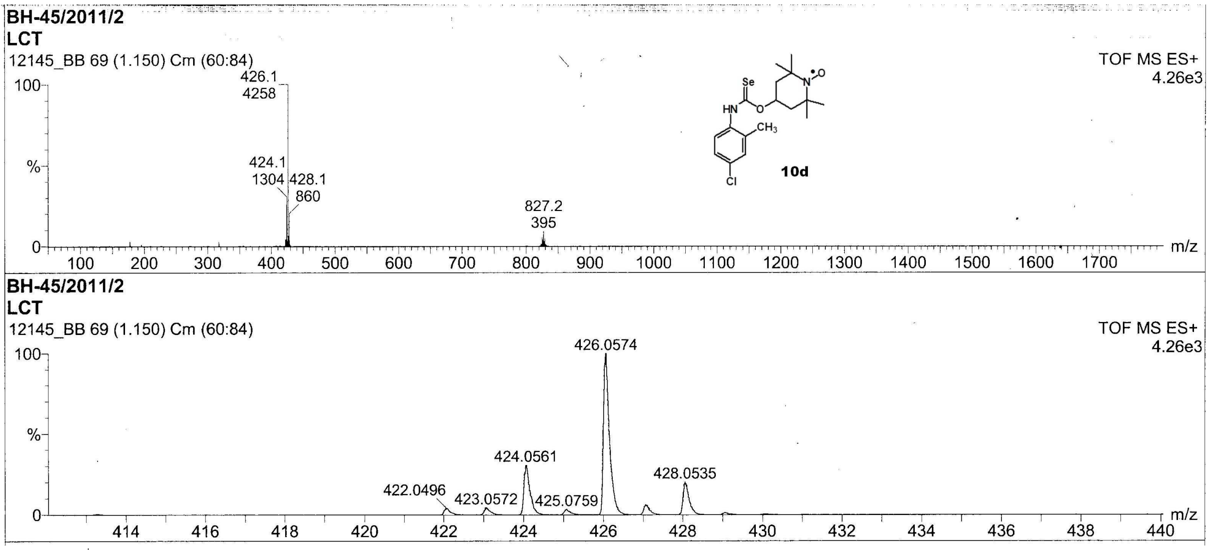

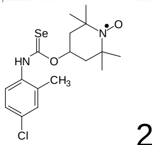

2,2,6,6-Tetramethyl-1-oxyl-4-piperidinyl *N*-(4-chloro-2-methylphenyl) selenonocarbamate, **10d**, ESI MS.

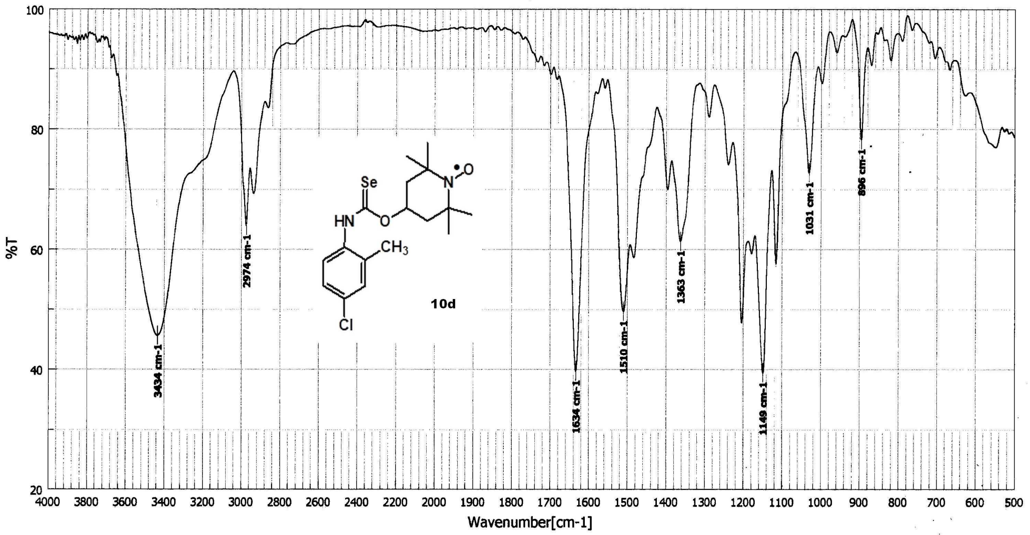

Sample Name	BH-45/2011/2 (0,5mg/270mg KBr)
Resolution	1 cm-1
Accumulation	30
Apodization	Cosine
Date/Time	111-12-12 12:26

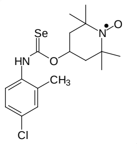

2,2,6,6-Tetramethyl-1-oxyl-4-piperidinyl *N*-(4-chloro-2-methylphenyl) selenonocarbamate, **10d**, IR.
